# Novel targetable FGFR2 and FGFR3 alterations in glioblastoma associate with aggressive phenotype and distinct gene expression programs

**DOI:** 10.1186/s40478-021-01170-1

**Published:** 2021-04-14

**Authors:** Maria-Magdalena Georgescu, Mohammad Zahidul Islam, Yan Li, James Traylor, Anil Nanda

**Affiliations:** 1 NeuroMarkers PLLC, Houston, TX 77025 USA; 2 Department of Pathology, Louisiana State University, Shreveport, LA 71103 USA; 3 Department of Neurosurgery, Rutgers-Robert Wood Johnson Medical School & University Hospital, Rutgers-New Jersey Medical School, New Brunswick, NJ 08901 USA

**Keywords:** FGFR2 amplification, FGFR2-TACC2, FGFR3-TACC3, FGFR3-TLN1, PAI-1, MMP, Leptomeningeal gliomatosis, Transcriptomics, Proteomics

## Abstract

Prognostic molecular subgrouping of glioblastoma is an ongoing effort and the current classification includes IDH-wild-type and IDH-mutant entities, the latter showing significantly better prognosis. We performed a comparative integrated analysis of the FGFR glioblastoma subgroup consisting of 5 cases from a prospective 101-patient-cohort. FGFR alterations included *FGFR2-TACC2* and *FGFR2* amplifications arising in a multifocal IDH-mutant glioblastoma with unexpected 2.5-month patient survival, novel *FGFR3* carboxy-terminal duplication and *FGFR3-TLN1* fusion, and two previously described *FGFR3-TACC3* fusions. The FGFR2 tumors showed additional mutations in *SERPINE1*/PAI-1 and *MMP16*, as part of extensive extracellular matrix remodeling programs. Whole transcriptomic analysis revealed common proliferation but distinct morphogenetic gene expression programs that correlated with tumor histology. The kinase program revealed *EPHA3*, *LTK* and *ALK* receptor tyrosine kinase overexpression in individual FGFR tumors. Paradoxically, all FGFR-fused glioblastomas shared strong PI3K and MAPK pathway suppression effected by *SPRY*, *DUSP* and *AKAP12* inhibitors, whereas the *FGFR2-TACC2* tumor elicited also EGFR suppression by *ERRFI1* upregulation. This integrated analysis outlined the proliferation and morphogenetic expression programs in FGFR glioblastoma, and identified four novel, clinically targetable FGFR2 and FGFR3 alterations that confer aggressive phenotype and trigger canonical pathway feedback inhibition, with important therapeutic implications.

**Supplementary Information:**

The online version contains supplementary material available at 10.1186/s40478-021-01170-1.

## Introduction

Glioblastoma is the most frequent malignant primary brain neoplasm in adults, with an incidence of 3–4 cases per 100,000 population, and 41% survival at 1 year [1]. Approximately 10% of the tumors harbor pathogenic mutations in *IDH1*/*2* genes, which confer a significantly better prognosis, with a median survival of 2.5 years [2]. Many of IDH-mutant glioblastomas result from progression over 4–10 years of lower grade IDH-mutant astrocytomas, hence their name of secondary glioblastoma. IDH-wild-type and IDH-mutant glioblastoma subgroups are recognized as separate molecular entities in the 2016 World Health Organization (WHO) Classification of Tumors of the Central Nervous System (CNS) [3]. The genetic hallmark of IDH-mutant astrocytoma, in general, is the presence of *IDH1*/*2*, *TP53* and *ATRX* mutations, whereas IDH-wild-type glioblastomas show *TERT* promoter mutations, *CDKN2A*/*B* homozygous loss, *TP53* and *PTEN* mutations, as most frequent common alterations [3]. Additional loss of chromosome arm 10q is seen in over 75% of IDH-wild-type glioblastoma and usually spans the *PTEN* locus at 10q23.31, and in 60% of IDH-mutant glioblastoma cases, the most commonly deleted region being 10q25-qter, encompassing the *FGFR2* and *DMBT1* loci [4].

The fibroblast growth factor receptor (FGFR) family of receptor tyrosine kinases (RTKs) comprises four members that share a common structure and activate the extracellular signal-regulated kinase/mitogen-activated protein kinase (ERK/MAPK) and phosphatidylinositol 3′-OH kinase (PI3K)/AKT pathways by constitutively docking FGFR substrate 2 (FRS2) to the juxtamembrane receptor region. They also activate STATs and phospholipase (PL) C-γ by docking them to phosphorylated tyrosine residues, the latter within the carboxyl (C)-terminal tail of the receptor [5]. FGFR driver mutations, amplifications and fusions, as well as FRS2 amplification, are seen in a variety of hematologic and solid cancers [5, 6]. In primary brain cancers, *FGFR1* gain-of-function mutations, kinase domain duplications and fusions, mainly with *TACC1*, *FGFR2* fusions with *CTNNA3* or *KIAA1598*/*SHTN1*, and *FGFR3-TACC3* fusions were reported [7–10]. Of these, the *FGFR3-TACC3* fusions are the FGFR alterations most commonly occurring in IDH-wild-type glioblastoma [11].

We characterized in this study a spectrum of FGFR alterations from a cohort of 101 WHO grade IV diffuse gliomas, including novel FGFR2 and FGFR3 fusions, the former taking place in IDH-mutant glioblastoma, a neoplasm previously not known to harbor oncogenic *FGFR* alterations [9]. By using an integrated proteogenomic approach coupled to temporospatial tumor sampling, we defined both common and unique signaling patterns for the FGFR tumors. In particular, FGFR2 or FGFR3 fusions inducing RTK multimerization activated potent inhibitory feedback loops suppressing the hyperactivation of the canonical MAPK and PI3K pathways, with potential implications for FGFR-targeted therapy.

## Material and methods

### Tumor specimens, autopsy, histology and tumor burden quantification

Surgical resection or biopsy specimens were obtained from patients with glioblastoma, as previously described [12], in accordance to hospital regulations. The autopsy was performed as previously described [13], following the patient’s husband consent for diagnosis and research. A recently described standardized autopsy sampling protocol was applied [14]. FFPE sections from autopsy and surgical specimens were stained with hematoxylin–eosin (H&E). Images were acquired with Nikon Eclipse Ci microscope equipped with Nikon Digital Sight DS-Fi2 camera (Nikon Instruments Inc., Melville, NY), as previously described [15]. The histologic tumor burden was quantified on a 0-to-4 scale, as described [14]. Numerical data were represented graphically by using GraphPad Prism (Version 8.3.0, GraphPad Software, La Jolla, CA).

### Immunohistochemistry (IHC)

IHC was performed on selected sections, as described [12, 15]. The following primary antibodies were used: histone H3-K27M (Millipore/Sigma, Burlington, MA), IDH1-R132H (DIA-H09, Dianova, Hamburg, Germany), p53 (DO-7), vimentin (V9), Ki-67 (30-9) (Roche/Ventana Medical Systems Inc., Tucson, AZ), Olig-2 (387M-15), GFAP (EP672Y) (Ventana/ Cell Marque, Rocklin, CA).

### Transmission electron microscopy

Freshly collected autopsy tumor samples were processed for electron microscopy, as previously described [15, 16]. Digital images were obtained by using AMT Image System (Advanced Microscopy Techniques, Danvers, MA).

### Next generation sequencing (NGS) and copy number variation (CNV)

Nucleic acids were extracted from fresh frozen or FFPE samples, as previously described [12]. FFPE section microdissection was performed for the F48 pituitary section in order to separate normal tissue from neoplastic invasion. NGS was performed in two distinct laboratories for F48 normal and tumor samples. Moreover, F48 autopsy LM_S4_ and DI_S6_ were sequenced from both frozen and FFPE samples, with similar results. The NGS libraries used were: Tempus xT 596-gene or xE whole exome panels [16, 17] for all samples and the customized 295-gene panel [12] for all F48 samples. Variant analysis and interpretation were performed as previously described [12, 16, 17]. CNV analysis was performed as previously described [14]. Gene amplification was called for CN ≥ 8, and loss of heterozygosity (LOH) for alterations with loss of one allele. Tumor mutation burden (TMB) is expressed as single-nucleotide protein-altering mutations per megabase DNA.

### Transcriptomics and statistical analysis

Whole transcriptome RNA sequencing was performed from FFPE-extracted RNA using an exome-capture-based RNA sequencing protocol, as described [18] (Tempus Labs) for all glioblastoma samples with more than 30% tumor on FFPE sections. Briefly, reads were aligned to GRCh38 using STAR (v2.4.0.1), and expression quantitation per gene was computed using FeatureCounts (v1.4.6). Raw read counts were normalized to correct for G+C content and gene length using full-quantile normalization and adjusted for sequencing depth via the size factor method. RNA fusions were detected by quantifying gene-level expression and chimeric transcripts through non-canonical exon-exon junctions mapped using split or discordant read pairs. The expression analysis parameters included threshold setting for total RNA counts ≥ 500 in at least one tumor sample, exclusion of amplified loci, pseudogenes and Y-chromosome genes, and ≥ fivefold expression threshold setting for FGFR tumors relative to DI_S6_ values. Gene classification in 12 non-overlapping functional categories was performed by individual curation. Gene category overexpression median ranking was calculated by using non-parametric, two-tailed Wilcoxon matched-pairs signed rank test. The graphic, statistic and gene classification software included Microsoft Excel (Microsoft Corp., Redmond, WA), GraphPad Prism, and GeneVenn (http://www.bioinformatics.org/gvenn).

### Proteomic analysis

Fresh frozen tissue lysis and Western blotting (WB) were performed as previously described [14]. The primary antibodies are provided in Additional file 1: Table S1, and many were previously tested in autopsy tissue [14]. WBs for each antibody were repeated at least twice, with similar results. The densitometric analysis was performed by scanning the X-ray films with optimal exposures on a ChemiDoc™ Touch imager (Bio-Rad, Hercules, CA). The bands were quantified by using Image Lab 6.0 software (Bio-Rad). Individual protein values were normalized to the corresponding actin or IDH1-R132H values, except for phosphoprotein values that were normalized to the corresponding unphosphorylated protein values. Minus values were manually adjusted as zero. Results were expressed as percent of the highest normalized values.

### Three-dimensional (3D) modeling

The 3D structure of human wild-type PAI-1 engineered to have a long half-life allowing an active conformation [19] (Protein Data Base accession number: 3r4I) was used to model wild-type R210 and mutant H210 residues. Surface models were generated by using PyMol Molecular Graphics System (Version 2.3.0, Schrodinger, LLC), as previously described [13, 20].

## Results

### Clinical overview of glioblastoma FGFR subgroup shows aggressive leptomeningeal (LM) disease linked to FGFR2 alterations

The genomic analysis of a prospective 101 adult patient glioblastoma cohort revealed FGFR alterations in five de novo glioblastomas (Table 1). Four of these were IDH-wild-type glioblastomas with FGFR3 alterations (FGFR3 glioblastoma), occurred in older patients (median age 67 years) and resulted in a 13.6-month median survival. Except for the oldest patient with co-morbidities that had a large, unresectable tumor treated by laser interstitial thermal therapy, the three other patients were amenable to gross total resection followed by radiochemotherapy. Notably, two of the three female patients had prior history of surgically-resected breast cancer (Table 1). Histologically, the FGFR3 glioblastomas showed intense GFAP reactivity and various degrees of “FGFR3-TACC3 glioma recurrent morphological features” [21], in addition to high-grade (HG) features, such as brisk mitotic activity, necrosis and microvascular proliferation (Additional file 1: Fig. S1).

**Table 1:** FGFR glioblastoma patients: clinical-histologic-molecular correlations

Sex/age	Race/smoker	Cancer history	Location	Size (cm)	Res	RT/TMZ/Avastin	Survival (months	Histology	MGMT	TMB	FGFR alteration
F48	W No	No	R Temp	4 × 2	2×	None	2.5	HG-Embryonal HG-LM Rhabdoid LG-DI	neg	2.1	FGFR2↑ FGFR2-TACC2↑
M60	W No	No	L Frontal	4 × 3.4	2×	RT/TMZ/A	20	RMF FGFR3	neg	0.8	FGFR3-TACC3; FRS2↑
F62	W No	BrCa^a^	R Par	3.5 × 3	2×	RT/TMZ	7.3	RMF FGFR3	neg	5.8	FGFR3 CTdup; FRS2↑
F72	W No	BrCa^b^	R Temp	6.8 × 4.5	1×	RT/TMZ/A	23	RMF FGFR3	low	5.8	FGFR3-TACC3
F75	W No	No^b^	R Temp	7.4 × 3.8	LITT	None^c^	2	RMF FGFR3	high	3.2	FGFR3-TLN1

*F* female, *M* male, *W* white, *BrCa* breast cancer, *R* right, *L* left, *Temp* temporal, *Par* parietal, *Res* surgical resection, *LITT* laser interstitial thermal therapy, *RT* radiotherapy, *TMZ* temozolomide, *HG* high grade, *LM* leptomeningeal, *LG* low grade, *DI* diffusely infiltrating astrocytoma, *RMF FGFR3* recurrent morphologic features of FGFR3 glioblastoma, *MGMT* methylguanyl methyl transferase promoter methylation, *TMB* tumor mutation burden, ↑ amplification

^a^Surgically resected breast cancer

^b^History of Hashimoto thyroiditis and arteriosclerosis (myocardial infarction and stroke)

^c^TMZ not tolerated

**Fig. 1:**
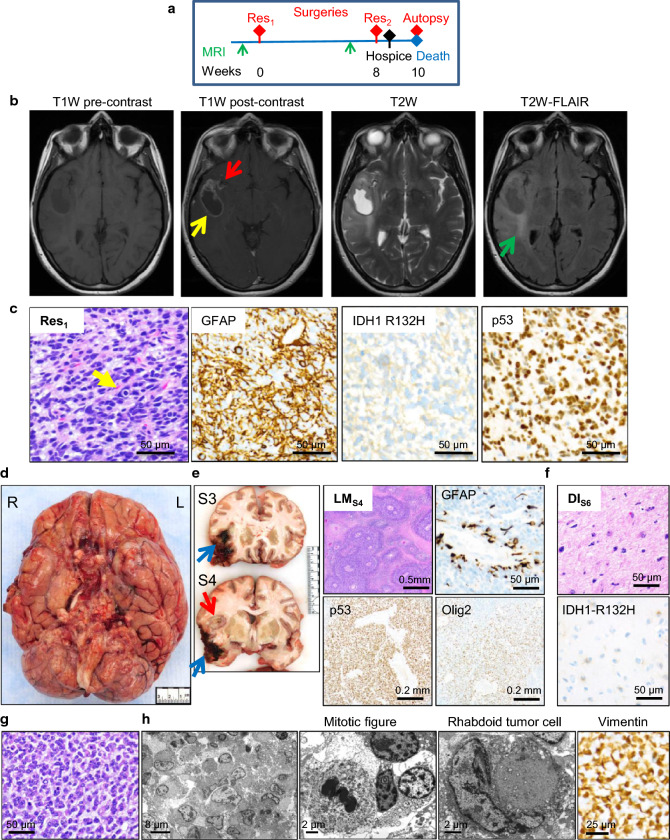
Three radiologically and histologically different neoplasms in F48 IDH-mutant glioblastoma with FGFR2 alterations. **a** Timeline of disease progression. **b** Initial axial MRI shows a rim-enhancing, T2W hyperintense and T2W-FLAIR hypointense mass (blue arrow), Sylvian fissure enhancement (red arrow), and extensive white matter infiltration (green arrow), each corresponding to a different tumor. **c** H&E and IHC with indicated antibodies of the resected rim-enhancing mass (Res_1_). Note HG neuroendocrine/embryonal morphology with hyperchromatic nuclei and frequent mitoses (arrow). IHC with Cam5.2, TTF-1, HMB-45, p40, p63, CD45, estrogen receptor and mammaglobin to exclude a metastatic neoplasm showed negative results (not shown). **d** Autopsy showing the entire brain base covered in a thick, nodular, hyperemic or hemorrhagic LM infiltrate. **e** Gross and microscopic appearance of the HG LM infiltrate. Sections S3 and S4 show the resection site (blue arrows) and the Sylvian fissure LM neoplasm (red arrow). H&E and IHC with indicated antibodies of the Sylvian fissure LM tumor (LM_S4_). **f** H&E and IDH-R132H reactivity of the LG DI neoplasm from S6 white matter (DI_S6_). **g** Close-up H&E of the HG LM component showing rhabdoid cells embedded in myxoid ECM. **h** Ultrastructural evaluation showing true rhabdoid cells with perinuclear whorls of intermediate filaments, and numerous mitotic figures. IHC for vimentin shows strong positivity in the whorls

The tumor from the youngest FGFR patient, F48, had FGFR2 alterations and a very aggressive course, resulting in rapid general status decline that precluded post-resection attempts to radio- and chemotherapy (Table 1, Fig. 1a). F48 was admitted following seizure on the left side of her body with loss of consciousness at work. She also complained of tingling in her 3rd, 4th and 5th digits for 2–3 weeks prior admission, and of occasional headaches and vision blurring for years. Brain magnetic resonance imaging (MRI) showed a right temporal, 4 × 2 cm, rim-enhancing mass, hyperintense on T2-weighted (W) images and hypointense on T2W-FLAIR (Fig. 1b). T2W-FLAIR showed also peri-insular and posterosuperior temporoparietal white matter infiltration (Fig. 1b), and T1W post-contrast images showed contrast enhancement lining the Sylvian fissure, suggestive of LM infiltration (Fig. 1b; Additional file 1: Fig. S2A). Gross total resection (Res_1_) and histopathological examination of the rim-enhancing mass revealed a gelatinous neoplasm with embryonal/HG neuroendocrine morphology, abundant myxoid extracellular matrix (ECM), necrosis, microvascular proliferation and a very high mitotic index, with up to 33 mitotic figures per 1 high power field (Fig. 1c; Additional file 1: Fig S2B). GFAP was positive in a small subset of neoplastic cells, and IDH1-R132H and p53 were diffusely positive in neoplastic cells (Fig. 1c). *MGMT* gene promoter methylation was negative (Table 1). The diagnosis rendered was glioblastoma, IDH-mutant, WHO grade IV, roughly predicting a 2.5-year median survival. An MRI performed one month later was suspicious for meningitis or LM gliomatosis, for which the patient was placed on antibiotics without improvement. A second resection (Res_2_) was performed consisting of re-resection of the initial mass, of a second lower temporal mass, and of multiple dura mater biopsies. Histopathological examination showed LM gliomatosis exhibiting rhabdoid tumor cell morphology, necrosis, and extensive loss of GFAP expression (Additional file 1: Fig. S2B). Following the second surgery, F48 was placed in hospice, and expired 2 weeks later, with a post-surgery survival of 10 weeks. An autopsy was performed within 3 h postmortem. The recorded brain weight was 1215 g, slightly lower than the normal range for age and gender, most likely due to prior resections. The leptomeninges overlaying the base of the brain contained a thick, granular or frankly nodular infiltrate with focal areas of hyperemia, hemorrhage or necrosis, filling the interhemispheric space and encasing all the structures at the base (Fig. 1d). At sectioning, two masses were apparent in the right temporal lobe: a 6 × 4 cm hematoma containing white nodular infiltrate and corresponding to the previous resection sites, and an adjacent 3-cm-diameter necrotic mass in the Sylvian fissure, involving the anterior insula (Fig. 1e; Additional file 1: Fig. S3). Effacement of the grey-white matter junction and induration of the subjacent white matter extended posteriorly in the Heschl’s transverse and upper temporal gyri (Additional file 1: Fig. S3, green arrow). Histopathological examination revealed massive infiltration of the leptomeninges by a HG mucinous neoplasm with extensive geographic necrosis and viable cells arranged around vessels (Fig. 1e). IHC showed diffuse positivity for p53, Olig2 and IDH1-R132H, but GFAP was retained only in a small perivascular subset, similarly to the Res_2_ specimen. In contrast, the diffusely infiltrating (DI) astrocytic neoplasm corresponding to the indurated white-matter areas, displayed a low-grade (LG) appearance, with relatively sparse IDH1-R132H-positive and p53-positive neoplastic cells (Fig. 1f). The HG LM component was composed of small rhabdoid cells with round, mostly uniform nuclei containing salt-and-pepper granular chromatin and sometimes a cherry-red nucleolus (Fig. 1g). Ultrastructural examination confirmed the nuclear features, the numerous mitotic figures and the rhabdoid appearance imparted by perinuclear whorls of intermediate filaments (Fig. 1h). Since both GFAP and cytokeratin immunostains were negative, vimentin was tested, and its strong IHC positivity indicated the nature of the intermediate filaments composing the whorls. From the LM space, the rhabdoid neoplasm re-invaded the brain through Virchow–Robin perivascular spaces, with breach of the pia mater, and either radial diffusion into the parenchyma with single cell invasion on a short distance or “en bloc” penetration of the parenchyma (Additional file 1: Fig. S2C). Many apparently uninvolved structures grossly were microscopically invaded by the LM neoplasm (Additional file 1: Fig. S2D). Semiquantitative evaluation of the tumor burden showed extensive, and in most part, massive infiltration of the brain structures by the LM component (Additional file 1: Fig. S2E). The LG DI component showed only a low tumor burden within the cerebral white matter and white matter tracts.

### Novel FGFR alterations in glioblastoma

**Fig. 2:**
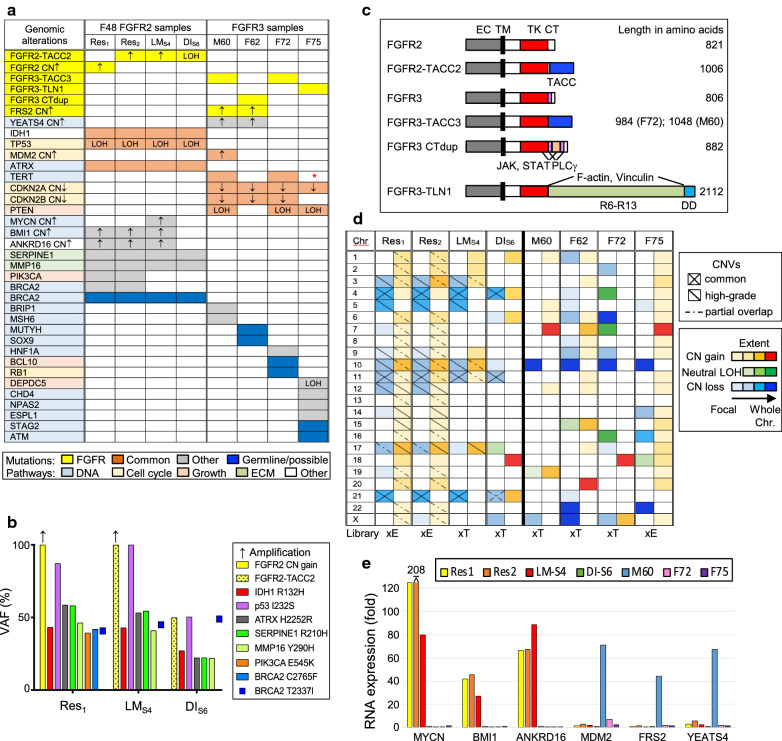
FGFR glioblastoma genomic analysis. **a** NGS analysis showing color-coded alterations. White boxes indicate lack of mutations. CNV: ↑, CN ≥ 8; ↓, CN = 0. Red * indicates lack of TERT promoter inclusion in the whole exome NGS. **b** Variant allele fraction (VAF) of the indicated mutations from F48 samples. DI_S6_ shows proportionally lower values due to a higher normal component within the sample. **c** Schematic diagram of the FGFR2 and FGFR3 fusions and duplication. Protein amino acids numbers are shown. Domains are indicated: EC, extracellular; TM, transmembrane; TK, tyrosine kinase ; CT, C-tail; TACC, transforming acidic coiled-coil; R6-R13, talin rod helical bundles 6–13; DD, dimerization domain. Binding sites for the indicated proteins are shown . For FGFR2-TACC2, the hybrid protein joined the last residue, E767, of FGFR2 coding exon 16 (Isoform 1, FGFR2IIIc, NM_000141), to the first residue, R2710, of TACC2 exon 17. **d** CNV heatmap showing the extent of chromosomal involvement by CNVs. The library used for the CNV analysis is shown at the bottom. **e** Transcriptomics expression analysis showing the relative mRNA expression of genes with amplification

NGS was performed on six F48 samples, including normal tissue, and on the four FGFR3 glioblastoma surgical specimens. F48 showed the common somatic mutation signature of IDH-mutant astrocytomas comprising the *IDH*/*TP53*/*ATRX* triad [3]: *IDH1* p.R132H, *TP53* p.I232S with LOH, and *ATRX* p.H2252R, which represents a novel *ATRX* mutation (Fig. 2a, b; Additional file 1: Table S2). Interestingly, additional common somatic mutations occurred in the *SERPINE1* p.R210H, a hotspot in colorectal cancer [22], and *MMP16* p.Y290H ECM remodelers. Pathogenic somatic mutations in *PIK3CA* p.E545K and *BRCA2* p.C2765F were present only in the F48 resections. F48 also showed a germline *BRCA2* T2337I variant of unknown significance (VUS) (ClinVar, multiple submitters). *FGFR2* or *FGFR2-TACC2* fusion amplifications, resulting in massive mRNA overexpression, were present in all F48 HG specimens, and correlated with the embryonal or rhabdoid morphology, respectively (Fig. 2a–c; Additional file 1: Fig. S4). Importantly, DI_S6_ also contained the *FGFR2-TACC2* fusion with LOH. The fusion RTK contained all FGFR2 amino (N)-terminal domains, including the entire tyrosine kinase (TK) domain, and swapped the FGFR2 54-residue C-terminus for the intact 239-residue TACC domain of TACC2 (Fig. 2c; Additional file 1: Table S3).

The FGFR3 glioblastoma cases exhibited the most common mutations found in IDH-wild-type glioblastoma [3], with *CDKN2A* homozygous loss in all cases, followed by *PTEN* mutations with LOH and *CDKN2B* homozygous loss, in three cases, and *TERT* c.124C>T promoter mutation in two cases (Fig. 2a, Additional file 1: Table S2). *FRS2* and neighboring oncogene *YEATS4* amplification were present in two cases. Putative germline pathogenic mutations, as assessed by their homozygous variant allele frequency and from previous reports, were present in the female patients with breast cancer history, such as *MUTYH* p.G396D (ClinVar, 33 submitters) and *RB1* p.R451H (ClinVar, 2 submitters) in F62 and F72, respectively. M60 presented additional pathogenic alterations of the DNA damage response (DDR) pathway, and F75 showed pathogenic mutations in multiple tumor suppressor genes, such as *DEPDC5* p.W905*, an mTOR inhibitor [23], and several nuclear DNA effectors. The FGFR3 alterations consisted of two distinct, previously reported *FGFR3-TACC3* fusions [10], a novel *FGFR3* C-tail insertion with duplication (FGFR3 CTdup), and a novel FGFR3-TLN1 fusion (Fig. 2a, c; Additional file 1: Table S3). The FGFR3 CTdup contained an extra 76-residue insertion that added a novel Proline-rich region with a putative PXXPXP JAK-binding motif, as well as non-canonical SH3-interacting motifs [24, 25], and duplicated the first half of the C-tail, including the PLCγ interaction Y-motif (Additional file 1: Fig. S5). The *FGFR3-TLN1* fusion joining the FGFR3 TK domain to talin C-terminal half resulted from chromosomal translocation. Talin is the cytoskeletal protein that couples F-actin to integrins at the focal adhesions, and the moiety involved in the FGFR3-TLN1 fusion included the F-actin-binding region and the C-terminal dimerization domain [26]. This talin moiety is predicted to constitutively activate the fused FGFR3 via dimerization, similarly to other FGFR fusions [27], and possibly mislocalize it via interaction with the actin cytoskeleton rather than with microtubules as the TACC domain [10, 28].

The CNV analysis showed only two common CN alterations for all F48 samples: chromosome 4q and 11p heterozygous losses (Fig. 2d; Additional file 1: Table S4). The three F48 HG samples contained one common 17q21.33-qter gain and several CN losses: 3p22.2-p21.1, 5q, 10q23.31-qter except for the *WDR11-FGFR2-TACC2* locus, and 21, which partially overlapped with the 21q22.11-qter loss from DI_S6_. Unique CNVs were present in all the samples but they were numerous for the LG DI_S6_, suggesting divergent evolution. All FGFR3 glioblastoma cases showed chromosome 10 deletion, and the overlap with the F48 10q23.31-qter deletion represented the only common CNV alteration between the FGFR3 and FGFR2 glioblastomas. Complete or partial chromosome 7 gain and chromosome 22 loss, which are other common chromosomal aberrations in IDH-wild-type glioblastoma [3], were present in three and two cases, respectively, whereas F72 had neither, showing unexpected chromosome 7 neutral LOH. Focal CNVs were better documented in the surgical samples from F48 and F75 for which whole exome NGS was performed (Additional file 1: Table S4). For the genes with CN gain ≥ 5, the RNA expression was analyzed and showed that only a minority resulted in overexpression (Fig. 2e). Only *MYCN*, *ANKRD16*, involved in translation fidelity [29], *BMI1,* encoding a major component of the Polycomb repressive complex 1 involved in transcriptional repression and DDR [30], and *FGFR2* were highly overexpressed in all F48 HG tumors. M60 tumor exhibited two chromosome 12 loci resulting in high gene overexpression, including of *MDM2*, *YEATS4* and *FRS2* (Additional file 1: Table S4).

### Gene expression programs in FGFR glioblastoma correlate with morphology

**Fig. 3:**
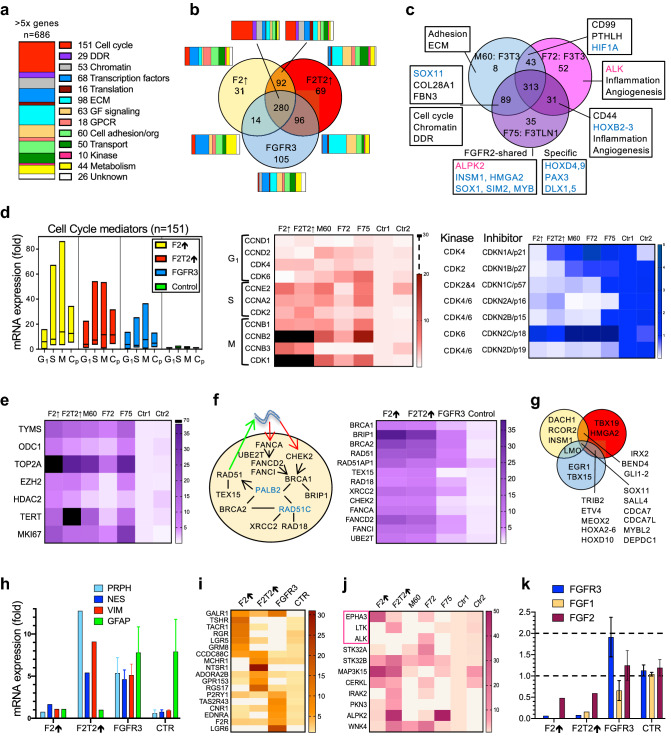
Gene expression programs in FGFR glioblastoma. **a** Vertical slice chart representing the ensemble of ≥ fivefold overexpressed genes from FGFR tumors classified in functional categories. The number of genes in each functional category is shown. **b** Venn diagram depicting the sharing of the same gene ensemble as in **a** between FGFR2 tumors (Res_1_/F2↑ and LM_S4_/F2T2↑) and the FGFR3 subgroup. Horizontal-slice charts of functional gene categories are shown for each of the 7 Venn diagram subsets. **c** Venn diagram of the ≥ fivefold overexpressed genes for the three FGFR3 cases with available RNA expression data: M60 and F72, sharing the *FGFR3-TACC3* (F3T3) fusion, and F75 showing *FGFR3-TLN1* (F3TLN1) fusion. The overexpressed genes from the two M60 amplified loci on chromosome 12 were not included in the expression analysis except for *PTHLH*, which encodes the hormone responsible for the hypercalcemia of malignancy. The functional gene categories or examples of genes associated with different Venn diagram subsets are shown boxed, with kinases, in magenta, and transcription factors, in blue. **d** Color-coded floating-bar graph for G1-, S- and M-phase and checkpoint (Cp) cell cycle genes. Fold-overexpression heatmaps are shown for cyclins/CDKs, and for CDK inhibitors. The corresponding CDKs inhibited by each inhibitor are listed. **e** Fold-overexpression heatmap of selected proliferation enzymatic therapeutic targets and *MKI67*. **f** Diagram and fold-overexpression heatmap of the BRCA1/2 DDR complex. Red and green arrows show recruitment to and repair of DNA damage, respectively; genes in blue are not overexpressed. **g** Venn diagram with representative examples of overexpressed transcription factors. **h** Mean ± SEM mRNA expression graph for intermediate filament genes. **i**, **j** Fold-overexpression heatmaps for the GPCR and Kinase functional categories. RTKs are boxed. **k** Mean ± SEM graph showing *FGFR3* and *FGF1-2* mRNA fold expression levels. CNV analysis showed normal 2-copy complement for *FGFR3,* and loss of one copy for *FGF2* for all F48 tumors*. FGF1* showed loss of one copy in F2↑ and F2T2↑, and normal complement in LG DI_S6_ control

Comparative gene expression analysis was performed for samples with available whole transcriptomics data. The unsupervised analysis of the FGFR glioblastoma gene expression uncovered a total of 686 genes with ≥ fivefold overexpression compared to DI_S6_ values, further classified in 12 functional categories (Fig. 3a). The large majority of the overexpressed genes, composed mainly of cell cycle genes, was shared between FGFR3 and FGFR2 tumors (Fig. 3b). Pairwise, Res_1_/F2↑–LM_S4_/F2T2↑, and F2T2↑–FGFR3 showed significant overlap of cell cycle and ECM/growth factor genes, respectively. F2↑ showed specific predominance of G protein-coupled receptors (GPCRs). Within the FGFR3 subgroup, gene overexpression unexpectedly showed more overlap between M60 and F75 tumors that harbored *FGFR3* fusions with different partners than between M60 and F72 (Fig. 3c). The discrepancy was caused by a set of chromatin and cell cycle genes that were not significantly upregulated in the F72 tumor, suggesting less aggressive proliferation. The F72 and F75 tumors shared genes mediating inflammation and angiogenesis, whereas *ALK* and *ALPK2* kinases were specifically expressed in each tumor, respectively.

To understand the biology of the tumors, each functional gene category was compared between the morphologically different F2↑, F2T2↑ and FGFR3 tumors. The ranking of overexpression values in tumor proliferation categories, such as cell cycle, DDR and chromatin, showed F2↑, followed by F2T2↑, as the most proliferative tumors (Additional file 1: Fig. S6). In-depth analysis of cell cycle genes showed high upregulation of genes governing S and M phases, as well as of cell cycle checkpoint genes in the FGFR2 subgroup (Fig. 3d). The examination of cyclin, CDK, and CDK inhibitor expression levels showed distinct upregulation of G1-phase CDK4 in FGFR2 tumors, and cyclin D2 and CDK6 in FGFR3 tumors. *CDKN2A* and *CDKN2B* expression correlated perfectly with their homozygous CN loss in the FGFR3 tumors (see Fig. 2a), and showed also decrease in the FGFR2 tumors, in the absence of CNV. Among druggable cell cycle and chromatin genes, *TOP2A*, *TYMS*, *TERT*, *EZH2*, *ODC* and *HDAC2* showed high overexpression levels in FGFR2 tumors (Fig. 3e). Within the FGFR3 subgroup, M60 and F75 showed lower but comparable cell-cycle-related overexpression values to FGFR2 tumors, while F72 had consistently low values, including for *TERT* that harbored the c.124C>T promoter mutation. Many of the DDR genes from the BRCA1/2 complex that is recruited to RAD51 DNA-damage foci [31] showed massive overexpression in the FGFR2 tumors (Fig. 3f).

Overexpression ranking in functional categories reflecting tissue specification, environment and architecture, placed generally F2T2↑ and FGFR3 tumors close together, with more developed programs than F2↑ (Additional file 1: Fig. S6). The transcription factor category was rather heterogenous, consisting of common or more specific factors (Fig. 3g). Of these, the INSM1-RCOR2 repressor complex specifying neuroendocrine development [32], was overexpressed in the F2↑ and F75 tumors. Interestingly, another transcription repressor *DACH1*, known to mediate a negative FGF signaling feedback loop through *FGF2* repression [33, 34], showed ≥ 15-fold specific overexpression in the F2↑ tumor.

The closely related ECM and growth factors expression programs showed the most complex activity in the F2T2↑ tumor, followed by FGFR3 tumors (Additional file 1: Fig. S7). Although many of the signaling pathways shown were significantly upregulated in F2T2↑, most were shared with the other tumors, noteworthy being the ALK and insulin receptor RTK pathways. For the cell adhesion/organization program, a common core of actin cytoskeleton genes was upregulated in all tumors (Additional file 1: Fig. S7). Important differences distinguished FGFR2 from FGFR3 tumors. Selective upregulation of a different subset of glycosylation and sulfation ECM remodeling enzymes was present in FGFR2 tumors, most likely explaining their mucinous background. Cytokines and their receptors, pro-angiogenetic factors, and inflammatory mediators were predominantly upregulated in the FGFR3 tumors. Importantly, very high *VEGFA* expression values were noted in F2T2↑ and FGFR3 tumors. *FN1* encoding fibronectin, the ligand for integrins, and the integrin cell adhesion program were robustly increased in FGFR3 tumors, suggesting an essential difference in a major ECM adhesion signaling pathway. Two signaling pathways were predominantly upregulated in F2T2↑: Wnt, and Sonic Hedgehog (SHH) and related ciliogenesis pathway. The F2↑ tumor showed a complementary pattern of low expression for all intermediate filament genes, and high overexpression for most genes involved in cell–cell junctions. The intermediate filaments *NES*, *VIM* and *PRPH* were overexpressed, whereas *GFAP* was underexpressed in FGFR2 tumors, consistent with the IHC results (Fig. 3h).

Signaling through GPCR and kinases showed relatively specific profiles in FGFR tumors. The GPCRs showed complementary overexpression profiles for F2↑, F2T2↑ and FGFR3 tumors (Fig. 3i). In particular, F2↑ overexpressed a neuroendocrine receptor program, including *TSHR*, *TACR1*, *RGR*, *LGR5* and *GRM8*. Eleven kinases other that *FGFR2* showed ≥ fivefold overexpression in the FGFR tumors, the commonly overexpressed being *STK32B* (Fig. 3j). *EPHA3*, *LTK*, an *ALK* homologue, and *ALK* were the only RTKs of the category, and showed specific overexpression in F2↑, F2T2↑, and F72, respectively. Among the FGFR family, *FGFR3* expression was mildly increased in FGFR3 tumors, but 15-fold underexpressed in the FGFR2 tumors, (Fig. 3k). In addition, *FGF1* and *FGF2* expression levels showed 111-fold and 2.5-fold decrease, respectively, in the FGFR2 tumors, suggesting an FGFR2-mediated negative feedback.

### Lack of canonical pathway activation in FGFR-fused glioblastoma relies on activation of inhibitory feedback loops

**Fig. 4:**
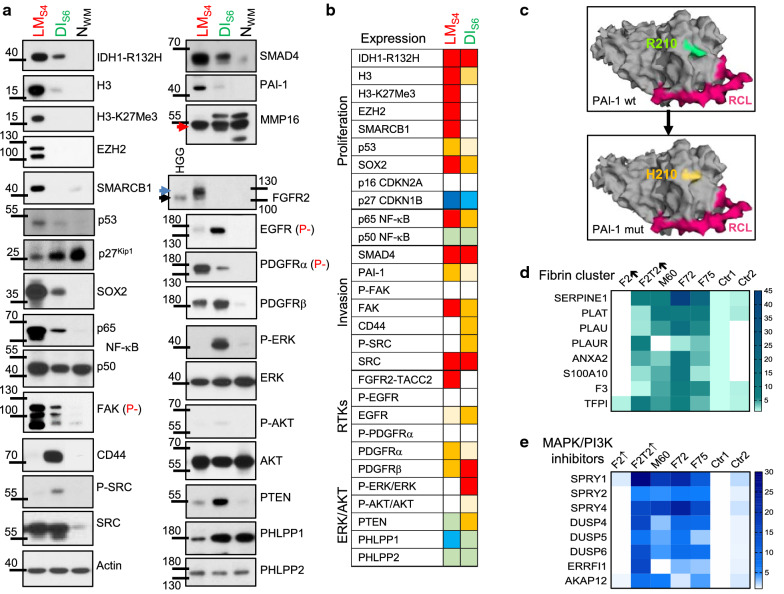
Signaling pathway activation and feedback inhibition. **a** WB with indicated antibodies of total protein lysates (50 µg protein) from LM_S4_, DI_S6_, and normal white matter control (N_WM_) fresh frozen autopsy samples. Molecular weight markers are indicated. For FGFR2, an autopsy HG glioma control with FGFR2 overexpression run on the same blot is shown for comparison. The blots performed for phosphorylation detection yielding negative results are not shown but the result is indicated with (P-) in red, following the corresponding protein. **b** Heatmap of semiquantitative WB, as shown quantified and normalized in Additional file 1: Fig. S8. White, green and blue shades indicate lack of expression, similar expression to control, and decreased expression, respectively. Incremental red or blue shades indicate incrementally increased or decreased expression, respectively. The maximum of a protein expression increase is shown as red or orange based on comparison with other glioblastoma samples. **c** Structural alterations induced by SERPINE1/PAI-1 R210H mutation. Surface 3D representations of active PAI-1 with wild-type R210 residue in green and mutant H210 residue in orange. The reactive center loop (RCL) of PAI-1, which is the binding site of uPA, is shown in magenta. Note significant surface change induced by the side chain of H210 mutant in the vicinity of RCL. Protein Data Base of the crystal structure accession number: 3r4I. **d**, **e** Fold-expression heatmaps of the ECM remodeling fibrin cluster and MAPK/PI3K inhibitor genes. CNs for RTK inhibitor genes were identical in all F48 tumors, except for *SPRY4* and *DUSP5* that showed normal 2-copy complement in DI_S6_ and loss of one copy in the HG F2↑ and F2T2↑ tumors

To evaluate the activation of canonical growth signaling pathways, WB proteomic analysis was performed for F48 LM_S4_ and DI_S6_ (Fig. 4a, b; Additional file 1: Fig. S8). Consistent with the IHC and NGS results, both LG and HG F48 tumor components expressed IDH1-R132H. Epigenetic changes consisted of upregulation of histone H3 in both components, although less prominent in DI_S6_. K27 trimethylation was detected only in LM_S4_ and correlated with overexpression of EZH2. SMARCB1, the core component of the SWI/SNF chromatin remodeling complex that is mutated in rhabdoid neoplasms [35], was overexpressed only in LM_S4_, suggesting that the rhabdoid morphology of the LM component is not due to *SMARCB1* mutation. The p53 levels were mildly increased, due to mutant protein stabilization. The CDK inhibitors p16 INK4A/*CDKN2A* and p27^Kip1^/*CDKN1B* were either not expressed or showed significant downregulation compared to control, respectively, correlating with the mRNA expression results. The stem cell factor SOX2 that is required for maintenance of undifferentiated neural stem cells [36] showed high expression in LM_S4_, but also moderate levels in DI_S6_. The analysis of NF-κB pathway downstream of cytokine signaling showed strong or moderate upregulation of p65 RelA in LM_S4_ and DI_S6_, respectively, whereas p50 levels were comparable to normal white matter control. Focal adhesion kinase (FAK), a non-RTK implicated in cell migration downstream of integrin signaling [37], was strongly upregulated in both components, but more prominently in LM_S4_, in the absence of phosphorylation or significant RNA expression increase, implicating protein stability as an important mechanism of protein expression levels. The hyaluronic acid receptor CD44 that has been shown to be upregulated in glioblastoma compared to LG astrocytoma [38] was unexpectedly overexpressed in DI_S6_. CD44 interacts with c-SRC kinase [39], and CD44 upregulation correlated with c-SRC activation, although c-SRC total expression levels were upregulated in both components.

SMAD4, the common transducer of TGF-β and bone morphogenic protein signaling, and its transcriptional target plasminogen activator inhibitor 1 (PAI-1) encoded by *SERPINE1* gene [40] showed upregulation in both components, more elevated in LM_S4_ for PAI-1. PAI-1 R210H somatic mutation maps to a short β-strand in the Serpin domain exposed to the surface, relatively close to the reactive center loop that is the site of interaction with urokinase plasminogen activator (uPA) [41]. The missense mutation H210 induces a relatively significant surface change that may interfere with uPA binding (Fig. 4c). In addition to the mutation, PAI-1/*SERPINE1*, but also its enzymatic targets uPA/*PLAU*, tPA/*PLAT*, and their receptors uPAR/*PLAUR*, annexin A2/*ANXA2* and p11/*S100A10* forming the ECM remodeling fibrin cluster were overexpressed to various levels in LM_S4_/F2T2↑, but also in the FGFR3 tumors (Fig. 4d). The membrane-type metalloproteinase MMP16 exhibiting a Y290H mutation mapping to the C-end of the peptidase domain showed similar levels in all samples, consistent with its reported expression in glioma and normal brain [42]. However, it demonstrated only the active processed form [43] in LM_S4_ (Fig. 4a, red arrow). This active form cleaves and activates MMP2, one of the gelatinases that have been consistently involved in cancer metastasis [44]. As the fibrin cluster, the peptidase cluster contained many differentially overexpressed MMPs, including MMP2, in the LM_S4_/F2T2↑ tumor but also in FGFR3 tumors (Additional file 1: Fig. S7).

FGFR2 expression was massively and exclusively upregulated in LM_S4_, and even high exposure failed to show expression in DI_S6_, consistent with the genomic and transcriptomic results. The main massively overexpressed band in LM_S4_ migrated approximately 20 kDa upper than the overexpressed wild-type FGFR2 from other HG glioma samples [14], most consistent with the molecular weight of FGFR2-TACC2 (Fig. 4a, blue arrow). EGFR, which is known to be upregulated in infiltrating gliomas [45], showed very low expression in LM_S4_, unlike other tested glioblastomas [38], and moderate overexpression in DI_S6_, without phosphorylation. PDGFRs exhibited complementary expression, with PDGFRα showing higher expression in LM_S4_, and PDGFRβ, in DI_S6_. Unexpectedly, ERK, and to a much lesser extent AKT, were activated in the LG DI_S6_ but not in LM_S4_, a finding uncommon for HG gliomas that usually show both ERK and AKT pathway activation [14, 46]. The PI3K/AKT pathways inhibitors PTEN and PHLPP1-2 [46] were expressed in both components, with PTEN and PHLPP1 showing decreased levels in LM_S4_, contrasting with the lack of AKT activation.

FGFRs signal through FRS2 and GRB2 adaptors to activate the canonical ERK/MAPK and PI3K pathways but may also elicit negative feedback loops [5]. Whole transcriptomics showed that 8 of the 686 overexpressed genes are RTK signaling inhibitors (Fig. 4e). The FGFR signaling inhibitors Sprouty (SPRY) 1, 2 and 4, which inhibit the activation of both PI3K and ERK, and Dual-specificity phosphatases (DUSP) 4, 5 and 6 that directly dephosphorylate ERK1/2 showed highest levels in the LM_S4_/F2T2↑ and FGFR3 tumors. RALT/*ERRFI1*, another RTK inhibitor transcriptionally upregulated by ERK activation, is an EGFR catalytic inhibitor and degradation inducer [47]. *ERRFI1* expression was specifically and strongly (16-fold) upregulated in the LM_S4_/F2T2↑ tumor, explaining the downregulation of EGFR in this tumor in the absence of significant mRNA expression change (Fig. 4a). AKAP12, an A-kinase anchoring protein and ERK inhibitor [48] showed mRNA overexpression in LM_S4_/F2T2↑ and to a lesser extent in FGFR3 tumors (Fig. 4e). Taken together, these data suggest that the lack of the canonical pathway activation observed in the LM_S4_/F2T2↑ tumor is most likely due to a potent negative feedback aligning multiple RTK/MAPK/PI3K inhibitors.

## Discussion

The WHO molecular subgrouping into IDH-wild-type and IDH-mutant glioblastoma emphasizes a significantly longer survival for the IDH-mutant subgroup, due to a slower tumor growth rate, and reflected in a more insidious onset [4]. Although most IDH-mutant cases conform to this biological behavior, we describe here the first case of de novo IDH-mutant glioblastoma with *FGFR2* alterations that induced fulminant progression with LM spread, resembling the most aggressive cases of IDH-wild-type glioblastoma. To better understand the role of FGFR in glioblastoma, we performed a comparative genomic, transcriptomic and proteomic analysis for the FGFR tumors from a prospective 101-adult-patient cohort with WHO grade IV diffuse gliomas. We found that the multifocal FGFR2 tumors exhibited relatively unique morphogenetic programs, whereas the four FGFR3 IDH-wild-type tumors showed relative histologic and signaling homogeneity. Despite apparent FGFR3 subgroup homogeneity, patient survival was significantly better for the two cases with *FGFR3-TACC3* fusions than for the cases with *FGFR3* C-terminal duplication or *FGFR3-TLN1* fusion that shared a more posterior location and chromosome 22 loss.

**Fig. 5:**
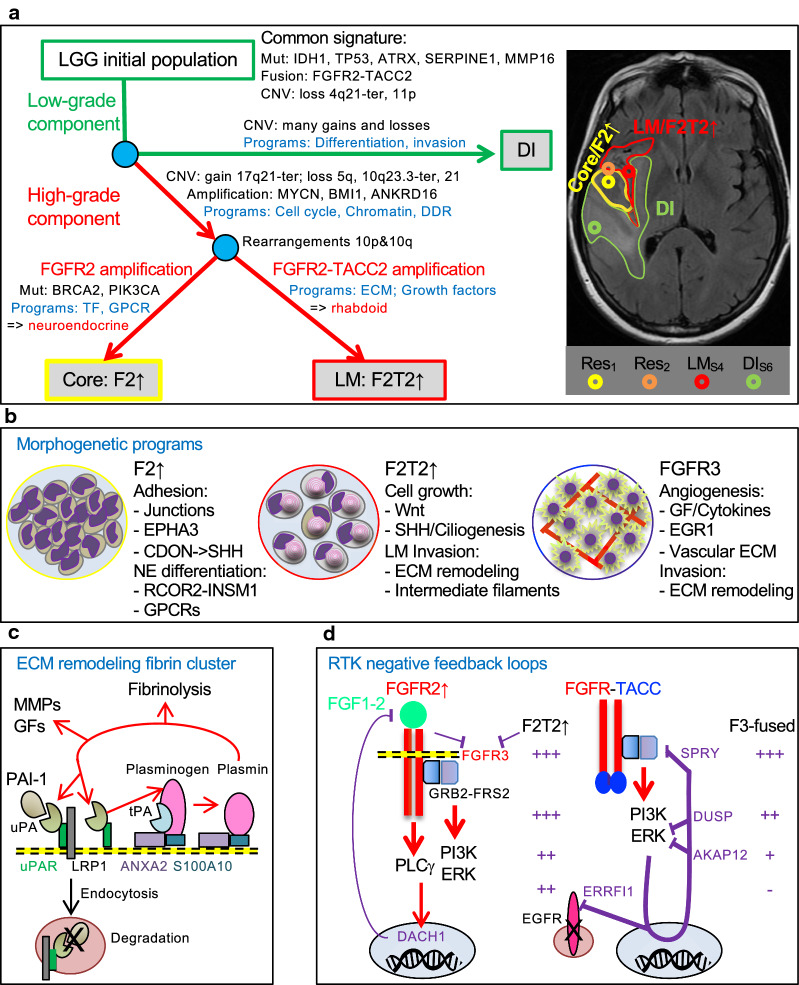
Oncogenetic programs in FGFR glioblastoma. **a** Nodal evolution of the LG FGFR2-TACC2 IDH-mutant astrocytoma (green tracing) into HG core F2↑ and LM F2T2↑ components (red tracing). Nodes are indicated with blue circles. Res1/Res2, first/second resections. The axial T2W-FLAIR MRI shows the outlined tumor components and the approximate 2D projection of the samples (circles). **b** Morphogenetic programs in FGFR glioblastoma with cartoon representation of specific morphologic features: HG neuroendocrine (NE)/embryonal, showing nuclear molding and high nuclear-cytoplasmic ratio; rhabdoid, showing eccentric nuclei and paranuclear vimentin whorls; FGFR3-TACC3 recurrent morphological features, showing cells with monomorphous nuclei aligned along chicken-wire capillaries. **c** Cartoon representation of the ECM remodeling fibrin cluster strongly upregulated in F2T2↑ and FGFR3 tumors. Red arrows indicate proteolytic cleavage. The end-product of the pathway, plasmin, activated by both uPA and tPA and membrane-bound via the heterotetrameric receptor complex formed by annexin A2/*ANXA2* and p11/*S100A10*, activates uPA, growth factors (GFs), MMPs and other ECM components beside degrading fibrin. **d** Negative feedback loops regulating canonical pathway signaling are represented with thick or thin purple lines when the mechanism is known or only putative, respectively. FGFs bind to transmembrane amplified FGFR2 (F2↑ tumor) that activates (red arrows) canonic PI3K and ERK pathways but also PLCγ through a C-terminal Y-motif. FGF1-2 feedback inhibition may stem from DACH1, a transcriptional repressor downstream of FGFR signaling. In FGFR-fused tumors with moieties promoting mislocalization and constitutive FGFR dimerization and activation, only the PI3K and ERK canonic pathways are activated. Strong ERK activation leads to transcription of multiple pathway inhibitors, with corresponding expression levels shown for F2T2↑ and FGFR3-fused tumors

By performing autopsy and corroborating findings with the patient’s clinical history, we revealed that an undiagnosed pauci-symptomatic LG astrocytoma slowly progressed in F48 before the HG neoplastic populations emerged (Fig. 5a). For secondary IDH-mutant glioblastoma evolving from LG astrocytoma, Ohgaki et al. proposed the loss of 10q25-qter region as contributing to the malignant transformation [49]. In F48, a paradoxical *FGFR2* locus amplification on 10q26.13 with CN loss of 10q25-qter resulted in massive *FGFR2* overexpression. Although novel to brain tumors, *FGFR2* amplification has been reported in gastric cancer where it imparts poor prognosis [50, 51], triple-negative breast cancer, hormone-resistant prostate cancer and in an isolated case of colorectal carcinoma [52–55]. In contrast, *FGFR2-TACC2* fusion without amplification has only been reported in a case of apocrine breast cancer [56], despite the fact that *FGFR2* fusions with various partners, including *FGFR2-TACC3*, are relatively common in intrahepatic cholangiocarcinoma [27]. Therefore, the F48 LM tumor is the first case showing amplification of the rare *FGFR2-TACC2* fusion. Noteworthy, both fused and unfused amplified *FGFR2* products are targetable molecular alterations, and current clinical protocols in gastric cancer and cholangiocarcinoma address with some success both forms [6, 27]. It is not clear what triggered the amplification of the *FGFR2* locus, but it appeared to coincide with the 10q23.3-qter loss, as well as with a turning point where *MYCN* and *BMI1* amplifications with overexpression, distinguished the LG and HG populations (Fig. 5a). Of these, *MYCN* amplification has been previously reported and shown to correlate with shorter survival in IDH-mutant glioblastoma [57, 58], and may have contributed to the aggressive course of this case, as well. Interestingly, a second turning point involved the separate evolution of the two main HG populations, in which F2↑ acquired higher genomic instability, especially of chromosome 10, but also pathogenic mutations in *PIK3CA* and *BRCA2*. The *BRCA2* somatic mutation may have contributed to genome instability together with the germline *BRCA2* VUS, malfunctioning within an upregulated BRCA1/2 complex shown here to be a DDR housekeeping mechanism in all highly-proliferating tumors.

The tumor biological behavior was assessed by a well-structured gene expression functional classification addressing proliferation and morphogenesis. The proliferation programs ranked FGFR2 and most FGFR3 tumors as highly proliferative, and several targetable molecules, such as TOP2A, TYMS, EZH2 and ODC1 showed very high levels. Interestingly, the FGFR2 tumors with *ATRX* mutation, had high *TERT* overexpression, suggesting TERT-dependent telomere extension as common denominator in all FGFR tumors. The morphogenetic programs showed upregulation in F2↑ of RCOR2-INSM1 transcription factor complex specifying neuroendocrine development [32], neuroendocrine GPCRs, *EPHA3* RTK and CDON cell adhesion and SHH receptor [59], explaining the HG neuroendocrine differentiation with nuclear molding (Fig. 5b). The LM F2T2↑ rhabdoid tumor showed a massive upregulation of a unique growth factor program with selectively activated Wnt and SHH/ciliogenesis pathways, and an intermediate filament expression switch, with GFAP loss, and peripherin and vimentin massive overexpression. The recurrent morphological features of FGFR3-fused tumors may have been contributed by a robust angiogenesis program orchestrated by EGR1, downstream of VEGFA secretion [60], cytokine signaling, and a developed vascular ECM component. A relatively common program, shared by FGFR-fused tumors, specified a network of ECM remodeling peptidases, their activators, inhibitors and receptors (Fig. 5c). Of these, PAI-1/*SERPINE1* and *MMP16* showed pathogenic mutations in the FGFR2 tumors, a novel finding in glioblastoma. *PAI-1* R210H mutation might interfere with uPA or tPA binding, or modulate the endocytosis and degradation of the PAI-1-uPA complex [61], whereas the MMP16 Y290H mutation induced processing towards the protein active form that activates MMP2 [62].

Proteomic pathway analysis showed paradoxical findings in FGFR2-TACC2 HG and LG components. Whereas the LG DI tumor showed common glioma changes, such as EGFR upregulation [38, 45], and ERK/MAPK and CD44/c-SRC pathway activation, the latter involved in glioma cell invasion [63, 64], the HG LM component lacked these changes. Similar data, showing lack of canonical MAPK and PI3K pathway activation, has been obtained in astrocytes transfected with FGFR3-TACC3 but not with kinase-dead FGFR3-TACC3 constructs [10]. The findings are even more surprising considering that the FGFR-TACC molecules are constitutively activated by di- or multimerization through the TACC domain [27, 28], and that FGFR signaling results in activation of both canonical pathways via FRS2 and GRB2 adaptor proteins [5]. Moreover, *FRS2* amplification in half of the FGFR3 tumors and the *FGFR2-TACC2* amplification from the HG LM component would be expected to fuel the FGFR downstream signaling. The explanation of these unexpected findings is the simultaneous upregulation of a potent FGFR negative feedback composed of Sprouty family members that block the activation of both canonical pathways, and of DUSP4/5/6 that dephosphorylate ERK1/2 [5, 65] (Fig. 5d). *AKAP12*, another ERK1/2 inhibitor [48], was found preferentially upregulated in FGFR tumors, whereas RALT/*ERRFI1*, an EGFR inhibitor and degradation promoter [47], was strongly overexpressed only in the F2T2↑ tumor, explaining its paradoxical EGFR expression loss. Noteworthy, *SPRY*, *DUSP* and *ERRFI1* are controlled transcriptionally by ERK1/2 signaling [5, 47, 65], and showed low expression levels in the LG DI compartment that lacked FGFR2-TACC2 overexpression, suggesting a dynamic, dose-dependent relationship between RTK signaling and feedback inhibition. Surprisingly, these inhibitors also exhibited low levels in the F2↑ tumor, and we hypothesize that their lack is linked to the presence of degradation signals in the FGFR C-terminus [5, 27] and absence of constitutive dimerization provided by the TACC domain [10, 66], perhaps allowing overexpressed FGFR2 a more physiological signaling controlled by strong negative feedbacks, possibly mediated by DACH1 [33, 34] (Fig. 5d).

In conclusion, we discovered novel targetable mutations in the FGFR glioblastoma subgroup, including FGFR2 alterations occurring during the evolution of a multifocal and unusually aggressive IDH-mutant astrocytoma. Comparative expression analysis within the FGFR glioblastoma subgroup uncovered common proliferation and unique morphogenetic programs, including the upregulation of targetable non-FGFR RTKs. In particular, tumors exhibiting FGFR fusions upregulated invasion-related ECM remodeling pathways to much higher extent than previously appreciated. Importantly, this analysis revealed MAPK/PI3K pathway inhibitory loops with strongest upregulation in FGFR-fused tumors, pinpointing potential resistance to RTK therapy. This study also encourages efforts towards more extensive characterization of aggressive glioblastoma variants, as some may harbor targetable alterations with existing therapeutic protocols.

## Supplementary Information

**Additional file 1 Figure S1**. FGFR3 glioblastomas: MRI and histology.** Figure S2**. FGFR2 glioblastoma: MRI and histology of the high-grade tumors.** Figure S3**. FGFR2 glioblastoma autopsy: gross appearance of sectioned brain.** Figure S4**. FGFR2 genomic locus and alterations in glioblastoma.** Figure S5**. FGFR3 carboxyl-terminal duplication mutation.** Figure S6**. FGFR glioblastoma expression analysis: overexpression ranking in 10 functional gene expression categories.** Figure S7**. Expression heatmaps: ECM, growth factor and cell adhesion/organization genes.** Figure S8**. Proteomic quantification.** Table S1**. Antibodies.** Table S2**. Mutations.** Table S3**. Fusions.** Table S4**. CNVs.

## Data Availability

Supporting data for this manuscript are available in the Supplemental Material and upon request to the corresponding author.

## Abbreviations

C-: Carboxyl
CDK: Cyclin-dependent kinase
CN: Copy number
CNS: Central nervous system
DDR: DNA damage response
DI: Diffusely infiltrating
DUSP: Dual-specificity phosphatase
ECM: Extracellular matrix
EGFR: Epidermal growth factor receptor
ERK/MAPK: Extracellular signal-regulated kinase/mitogen-activated protein kinase
FFPE: Formalin-fixed paraffin-embedded
FGFR: Fibroblast growth factor receptor
FLAIR: Fluid attenuated inversion recovery
GFAP: Glial fibrillary acidic protein
H&E: Hematoxylin eosin
HG: High grade
IDH: Isocitrate dehydrogenase
IHC: Immunohistochemistry
LG: Low grade
LM: Leptomeningeal
LOH: Loss of heterozygosity
MMP: Matrix metalloproteinase
MRI: Magnetic resonance imaging
N-: Amino
NGS: Next generation sequencing
PAI-1: Plasminogen activator inhibitor 1, encoded by *SERPINE1* gene
PDGFR: Platelet-derived growth factor receptor
PI3K p110a/*PIK3CA*: Phosphatidylinositol 3-OH kinase catalytic subunit alpha
PLCγ: Phospholipase C gamma
RTK: Receptor tyrosine kinase
SHH: Sonic Hedgehog
SPRY: Sprouty
TACC: Transforming acidic coiled-coil containing protein
TGF-β: Transforming growth factor β
TK: Tyrosine kinase
TLN1: Talin1
uPA: Urokinase plasminogen activator
W: Weighted (on MRI sequences T1 and T2)
WB: Western blot
WHO: World Health Organization
