# Supplementary material for: Novel targetable FGFR2 and FGFR3 alterations in glioblastoma associate with aggressive phenotype and distinct gene expression programs
Source: Acta Neuropathol Commun. 2021 Apr 14;9:69. doi: 10.1186/s40478-021-01170-1 (PMC8048363; doi:10.1186/s40478-021-01170-1)
Supplement: Supplementary file 1 — Additional file 1 Figure S1. FGFR3 glioblastomas: MRI and histology. Figure S2. FGFR2 glioblastoma: MRI and histology of the high-grade tumors. Figure S3. FGFR2 glioblastoma autopsy: gross appearance of sectioned brain. Figure S4. FGFR2 genomic locus and alterations in glioblastoma. Figure S5. FGFR3 carboxyl-terminal duplication mutation. Figure S6. FGFR glioblastoma expression analysis: overexpression ranking in 10 functional gene expression categories. Figure S7. Expression heatmaps: ECM, growth factor and cell adhesion/organization genes. Figure S8. Proteomic quantification. Table S1. Antibodies. Table S2. Mutations. Table S3. Fusions. Table S4. CNVs. [file 40478_2021_1170_MOESM1_ESM.pdf]

SUPPLEMENTAL FIGURES S1-S8

S1: FGFR3 glioblastomas: MRI and histology
S2: FGFR2 glioblastoma: MRI and histology of the two HG tumors
S3: FGFR2 glioblastoma autopsy: gross appearance of sectioned brain
S4: FGFR2 locus and alterations in glioblastoma
S5: FGFR3 CTdup mutation
S6: FGFR glioblastoma expression analysis: overexpression ranking
S7: Expression heatmaps: ECM, growth factor and cell adhesion/organization genes
S8: Proteomic quantification

SUPPLEMENTAL TABLES S1-S4

S1: Antibodies
S2: Mutations
S3: Fusions
S4: CNVs

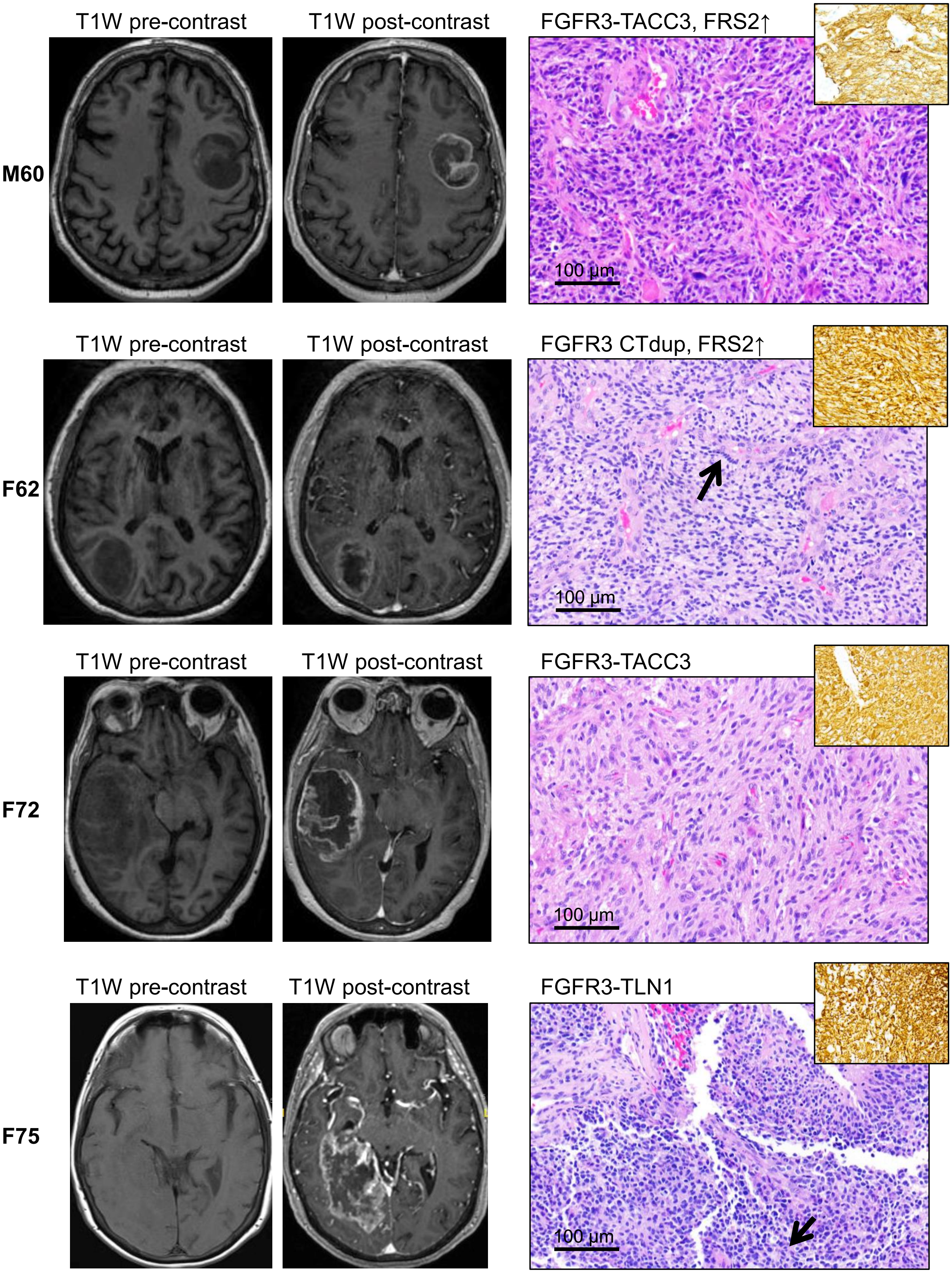

Supplemental Figure S1*.* **FGFR3 glioblastomas: MRI and recurrent morphologic features (RMFs).** T1W pre- and post-contrast images of the four FGFR3 glioblastoma rim-enhancing masses. H&Es show areas of previously described “FGFR3-TACC3 glioma RMFs”, such as monomorphous ovoid nuclei, endocrinoid capillary network, and pseudorosettes (arrow). The insets show strong diffuse GFAP positivity in all tumors.

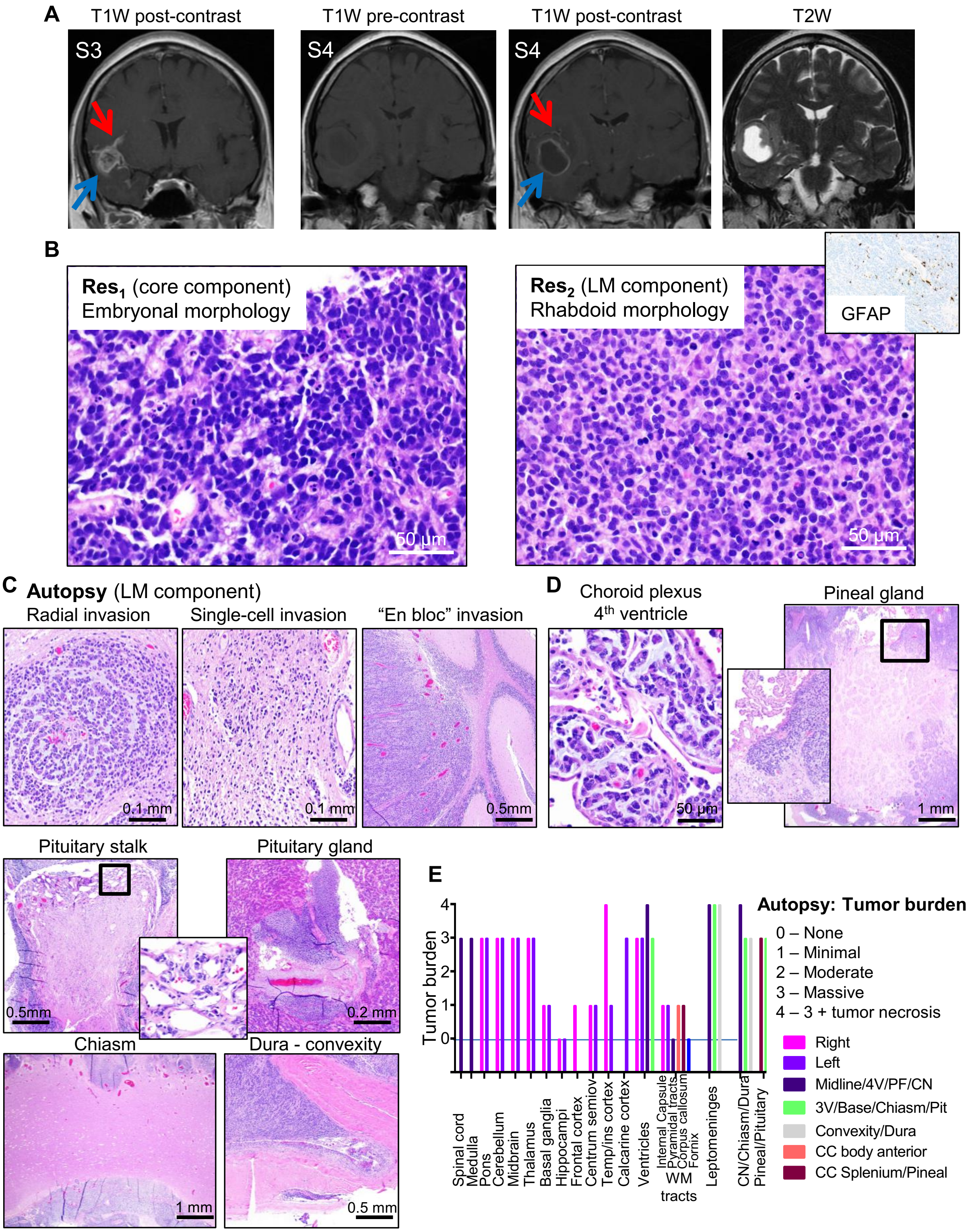

Supplemental Figure S2. **FGFR2 glioblastoma: MRI and histology of HG tumors.** **A.** Coronal MRI at two levels (S3 and S4) showing the rim-enhancing mass (blue arrows) and extension of enhancement to the Sylvian fissure (red arrows). T2W shows brain invasion surrounding the mass. **B.** H&E of the two different HG tumors: embryonal/HG neuroendocrine "blue-cell" morphology in the tumor core from Res_1, and rhabdoid cell morphology with loss of GFAP IHC in the LM component of Res_2. **C.** Routes of brain reinvasion by the LM neoplasm: (1) radial cell group invasion from Virchow-Robin spaces, (2) single-cell invasion, (3) "en bloc" invasion. **D.** Examples of brain structures invaded by the LM neoplasm: choroid plexus, pineal gland (inset of the box shown), pituitary stalk (inset of the box shown), pituitary gland, optic chiasm, dura mater. **E.** Semi-quantitative histologic analysis of tumor burden.

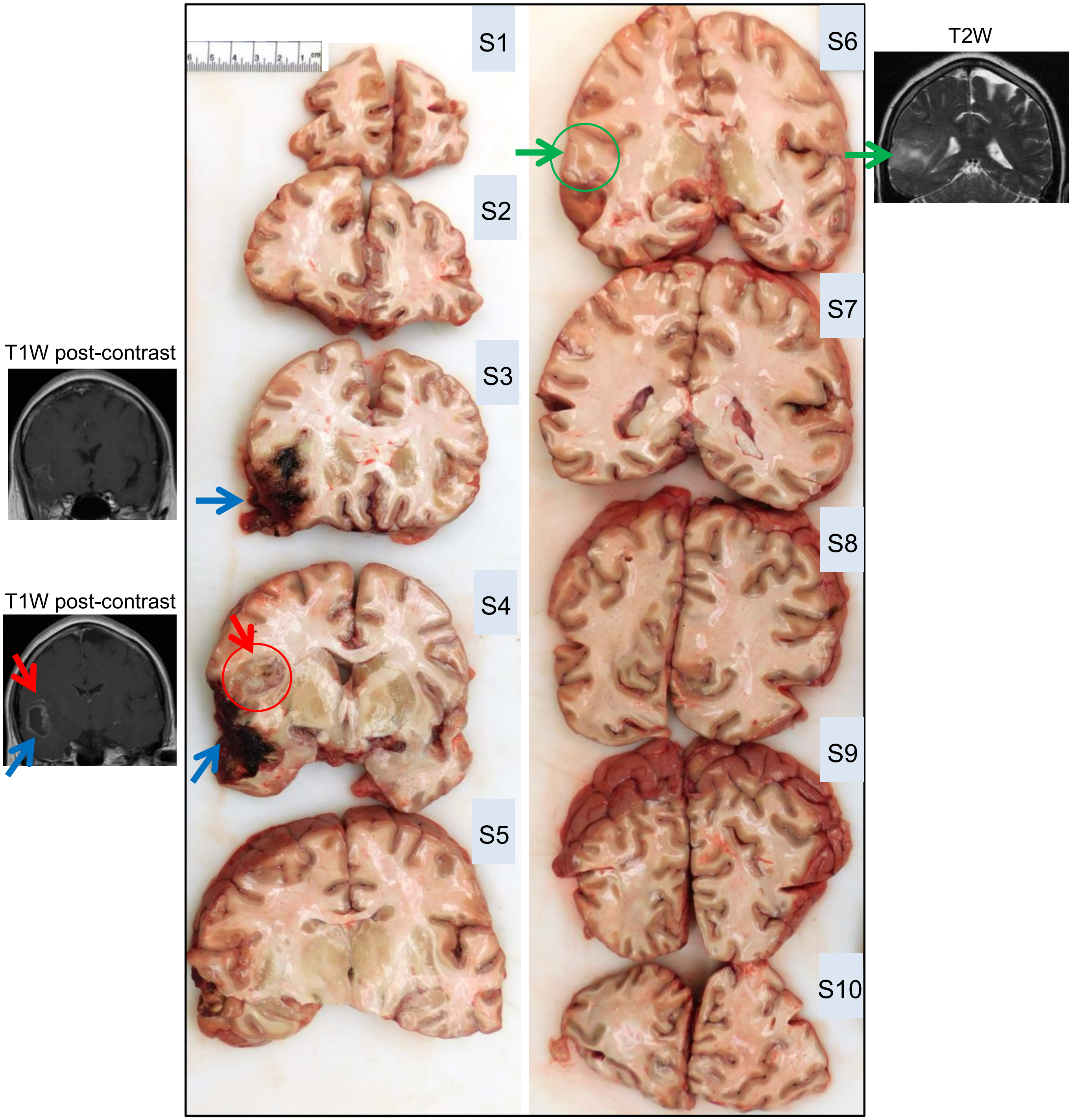

Supplemental Figure S3. **FGFR2 glioblastoma autopsy: gross appearance of the sectioned brain.** Fresh brain cutting shows the numbered sections (S1-S10) and the site of the previous resections (blue arrows). The following brain encircled areas were sampled for further analysis: the right Sylvian LM component shown with red arrow (LM_{S4}), and the right temporal intraparenchymal DI component shown with green arrow (DI_{S6}). Matched coronal MRI images acquired pre-operatory, 2.5 months pre-mortem are shown for the S3, S4 and S6 sections. Note aggressive tumor growth of LM_{S4} and presence of T2W hyperintensity corresponding to DI_{S6}.

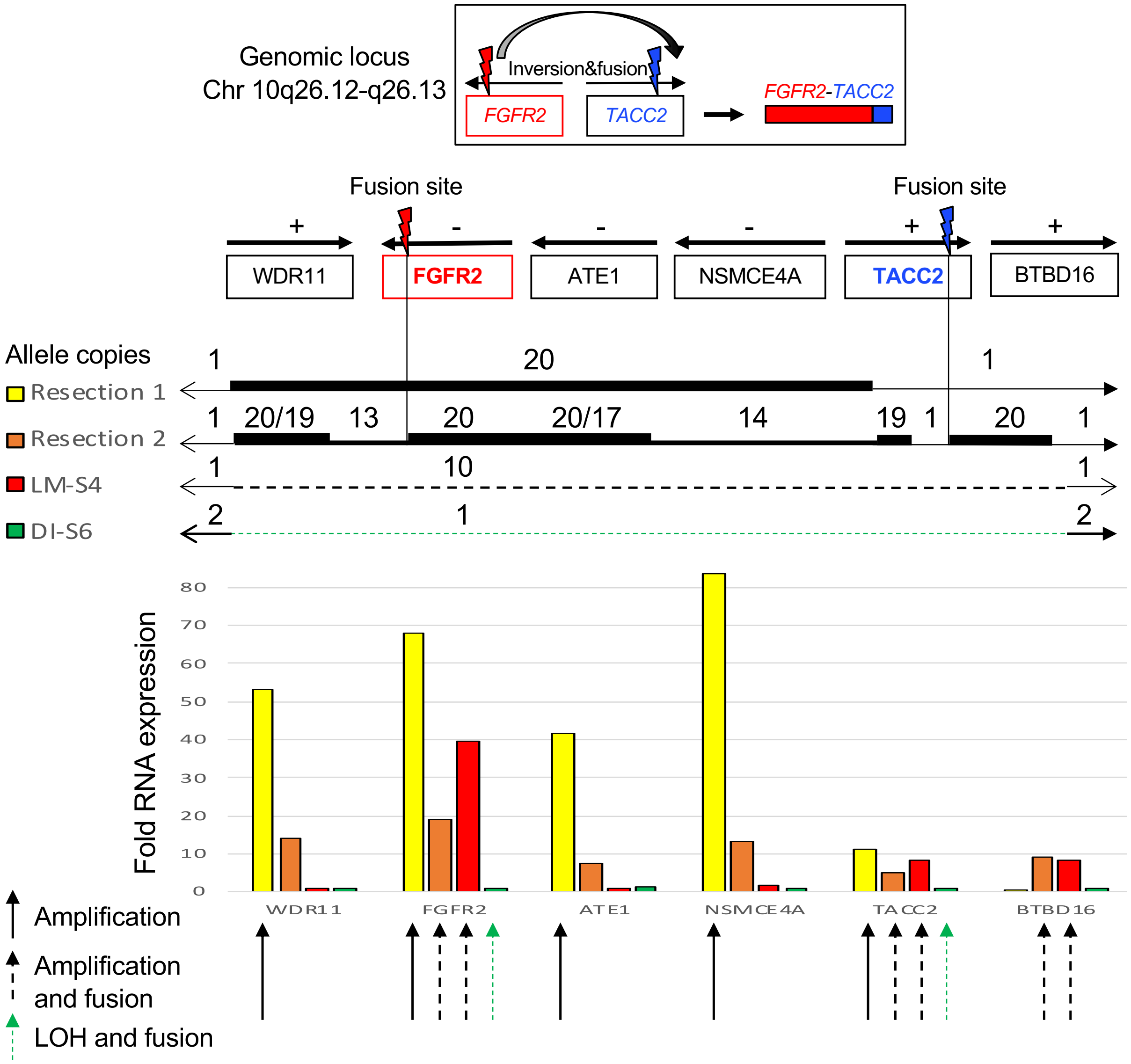

Supplemental Figure S4. *FGFR2* locus and alterations in glioblastoma. The genes of the *WDR11-FGFR2-TACC2* locus are boxed and their orientation on DNA is shown by upper arrows. The number of alleles is shown on top of each line of corresponding thickness for the samples indicated on the left. The CNV analysis used the whole exome for Res_1 and Res_2 specimens, hence the detailed analysis of all the genes of the locus, and the 596-gene panel for LM_{S4} and DI_{S6} autopsy samples, for which only the *FGFR2* gene was analyzed and the locus is represented by dotted lines. LOH was present for the whole locus and flanking regions in all HG samples, and only for *FGFR2* gene in DI_{S6}. Whole transcriptome analysis showed *FGFR2* highly expressed in all HG samples, and variable expression for the other genes of the locus, correlating with the presence or absence of amplified *FGFR2-TACC2* fusion in the HG samples. *FGFR2-TACC2* fusion with LOH and without amplification (green dotted line and arrows) was present in DI_{S6}. The mechanism of fusion involved inversion, as *FGFR2* and *TACC2* genes map to negative and positive DNA strands of the 10q26.13 chromosomal locus, respectively, and in-frame fusion. The Res_2 specimen contains a mixture of both HG tumors: core (embryonal/HG neuroendocrine morphology) and LM (rhabdoid morphology).

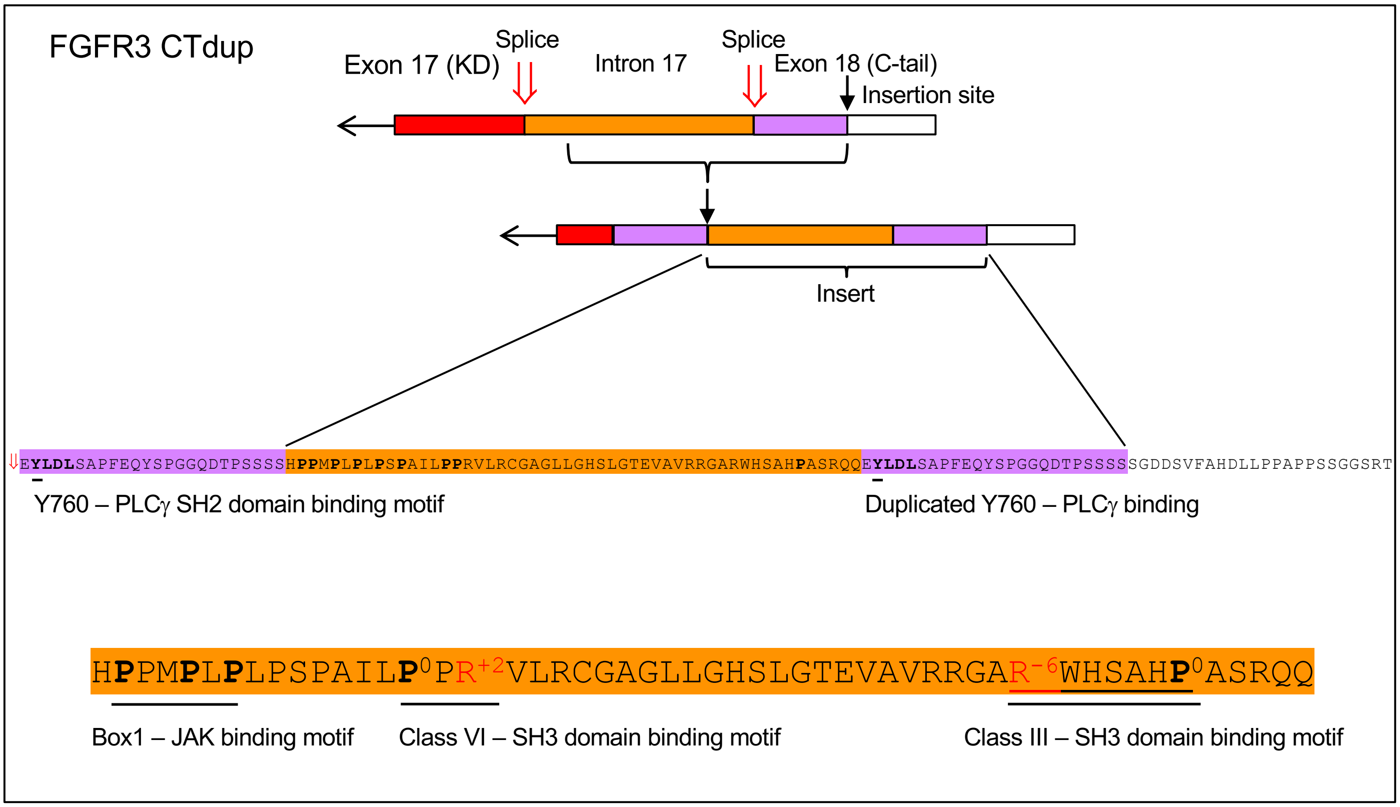

Supplemental Figure S5. <u>FGFR3 CTdup mutation</u>. Diagram of the *FGFR3* genomic locus showing the exon 17, encoding the kinase domain (KD), and exon 18, encoding FGFR3 C-tail (CT), with splice sites. The FGFR3 CTdup mutant resulted from an in-frame insertion of 52 amino acids encoded by most of intron 17, in orange, followed by 24 amino acids of the CT, in purple, into the insertion site marked by arrow, leading to the duplication of this CT segment. The CT sequence duplication resulted in the duplication of the PLCγ SH2 domain binding site (phosphorylated Y760). The new intronic sequence is Proline-rich (prolines shown in bold), and contains a putative PxxPxP (Box 1) JAK binding motif and two non-canonical SH3 domain binding motifs, class VI: XXXP^0XR^{+2}, and class III: R^{-6}XXXXXP^0.

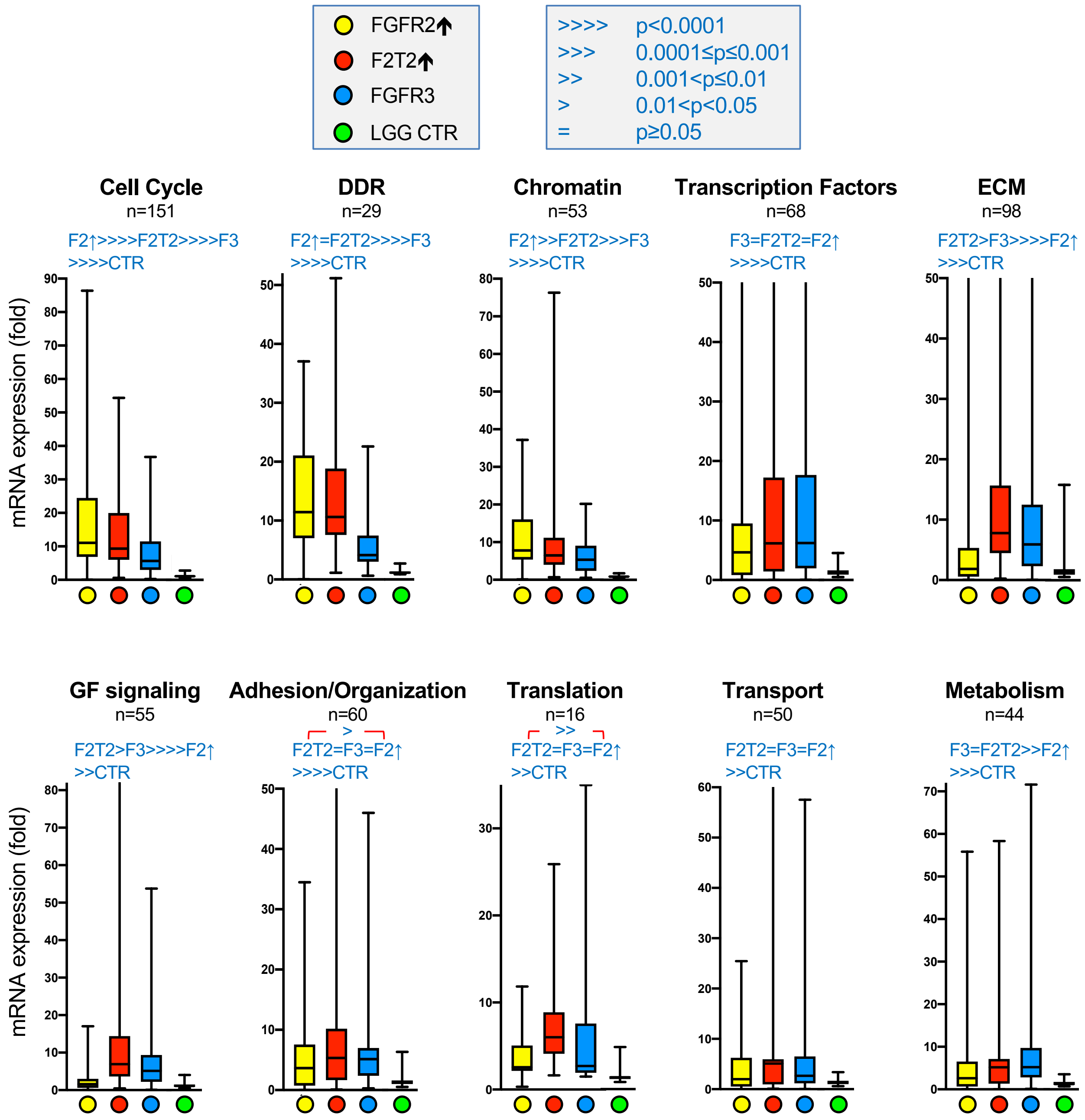

Supplemental Figure S6. <u>FGFR glioblastoma expression analysis</u>. Overexpression ranking for the functional gene categories shown on top and represented as box-and-whiskers plots of the overexpressed genes for the glioblastoma subgroups indicated with color-coded symbols on the x-axis. The box represents the median and quartiles, and the whiskers, the minimum and maximum values. Median ranking and statistical significance for the glioblastoma categories is shown on top of each chart, as in p-value legend. Red braces are shown for statistical significance in nonadjacent tumors.

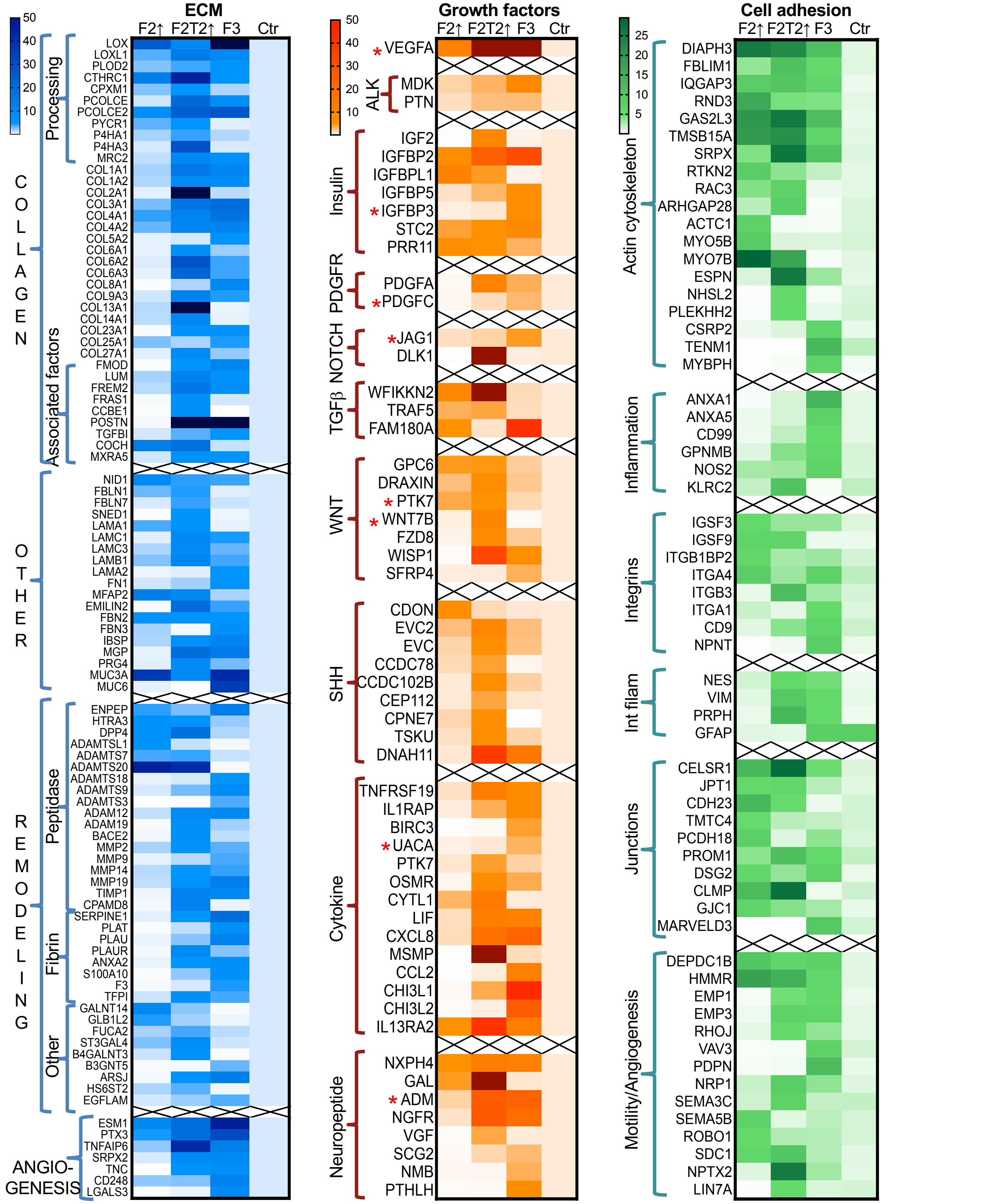

Supplemental Figure S7. ECM, growth factor and cell adhesion/organization expression heatmaps. Heatmaps showing ≥5-fold gene overexpression for the indicated gene categories. ECM was subclassified in four subsets related to collagenous or non-collagenous constituents and to remodeling or angiogenesis. The growth factor category was subdivided in signaling pathways. Asterisks mark genes involved in angiogenesis. The cell adhesion/organization category was subclassified into structural components. *GFAP* expression is shown for comparison, and is not included in the overexpression gene analysis.

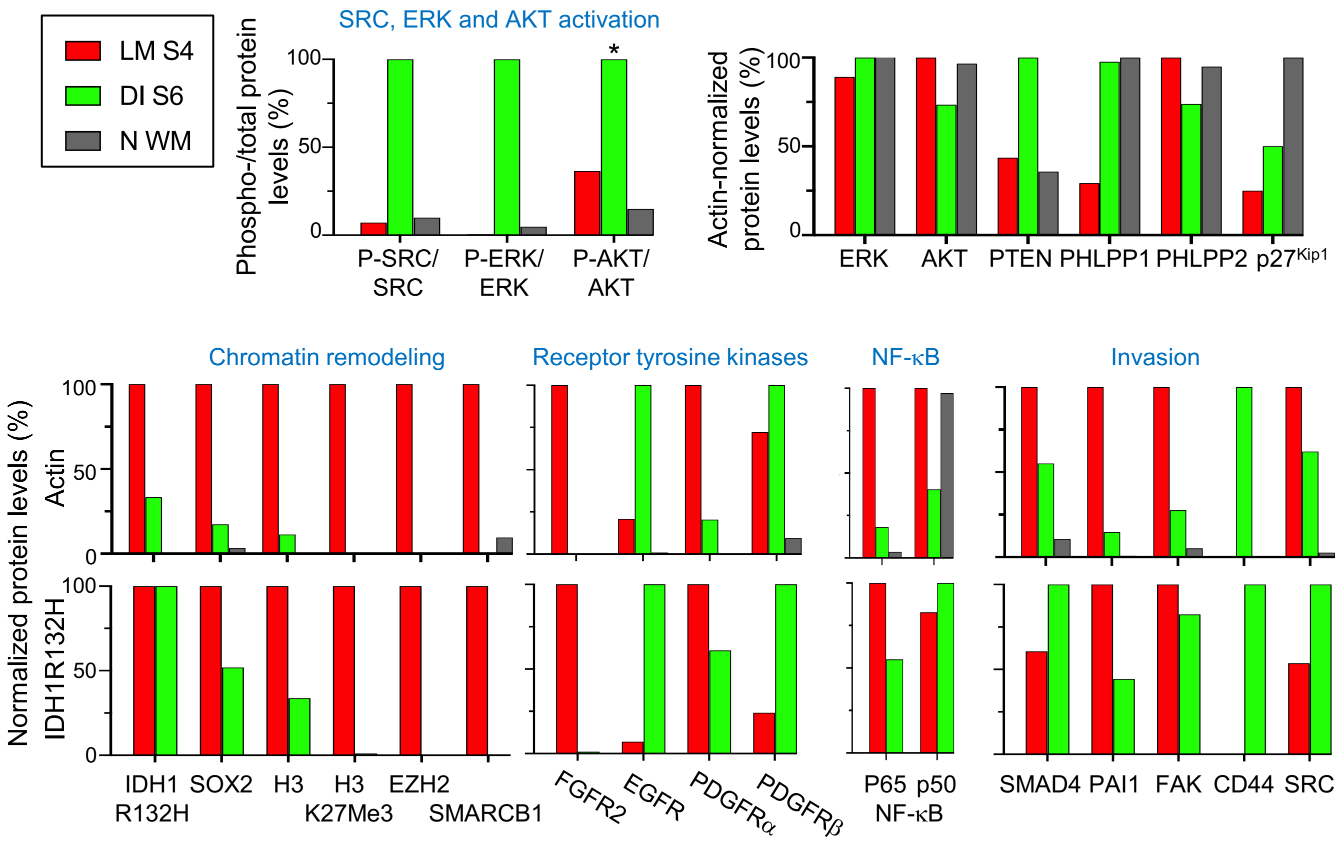

Supplemental Figure S8. **Quantitative protein expression analysis.** Bar graphs represent the indicated protein levels from autopsy LM_{S4} and DI_{S6} tumors and from normal white matter (N WM) control. The quantification of the WB bands was performed by densitometric analysis, as described in Materials and Methods. The phosphoprotein P-SRC, P-ERK and P-AKT values were normalized to the corresponding total unphosphorylated protein values, as indicated. P-AKT values are marked with an asterisk to indicate very low absolute values, and therefore minimal activation for the AKT pathway. As DI_{S6} contained a mixture of neoplastic and non-neoplastic cells, whereas LM_{S4} contained predominantly neoplastic cells, protein expression for the other individual densitometric values was normalized to both actin and IDH1-R132H levels, the latter reflecting better the content of neoplastic cells. Results are expressed as % of the highest expression value for a given protein. The pathways corresponding to each graph are indicated in blue on top of the graph.

Supplemental Table S1. Primary antibodies for WB.

Antibody	Cat. No.	Source	Type	MW kDA
β-Actin (AC15)	A5441	Sigma-Aldrich (St Louis, MO)	Mouse mAb	42
Akt (pan) (C67E7)	4691	Cell Signaling Technology (Danvers, MA)	Rabbit mAb	60
Phospho-Akt (Ser473) (D9E)	4060	Cell Signaling Technology (Danvers, MA)	Rabbit mAb	60
CD44 (156-3C11)	3570	Cell Signaling Technology (Danvers, MA)	Mouse mAb	80
EGFR (D38B1)	4267	Cell Signaling Technology (Danvers, MA)	Rabbit mAb	175
Phospho-EGFR (Tyr1068) (D7A5)	3777	Cell Signaling Technology (Danvers, MA)	Rabbit mAb	175
ERK (pan)	E17120/L1	BD Transduction Laboratories (San Jose, CA)	Mouse mAb	44/42
Phospho-p44/42 MAPK (Erk1/2) (Thr202/Tyr204) (D13.14.4E)	4370	Cell Signaling Technology (Danvers, MA)	Rabbit mAb	44/42
EZH2 (D2C9)	5246	Cell Signaling Technology (Danvers, MA)	Rabbit mAb	98
FAK/PTK2	3285	Cell Signaling Technology (Danvers, MA)	Rabbit pAb	125
Phospho-FAK (Tyr397)	3283	Cell Signaling Technology (Danvers, MA)	Rabbit mAb	125
FGFR1 (D8E4)	9740	Cell Signaling Technology (Danvers, MA)	Rabbit mAb	92, 120, 145
FGFR2 (D4H9)	11835	Cell Signaling Technology (Danvers, MA)	Rabbit mAb	92, 145
FGFR3 (C51F2)	4574	Cell Signaling Technology (Danvers, MA)	Rabbit mAb	125, 145, 165
FGFR4 (A-10)	sc-136988	Santa Cruz Biotechnology, Inc. (Dallas, TX)	Mouse mAb	95 - 125
Histone H3 (D1H2)	4499	Cell Signaling Technology (Danvers, MA)	Rabbit mAb	17
Tri-Methyl-Histone H3 (Lys27) (C36B11)	9733	Cell Signaling Technology (Danvers, MA)	Rabbit mAb	17
IDH1-R132H	DIA- H09	Dianova GmbH, Germany	Mouse mAb	44
INI-1/SMARCB1 (MRQ-27)	272M-16	Cell Marque (Rocklin, CA)	Mouse mAb	44
MET (D1C2)	8198	Cell Signaling Technology (Danvers, MA)	Rabbit mAb	145
MMP16 (13H7L7)	701306	Thermo Fisher Scientific (Rockford, IL)	Rabbit mAb	55
NF-κB p65/RELA (C22B4)	4764	Cell Signaling Technology (Danvers, MA)	Rabbit mAb	65
NF-κB1 p105/p50	3035P	Cell Signaling Technology (Danvers, MA)	Rabbit pAb	105 & 50
p16 INK4A	10883-1-AP	Proteintech (Chicago, IL)	Rabbit pAb	16
p27 Kip1 (D69C12)	3686	Cell Signaling Technology (Danvers, MA)	Rabbit mAb	27
p53(DO-7)	48818	Cell Signaling Technology (Danvers, MA)	Mouse mAb	53
PAI-1/SERPINE1 (C-9)	sc-5297	Santa Cruz Biotechnology (Dallas, TX)	Mouse mAb	48
PDGFRα (D1E1E)	3174	Cell Signaling Technology (Danvers, MA)	Rabbit mAb	190
PDGFRβ (28E1)	3169	Cell Signaling Technology (Danvers, MA)	Rabbit mAb	190
PHLPP1	A300-660A	Bethyl Laboratories (Montgomery, TX)	Rabbit pAb	185
PHLPP2	A300-661A	Bethyl Laboratories (Montgomery, TX)	Rabbit pAb	185
PTEN (138G6)	9559	Cell Signaling Technology (Danvers, MA)	Rabbit mAb	54
SMAD4 (D3R4N)	46535	Cell Signaling Technology (Danvers, MA)	Rabbit mAb	70
SOX2 (D6D9)	3579	Cell Signaling Technology (Danvers, MA)	Rabbit mAb	35
Src (32G6)	2123	Cell Signaling Technology (Danvers, MA)	Rabbit mAb	60
Phospho-Src Family (Tyr416) (D49G4)	6943	Cell Signaling Technology (Danvers, MA)	Rabbit mAb	60

mAb, monoclonal antibody; pAb, polyclonal antibody

Supplemental Table S3. Mutations.

Gene	Nucleotide	Amino acid	Effect	Other	NM	Case
BRCA2	c.7010C>T	T2337I	Missense	Germline	_000059	F48
BRCA2	c.8294G>T	C2765F	Missense	Somatic	_000059	F48
IDH1	c.395G>A	R132H	Missense	Somatic	_001282387	F48
TP53	c.695T>G	I232S	Missense	Somatic	_000546	F48
ATRX	c.6755A>G	H2252R	Missense	Somatic	_000489	F48
SERPINE1	c.629G>A	R210H	Missense	Somatic	_000602	F48
MMP16	c.868T>C	Y290H	Missense	Somatic	_005941	F48
PIK3CA	c.1633G>A	E545K	Missense	Somatic	_006218	F48
PTEN	c.47_48dupAT	Q17fs	Frameshift	Somatic	_000314	M60
PTEN	c.464A>G	Y155C	Missense	Somatic	_000314	F72
PTEN	c.743_744del	P248fs	Frameshift	Somatic	_000314	F75
PTEN	c.750_751del	C250fs	Frameshift	Somatic	_000314	F75
TERT	c.-124C>T		Promoter	Somatic	_198253	M60; F72
BRIP1	c.2392C>T	R798*	Nonsense	Somatic	_032043	M60
MSH6	c.3018C>G	Y1006*	Nonsense	Somatic	_000179	M60
MUTYH	c.1187G>A	G396D	Splice	Likely Germline	_001128425	F62
SOX9	c.499_501del	K167del	Inframe del	Likely Germline	_000346	F62
FGFR3	c.2274+26_2346dup	S782_S783ins(76)^	Inframe ins	Somatic	_000142	F62

^HPPMPLPLPSPAILPPRVLRCGAGLLGHSLGTEVAVRRGARWHSAHPASRQQ**EYLDLSAPFEQYSPGGQDTPSSSS**

The amino acid sequence in bold is duplicated in the mutant FGFR3

HNF1A	c.872dupC	G292fs	Frameshift	Somatic	_000545	F72
BCL10	c.262C>T	R88*	Nonsense	Likely Germline	_003921	F72
RB1	c.1352G>A	R451H	Missense	Likely Germline	_000321	F72
ATM	c.1402_1403del	K468fs	Frameshift	Likely Germline	_000051	F75
STAG2	c.3448C>T	Q1150*	Frameshift	Likely Germline	_001042750	F75
ADA	c.424C>T	R142*	Nonsense	Likely Germline	_000022	F75
DEPDC5	c.2714G>A	W905*	Nonsense	Somatic	_001242896	F75
ESPL1	c.5047G>T	E1683*	Nonsense	Somatic	_012291	F75
CHD4	c.3598C>T	R1200W	Missense	Somatic	_001273	F75
NPAS2	c.363G>A	P121P	Splice	Somatic	_002518	F75

Supplemental Table S2. RNA fusions.

Shading of in-frame fusions: grey-sample specific; blue-common to F48 high-grade tumors; green-common to different cases; yellow-FGFR fusions or FGFR2

F48 Common non-coding fusions: purple shading
* Genes with 5 copies or more in Resections 1-2
aa, amino acid

Fusions

FGFR2 exon	TACC2 exon	FGFR2 aa	TACC2 aa	Fusion peptide	F48
16	17	E767	R2710	LTLTTNE/REAAH	

FGFR3 exon	TACC3 exon	FGFR3 aa	TACC3 aa	Fusion peptide	M60
17	8	D758	F549	DLDRVLTVTSTD/FKESALRKQSLY	

FGFR3 exon	TACC3 exon	FGFR3 aa	TACC3 aa	Fusion peptide	F72
17	10	D758	V613	DLDRVLTVTSTD/VPGPPPGVPPPG	

FGFR3 exon	TLN1 exon	FGFR3 aa	TLN1 aa	Fusion peptide	F75
17	28	D758	V1188	DLDRVLTVTSTD/VAKAVTQALNRCV	

RORB exon	SLC9A6 exon	RORB aa	SLC9A6 aa	Fusion peptide	Fusion ncl sequence
1	11	R2	G351	MR/GVVAVLFC	ATGCGAG/GTGTA

SVIL exon	RSU1exon	SVIL region	RSU1 aa	RSU1 initial peptide	RSU1 peptide with Met
1	8	regulatory	G200	GLNDL	27-residue C-terminal

F48

Resection 1

gene_5	gene_3	chr_5	chr_3	bpt_5	bpt_3	fr_infr	fr_spr	hq_sp	signif_	spn_l	topo
SSU72	NADK	chr1	chr1	1564604	1765447	0	1	6	1	1	dup
chr4_q25	ANK2	chr4	chr4	112706218	113174415	0	1	4	1	1	del
PTN	MOB4:HSPE1:	chr7	chr2	137300977	197500344	0	0	28	1	3	tloc
BOP1	UBA3	chr8	chr3	144265009	69061850	0	0	5	1	1	tloc
chr9_p21.2	EQTN	chr9	chr9	27277550	27286363	1	1	2	1	1	dup
RORB	SLC9A6	chr9	chrX	74497984	136013348	1	1	4	1	2	tloc
chr10_p15.2	RSU1	chr10	chr10	3256459	16695156	1	1	42	1	1	dup
ANKRD16*	PIP4K2A	chr10	chr10	5864402	22688303	0	1	2	1	2	dup
ANKRD16*	chr10_p12.2	chr10	chr10	5878096	24146111	0	1	3	1	3	dup
ANKRD16*	ABI1	chr10	chr10	5878096	26777242	0	1	3	1	1	dup
ANKRD16*	chr10_p15.1	chr10	chr10	5878096	4144835	0	0.99	85	4	6	inv
ANKRD16*	CAMK1D	chr10	chr10	5878096	12389585	0	1	69	5	10	inv
RSU1	chr10_p14	chr10	chr10	16695022	10746247	0	1	3	1	2	del
RSU1	chr10_p13	chr10	chr10	16695022	17234832	0	1	3	1	1	dup
RSU1	chr10_p13	chr10	chr10	16695022	17249910	0	1	11	1	1	dup
RSU1	chr10_p12.31	chr10	chr10	16695022	18729869	0	1	9	1	1	dup
RSU1	chr10_p12.31	chr10	chr10	16695022	18736215	0	1	0	0	1	dup
RSU1	chr10_p12.31	chr10	chr10	16695022	18739238	0	1	0	0	1	dup
RSU1	chr10_p12.31	chr10	chr10	16695022	18846387	0	1	2	0	1	dup
RSU1	chr10_p14	chr10	chr10	16695022	7528974	0	0.99	404	3	10	inv
ABI1*	chr10_p15.1	chr10	chr10	26747358	5896935	0	0.75	2	0	2	del
SVIL	RSU1	chr10	chr10	29705719	16695063	0	0.67	3	0	2	del
SVIL	RSU1	chr10	chr10	29735754	16695156	0.95	1	283	5	7	del
SVIL	ABI1*	chr10	chr10	29735754	26777242	1	1	12	1	3	del
ZNF248	chr10_p14	chr10	chr10	37832194	9571130	0	0.25	6	1	2	del
chr10_q21.1	IPO7	chr10	chr11	59244006	9436270	0	1	4	1	1	tloc
chr10_q26.1:	chr10_q26.13	chr10	chr10	120768476	122202586	0	0	0	0	0	dup
chr10_q26.1:	chr10_q26.13	chr10	chr10	120812038	122327974	0	0	0	0	0	dup
chr10_q26.1:	chr10_q26.13	chr10	chr10	120812034	122463068	0	0	0	0	1	dup
chr10_q26.1:	TACC2*	chr10	chr10	121313215	122132608	0	1	4	1	1	inv
FGFR2*	CAMK1D	chr10	chr10	121515122	12389585	0	1	4	1	1	inv
FGFR2*	chr10_q26.12	chr10	chr10	121551289	121304944	0	1	7	1	4	inv
FGFR2*	chr10_q26.13	chr10	chr10	121538591	121967717	0	1	4	1	1	inv
FGFR2*	chr10_q26.13	chr10	chr10	121538591	121976287	0	1	2	0	1	inv
ATE1*	chr10_q26.12	chr10	chr10	121836717	121064169	0	1	0	0	1	del
ATE1*	WDR11*	chr10	chr10	121836715	120858720	0	1	3	0	2	inv
ATE1*	WDR11*	chr10	chr10	121790174	120900026	1	1	0	0	1	inv
ATE1*	TACC2*	chr10	chr10	121790168	122132608	0	1	18	1	6	inv
ATE1*	chr10_q26.12	chr10	chr10	121898840	121064169	0	1	7	1	2	del
ATE1*	WDR11*	chr10	chr10	121898840	120858642	0.71	0.86	6	1	3	inv

ATE1*	TACC2*	chr10	chr10	121898905	122132731	0	0.86	4	0	4	inv
FBXO18	chr10_p12.1	chr10	chr10	5895188	26759020	0	1	13	1	8	del
ARMC3	chr10_p14	chr10	chr10	22981701	10089318	0	1	3	1	2	dup
KIAA1217	ABI1*	chr10	chr10	24141847	26777242	0	1	4	1	1	inv
chr10_p12.1	ABI1*	chr10	chr10	29235348	26777242	0	1	4	1	3	inv
WDR11*	ATE1*	chr10	chr10	120852636	121841264	0.68	1	18	2	4	inv
WDR11*	ATE1*	chr10	chr10	120866765	121913894	1	1	0	0	1	inv
WDR11*	ATE1*	chr10	chr10	120906676	121911101	0	0.5	1	0	2	inv
TACC2*	chr2_p24.3	chr10	chr2	122022015	16295125	0	1	2	1	2	tloc
TACC2*	chr2_q31.1	chr10	chr2	122195177	172700970	0	1	594	2	3	tloc
TACC2*	chr10_q26.12	chr10	chr10	122195177	121064169	0	1	4	1	2	inv
TACC2*	chr10_q26.12	chr10	chr10	122195177	121313362	0	1	3	1	1	inv
TACC2*	chr10_q21.2	chr10	chr10	122195177	59743715	0	1	3	1	1	dup
TACC2*	chr10_q26.12	chr10	chr10	122195177	120752895	0	0.57	12	2	12	dup
TACC2*	chr10_q26.12	chr10	chr10	122195177	120812030	0	0.99	1855	4	45	dup
TACC2*	chr10_q26.12	chr10	chr10	122195177	120824385	0	1	20	2	4	dup
TACC2*	chr10_q26.12	chr10	chr10	122195177	120851079	0	1	4	1	3	dup
TACC2*	WDR11*	chr10	chr10	122195177	120858642	0.3	1	4	1	3	dup
TACC2*	WDR11*	chr10	chr10	122088559	120900026	0.17	1	1	0	3	dup
TACC2*	chr17_q12	chr10	chr17	122195177	39333196	0	0.98	54	1	3	tloc
CEP112:AXI	WFIKKN2	chr17	chr17	65549519	50839498	0	1	4	1	3	inv
HELZ	chr17_q22	chr17	chr17	67215898	53450911	0	1	2	1	2	del
PRKCA*	chr17_q24.2	chr17	chr17	66306128	66220135	0	1	14	1	1	dup
SAP30BP	CA10	chr17	chr17	75671850	51747812	0	0	0	0	0	inv
SAP30BP	CA10	chr17	chr17	75671864	51931133	0	0.96	18	1	3	inv
LRRC75B	chr8_q11.21	chr22	chr8	24588213	48077406	0	1	3	1	1	tloc
TMEM164	chrX_q23	chrX	chrX	110109147	112962582	0	1	8	1	2	del
TMEM164	chrX_q23	chrX	chrX	110109147	112985240	0	1	7	2	4	del
TMEM164	chrX_q23	chrX	chrX	110109147	113048853	0	1	2	0	2	del
PAK3	OSTF1	chrX	chr9	111013901	75117503	1	1	5	1	2	tloc

Resection 2

gene_5	gene_3	chr_5	chr_3	bpt_5	bpt_3	fr_infr	fr_spr	hq_sp	signif_	spn_l	topo
SSU72	NADK	chr1	chr1	1564604	1765447	0	1	8	1	2	dup
YARS	chr1_p31.3	chr1	chr1	32817187	63857968	0	1	0	0	1	dup
ROR1	S100PBP	chr1	chr1	63866854	32817113	0	0	3	1	1	dup
PECR:MREG	chr2_q35	chr2	chr2	215996305	217220856	0	1	1	0	1	dup
PECR:MREG	chr2_q35	chr2	chr2	215996305	217268465	0	1	8	1	2	dup
RANBP2	RGPD5	chr2	chr2	108753564	109825875	0.8	0.8	2	1	3	del
ZNF354B	ZNF354A	chr5	chr5	178866165	178727126	0	1	3	1	2	inv
PTN	MOB4:HSPE1:	chr7	chr2	137300977	197500344	0	0	14	1	1	tloc
RORB	SLC9A6	chr9	chrX	74497984	136013348	0.57	1	4	1	4	tloc
chr10_p15.2	RSU1	chr10	chr10	3256459	16695156	1	1	11	1	1	dup
ANKRD16*	chr10_p15.1	chr10	chr10	5878096	4144835	0	0.95	48	3	5	inv
ANKRD16*	CAMK1D	chr10	chr10	5878096	12389585	0	1	30	3	8	inv
RSU1	chr10_p14	chr10	chr10	16695022	7528974	0	0.97	23	1	7	inv
ABI1*	chr10_p15.1	chr10	chr10	26751697	5895638	0	0	0	0	1	del
SVIL	RSU1	chr10	chr10	29705720	16695137	0	0	2	0	2	del
SVIL	RSU1	chr10	chr10	29735754	16695156	0.94	1	18	2	5	del
chr10_q26.1:	chr10_q26.13	chr10	chr10	120808258	122195152	0	0	0	0	0	dup
FGFR2*	chr10_q26.13	chr10	chr10	121483697	122056083	0	1	8	1	1	dup
FGFR2*	chr10_q26.12	chr10	chr10	121483735	121368985	0	0	3	1	1	inv
FGFR2*	TACC2*	chr10	chr10	121483697	122237394	0.99	0.99	702	3	8	inv
FBXO18	chr10_p12.1	chr10	chr10	5890347	26759020	0	0.9	11	1	10	del
ARMC3*	chr10_p14	chr10	chr10	22981701	10090667	0	1	5	1	1	dup
TACC2*	chr2_q31.1	chr10	chr2	122195177	172700970	0	1	30	1	2	tloc
BTBD16*	FGFR2*	chr10	chr10	122295380	121539000	0	0.5	6	1	4	inv
TACC2*	chr10_q26.12	chr10	chr10	122195131	120752892	0	0.5	2	0	2	dup
TACC2*	chr10_q26.12	chr10	chr10	122195177	120812030	0	0.99	44	2	9	dup
TUBA1C	TRIM9	chr12	chr14	49272686	51026593	0	0	4	1	1	tloc
CEP112:AXII	WFIKKN2	chr17	chr17	65549519	50839498	0	1	3	1	1	inv
HELZ	chr17_q22	chr17	chr17	67215898	53437378	0	1	1	0	1	del
HELZ	chr17_q22	chr17	chr17	67215898	53450911	0	1	3	1	1	del
HELZ	chr17_q22	chr17	chr17	67215898	53549657	0	1	3	0	2	del
DLX4	chr17_q24.3	chr17	chr17	49969752	70897671	0	0.95	17	1	3	del
PRKCA*	chr17_q24.2	chr17	chr17	66306128	66220135	0	1	6	1	1	dup
SAP30BP	CA10	chr17	chr17	75671864	51747816	0	0	0	0	0	inv
SAP30BP	CA10	chr17	chr17	75671864	51931133	0	1	22	1	2	inv
chrX_p22.2	MID1	chrX	chrX	11055935	10567604	1	1	6	1	2	del
TMEM164	chrX_q23	chrX	chrX	110067356	112962653	0	1	2	0	3	del
TMEM164	chrX_q23	chrX	chrX	110109147	112991543	0	1	9	1	4	del
TMEM164	chrX_q23	chrX	chrX	110109147	113073082	0	1	9	1	3	del

LM-S4

gene_5	gene_3	chr_5	chr_3	bpt_5	bpt_3	fr_infr	fr_spr	hq_sp	signif_	spn_l	topo
SSU72	NADK	chr1	chr1	1564604	1765447	0	1	13	1	1	dup
YARS	chr1_p31.3	chr1	chr1	32817187	63857968	0	1	3	1	1	dup
MEX3A	chr9_q34.3	chr1	chr9	156077067	135973361	0	1	3	1	1	tloc
ROR1	S100PBP	chr1	chr1	63866854	32817113	0	0	0	0	1	dup
chr2_p24.3	chr10_p15.1	chr2	chr10	16003443	5882648	0	0	0	0	1	tloc
ANKRD36B	ANKRD36	chr2	chr2	97547535	97194725	1	1	3	1	2	inv
RGPD8	RGPD3	chr2	chr2	112399631	106435203	1	1	4	1	1	del
NCKAP1	LMCD1	chr2	chr3	182942103	8550624	0	0	3	1	1	tloc
PECR:MREG	chr2_q35	chr2	chr2	215996305	217268465	0	1	17	1	3	dup
chr3_q13.32	LSAMP	chr3	chr3	117997181	116444956	1	1	5	1	1	del
chr3_q13.32	LSAMP	chr3	chr3	117997193	117009235	0	0	0	0	0	del
TTC33	PRKAA1	chr5	chr5	40746797	40777587	0	1	4	1	2	dup
ZNF354B	ZNF354A	chr5	chr5	178866165	178727126	0	1	7	2	2	inv
PTN	MOB4:HSPE1:	chr7	chr2	137300977	197500344	0	0	13	1	2	tloc
chr10_p15.2	RSU1	chr10	chr10	3256459	16695156	0.9	0.9	4	1	2	dup
ANKRD16*	chr2_p24.3	chr10	chr2	5878096	16011172	0	1	2	1	1	tloc
ANKRD16*	chr10_p15.1	chr10	chr10	5878096	4144835	0	0.99	61	2	5	inv
ANKRD16*	CAMK1D	chr10	chr10	5878096	12389585	0	0.98	31	4	10	inv
RSU1	chr10_p14	chr10	chr10	16695022	7528974	0	1	41	2	5	inv
ABI1*	chr10_p15.1	chr10	chr10	26748004	5894037	0	1	1	0	1	del
SVIL	RSU1	chr10	chr10	29705719	16695063	0	0.33	3	0	2	del
SVIL	RSU1	chr10	chr10	29735754	16695156	1	1	36	3	3	del
FGFR2*	chr10_q26.13	chr10	chr10	121483697	122056083	0	1	17	1	1	dup
FGFR2*	TACC2*	chr10	chr10	121483697	122237394	0.98	0.99	2554	5	16	inv
FBXO18	chr10_p12.1	chr10	chr10	5895188	26759020	0	0.7	14	2	6	del
chr10_p11.2	ARMC3*	chr10	chr10	35790045	22955806	0	1	2	1	1	dup
MCU	chr20_q13.33	chr10	chr20	72715218	64241512	0	0	0	0	0	tloc
MCU	chr20_q13.33	chr10	chr20	72715903	64279394	0	1	3	1	1	tloc
BTBD16*	FGFR2*	chr10	chr10	122291195	121538716	0	0.77	11	3	3	inv
KLRC4:KLRK	ITPR2	chr12	chr12	10407635	26655853	0	1	3	1	1	dup
RND1	PDCL2	chr12	chr4	48860996	55582238	1	1	2	1	1	tloc
chr15_q23	RFWD3	chr15	chr16	72384415	74636578	0	1	4	1	1	tloc
KANSL1	LRRC37A	chr17	chr17	46170854	46330449	0	1	3	1	1	inv
CEP112:AXII	WFIKKN2	chr17	chr17	65549519	50839498	0	1	7	1	3	inv
DLX4	chr17_q24.3	chr17	chr17	49969752	70897671	0	1	21	1	2	del
PRKCA*	chr17_q24.2	chr17	chr17	66306128	66220135	0	1	13	1	1	dup
SAP30BP	CA10	chr17	chr17	75668622	51747756	0	0	0	0	0	inv
SAP30BP	CA10	chr17	chr17	75671864	51931133	0	1	37	1	2	inv
RPTOR	chr17_q21.33	chr17	chr17	80545792	50405349	0	1	6	1	2	inv
LAMA5	PIP5K1C	chr20	chr19	62346738	3661978	0	0	2	1	1	tloc
TMEM164	chrX_q23	chrX	chrX	110109147	112962582	0	1	4	1	3	del

TMEM164	chrX_q23	chrX	chrX	110109147	112992435	0	1	6	1	4	del
TMEM164	chrX_q23	chrX	chrX	110109147	113073082	0	1	6	2	2	del

DI-S6

gene_5	gene_3	chr_5	chr_3	bpt_5	bpt_3	fr_infr	fr_spr	hq_sp	signif_	spn_l	topo
SSU72	NADK	chr1	chr1	1564604	1765447	0	1	21	1	2	dup
CYP4Z1	CYP4X1	chr1	chr1	47068764	47046466	0	1	6	1	1	dup
RGPD8	RGPD3	chr2	chr2	112399631	106435203	1	1	8	1	1	del
TMEM163	chr2_q21.3	chr2	chr2	134713199	134869042	0	1	4	1	2	dup
STON1-GTF2	chr2_p16.3	chr2	chr2	48671681	49097431	0	1	4	1	1	del
chr3_q13.32	LSAMP	chr3	chr3	117932411	116086560	1	1	1	0	1	del
chr3_q13.32	LSAMP	chr3	chr3	117997181	116444956	0.9	1	14	1	2	del
chr3_q13.32	LSAMP	chr3	chr3	117997193	117009235	0	0	0	0	0	del
chr4_q25	ANK2	chr4	chr4	112706218	113174415	0	1	12	1	1	del
PTN	MOB4:HSPE1	chr7	chr2	137300977	197500344	0	0	3	1	1	tloc
PTN	MOB4:HSPE1	chr7	chr2	137327409	197501109	0	0	0	0	0	tloc
TNKS	XPO7	chr8	chr8	9766426	21966856	0	1	4	1	1	del
XPO7	chr8_p11.23	chr8	chr8	21999675	36814366	0	1	4	1	1	inv
ERMP1	KIAA2026	chr9	chr9	5787060	5914063	0	1	2	1	1	dup
FGFR2	TACC2	chr10	chr10	121483697	122237394	1	1	9	1	1	inv
PSPC1	ZMYM5	chr13	chr13	19730238	19835690	0.5	1	6	1	3	dup
PSPC1	ZMYM5	chr13	chr13	19782456	19852184	0	0	0	0	0	dup
ADAMTSL3	SH3GL3	chr15	chr15	83704509	83586982	1	1	5	1	2	dup
ADAMTSL3	SH3GL3	chr15	chr15	83899732	83586982	0	1	1	0	1	dup
SUPT5H	chr15_q25.3	chr19	chr15	39453522	85834762	0	1	2	1	1	tloc
chr19_q13.4	ZNF880	chr19	chr19	52392789	52373110	0	1	2	1	1	dup
ZNF528	ZNF880	chr19	chr19	52406644	52373110	0	1	3	1	2	dup
TMEM164	chrX_q23	chrX	chrX	110109147	112962582	0	1	5	1	2	del
TMEM164	chrX_q23	chrX	chrX	110109147	112992435	0	1	3	1	1	del
TMEM164	chrX_q23	chrX	chrX	110109085	113073081	0	0	0	0	0	del

M60

gene_5	gene_3	chr_5	chr_3	bpt_5	bpt_3	fr_infr	fr_spr	hq_sp	signif_	spn_l	topo
PLEKHM2	BLVRB	chr1	chr19	15684619	40458546	0	0.99	65	2	3	tloc
GPSM2	STXBP3	chr1	chr1	108885579	108794826	0	0.98	30	1	4	dup
RANBP2	RGPD5	chr2	chr2	108753564	109825875	1	1	3	1	1	del
chr3_q13.32	LSAMP	chr3	chr3	117932422	116086399	0	0	0	0	0	del
chr3_q13.32	LSAMP	chr3	chr3	117997181	116444956	0.92	1	8	1	2	del
chr3_q13.32	LSAMP	chr3	chr3	117997190	117009235	0	0	0	0	0	del
FGFR3	TACC3	chr4	chr4	1806935	1735730	0.95	0.95	45	1	3	dup
PLAT	chr8_p11.21	chr8	chr8	42191371	42233860	0	1	3	1	2	dup
IPO8	RASSF8	chr12	chr12	30649136	25995036	0	1	51	4	10	inv
chr12_q15	PTPRR	chr12	chr12	70168460	70684784	0	1	0	0	2	dup
chr12_q15	PTPRR	chr12	chr12	70184811	70684785	0	0	0	0	0	dup
chr12_q15	PTPRR	chr12	chr12	70242796	70684784	0.94	1	19	3	4	dup
FAR2	chr12_p11.22	chr12	chr12	29270639	28719912	0	1	4	1	1	dup
CPSF6	PNISR	chr12	chr6	69262563	99408272	0	1	3	1	2	tloc
CPSF6	CHMP1A	chr12	chr16	69262563	89647332	0	1	2	1	1	tloc
chr12_q15	CAPRIN2	chr12	chr12	70180539	30741107	0.84	1	30	4	7	inv
ACSS3	chr12_q15	chr12	chr12	81078402	68807839	0	0	0	0	0	dup
ACSS3	MDM2	chr12	chr12	81078432	68809207	0.88	0.88	6	1	2	dup
ACSS3	chr12_q15	chr12	chr12	81109705	69706005	0	1	2	1	1	inv
ACSS3	SLC35E3	chr12	chr12	81109705	68782280	0	1	28	2	3	dup
ACSS3	chr12_q15	chr12	chr12	81109641	68798625	0	0.5	2	0	2	dup
ACSS3	chr12_q15	chr12	chr12	81109705	68807842	0	1	78	1	1	dup
ACSS3	MDM2	chr12	chr12	81109705	68813553	0.01	0.98	664	4	12	dup
ACSS3	chr12_q15	chr12	chr12	81256176	68807845	0	0	1	0	1	dup
ACSS3	MDM2	chr12	chr12	81229167	68809226	0	0	1	0	1	dup

F72

gene_5	gene_3	chr_5	chr_3	bpt_5	bpt_3	fr_infr	fr_spr	hq_sp	signif_	spn_l	topo
ATP1A2	RALGAPB	chr1	chr20	160129063	38560388	0	0	3	1	1	tloc
DNAJC27	chr2_p23.3	chr2	chr2	24947632	25093084	0	0	0	0	0	dup
RGPD8	RGPD3	chr2	chr2	112399631	106435203	1	1	4	1	1	del
EFR3B	chr2_p23.3	chr2	chr2	25103788	24942736	0	1	5	1	1	dup
EFR3B	chr2_p23.3	chr2	chr2	25103788	24947633	0	1	9	2	3	dup
chr3_q13.2	FAM21A	chr3	chr10	111904016	50084071	0	1	2	1	1	tloc
FGFR3	TACC3	chr4	chr4	1806935	1737597	0.98	0.98	217	1	5	dup
chr4_q25	ANK2	chr4	chr4	112706218	113174415	0	1	2	1	1	del
ADAP1:COX	chr9_p21.3	chr7	chr9	969397	19969658	0	0	3	1	1	tloc
TTC17	ELOVL2	chr11	chr6	43405807	10983501	0	0	3	1	1	tloc
chr11_q13.1	DUSP1	chr11	chr5	65506029	172769769	1	0	4	1	1	tloc
TSFM:METTL	MARS	chr12	chr12	57773129	57512007	0	1	70	2	2	dup
PRKD1	chr14_q12	chr14	chr14	29725535	30419570	0	1	1	0	1	inv
PRKD1	chr14_q12	chr14	chr14	29725535	30480635	0	1	20	1	1	inv
FEM1B	LRRC8C:LRRC	chr15	chr1	68292857	89933293	0	0	4	1	1	tloc
ATP6V1E1	DNAH10	chr22	chr12	17628602	123767408	0	1	3	1	1	tloc

F75

gene_5	gene_3	chr_5	chr_3	bpt_5	bpt_3	fr_infr	fr_spr	hq_sp	signif_	spn_l	topo
IFI44	HIST2H4A	chr1	chr1	78659957	149832909	0	0	4	1	1	del
RGPD8	RGPD3	chr2	chr2	112399631	106435203	1	1	6	1	1	del
chr3_q13.32	LSAMP	chr3	chr3	117997179	116086540	0	0	0	0	0	del
chr3_q13.32	LSAMP	chr3	chr3	117997181	116444956	1	1	5	1	1	del
chr3_q13.32	LSAMP	chr3	chr3	117997190	117009235	0	0	0	0	0	del
FGFR3	TLN1	chr4	chr9	1806935	35712125	0.73	0.99	59	2	6	tloc
chr4_q25	ANK2	chr4	chr4	112706218	113174415	0	1	3	1	1	del
APC:SRP19:Z	REEP5	chr5	chr5	112707883	112902519	0	1	0	0	1	inv
APC:SRP19:Z	ZRSR2	chr5	chrX	112892924	15823003	0	0	3	1	1	tloc
PTN	MOB4:HSPE1:	chr7	chr2	137300977	197500344	0	0	14	1	1	tloc
EGFR	chr7_p11.2	chr7	chr7	55200414	54730107	0	0.71	4	1	2	dup
TLN1	MYO18A:TIAF	chr9	chr17	35711594	29090927	0.98	0.98	225	2	3	tloc
ZMIZ1	FCGBP	chr10	chr19	79306224	39875679	0	0	3	1	1	tloc
TACC2	chr2_q31.1	chr10	chr2	122195177	172700970	0	1	3	1	1	tloc
ITPR2	PCDHB11	chr12	chr5	26705988	141201818	0	0	3	1	1	tloc
chr12_q14.3	GRIP1	chr12	chr12	67069049	66541951	1	1	2	1	1	del
chr12_q14.3	GRIP1	chr12	chr12	67069044	66596878	0	0	0	0	0	del
CLCN7	chr22_q13.1	chr16	chr22	1457268	38247804	0	0	0	0	0	tloc
chr16_p11.2	TAOK2	chr16	chr16	30876119	29989592	1	1	5	1	1	dup
ST8SIA5	PIAS2	chr18	chr18	46704571	46821073	1	1	3	1	1	dup
ZNF44	ZNF442	chr19	chr19	12247450	12350298	0	0	2	1	1	dup
chr21_q11.2	chr21_q11.2	chr21	chr21	14027598	13970111	0	1	7	1	5	inv
TMEM184B	chr16_p13.3	chr22	chr16	38247769	1457271	0	1	5	1	1	tloc
chr22_q11.2	chr22_q11.22	chr22	chr22	22162679	22899617	0	0	4	1	1	del
SPECC1L:AD(	LARGE	chr22	chr22	24369321	33432266	0	1	3	1	1	inv

Supplemental Table S4. CNVs.
Shading: orange-CN gain; blue-CN loss; green-neutral LOH; yellow-genes with overexpression.

F48: Resection 1

Chrom	start	stop	major copy #	minor copy #	amplification	LOH	Genes
1	69090	955711	2	1	neutral	0	
1	957580	977011	3	0	gain	1	
1	977060	990361	4	0	gain	1	
1	1007200	1480351	2	1	neutral	0	
1	1500160	1718871	3	1	gain	0	
1	1720500	10479791	2	1	neutral	0	
1	10490580	10502481	4	2	gain	0	
1	10510280	11346151	2	1	neutral	0	
1	11561050	11596731	4	0	gain	1	
1	11708750	21904131	2	1	neutral	0	
1	21924220	21976281	4	2	gain	0	
1	21995750	28764911	2	1	neutral	0	
1	28784350	28823061	4	2	gain	0	
1	28856380	29563211	2	1	neutral	0	
1	29581790	29652161	4	2	gain	0	
1	31186430	32164207	2	1	neutral	0	
1	32165420	32230381	3	1	gain	0	
1	32256210	33116921	2	1	neutral	0	
1	33116960	33145743	4	0	gain	1	
1	33147390	36060251	2	1	neutral	0	
1	36065150	36107301	3	0	gain	1	
1	36181240	36282641	2	1	neutral	0	
1	36288460	36521341	3	0	gain	1	
1	36550530	43687931	2	1	neutral	0	
1	43688500	43766791	2	0	neutral	1	
1	43770530	44010801	2	1	neutral	0	
1	44019170	44118951	4	2	gain	0	
1	44121270	44438191	2	1	neutral	0	
1	44440720	44456111	4	2	gain	0	
1	44457550	46651201	2	1	neutral	0	
1	46654390	46743951	4	2	gain	0	
1	46744540	55465011	2	1	neutral	0	
1	55470700	55527214	4	2	gain	0	
1	55529050	57111111	2	1	neutral	0	
1	57140060	57173351	4	0	gain	1	
1	57185350	62905641	2	1	neutral	0	
1	62905690	63999869	3	1	gain	0	
1	63999870	71318541	2	1	neutral	0	
1	71327990	71512871	3	1	gain	0	
1	71530760	84544051	2	1	neutral	0	
1	84609950	84700971	4	2	gain	0	
1	84764240	92327081	2	1	neutral	0	
1	92428320	92479821	3	0	gain	1	
1	92495640	103348861	2	1	neutral	0	
1	103352370	104068851	4	2	gain	0	
1	104070320	149763051	2	1	neutral	0	
1	149783450	149859431	3	0	gain	1	
1	149871620	150981141	2	1	neutral	0	
1	150990290	151006691	4	0	gain	1	PRUNE1
1	151009210	153283091	2	1	neutral	0	

1	153283140	153412421	1	0	loss	1	
1	153430290	156873811	2	1	neutral	0	
1	156874540	156884585	3	1	gain	0	
1	156890600	167097841	2	1	neutral	0	
1	167190140	167385041	4	2	gain	0	POU2F1
1	167400920	170521561	2	1	neutral	0	
1	170633360	170705291	4	0	gain	1	
1	170914700	171310961	2	1	neutral	0	
1	171481230	171514771	4	2	gain	0	
1	171514820	171557621	4	0	gain	1	
1	171558510	171560981	3	0	gain	1	
1	171605070	176153821	2	1	neutral	0	
1	176175710	176176111	1	0	loss	1	
1	176525460	177133831	4	2	gain	0	
1	177199020	180135721	2	1	neutral	0	
1	180144460	180166471	4	2	gain	0	
1	180199670	198608451	2	1	neutral	0	
1	198661480	198725291	4	2	gain	0	
1	199996980	202119541	2	1	neutral	0	
1	202121670	202130681	4	2	gain	0	
1	202163120	202304881	2	1	neutral	0	
1	202317980	202544281	3	1	gain	0	
1	202549610	203672931	2	1	neutral	0	
1	203676140	203693111	4	0	gain	1	
1	203696530	205782881	2	1	neutral	0	
1	205797750	205817071	3	1	gain	0	
1	205819040	212459611	2	1	neutral	0	
1	212475150	212530601	3	1	gain	0	
1	212532030	214531371	2	1	neutral	0	
1	214537860	214638131	4	2	gain	0	PTPN14
1	214787100	226076761	2	1	neutral	0	
1	226108160	226111801	4	0	gain	1	
1	226125140	230415201	2	1	neutral	0	
1	230459170	230493041	3	0	gain	1	
1	230513250	233113961	2	1	neutral	0	
1	233120050	233431181	3	1	gain	0	
1	233463780	242162361	2	1	neutral	0	
1	242253160	242253411	4	0	gain	1	
1	242263970	244006881	2	1	neutral	0	
1	244013350	244218681	4	0	gain	1	
1	244528010	249208071	2	1	neutral	0	
1	249208640	249212561	3	0	gain	1	

2	41610	1643181	2	1	neutral	0
2	1647140	1748221	4	0	gain	1
2	1795640	20424621	2	1	neutral	0
2	20451350	20647731	4	0	gain	1
2	20818440	33623581	2	1	neutral	0
2	33740210	33787801	3	0	gain	1
2	33809850	43906061	2	1	neutral	0
2	43919660	44001281	4	2	gain	0
2	44003920	54087021	2	1	neutral	0
2	54093230	54197881	4	2	gain	0
2	54342840	55831171	2	1	neutral	0
2	55842590	55920951	4	0	gain	1
2	56094210	71160301	2	1	neutral	0

2	71163090	71212241	3	1	gain	0	
2	71215730	71215781	2	1	neutral	0	
2	71215830	86327291	2	1	neutral	0	
2	86332830	86364261	4	0	gain	1	
2	86364600	96262151	2	1	neutral	0	
2	96517530	96652941	3	1	gain	0	
2	96657260	97757401	2	1	neutral	0	
2	97779480	97827871	3	0	gain	1	
2	97829600	97899521	4	2	gain	0	
2	97899570	98201851	3	0	gain	1	
2	98205990	111334581	2	1	neutral	0	
2	111395540	111880981	3	1	gain	0	
2	111881030	112638391	3	0	gain	1	
2	112656320	113090061	2	1	neutral	0	
2	113127760	113174871	4	1	gain	0	
2	113180130	114390521	2	1	neutral	0	
2	114391730	114399701	4	2	gain	0	
2	114475330	119600821	2	1	neutral	0	
2	119603890	119752071	1	0	loss	1	
2	119914380	131113731	2	1	neutral	0	
2	131116440	131785621	4	2	gain	0	
2	131792560	136262051	2	1	neutral	0	
2	136289110	136481861	4	0	gain	1	R3HDM1
2	136499500	153501951	2	1	neutral	0	
2	153502000	153504411	3	0	gain	1	
2	153512820	153575531	4	0	gain	1	
2	153577050	155566321	3	0	gain	1	
2	155711240	158300511	2	1	neutral	0	
2	158390430	158454031	4	0	gain	1	
2	158485090	161132201	2	1	neutral	0	
2	161133820	161223881	3	1	gain	0	RBMS1
2	161263980	162092011	2	1	neutral	0	
2	162175340	162267871	4	0	gain	1	
2	162272930	170428521	2	1	neutral	0	
2	170460560	170492711	3	0	gain	1	
2	170492930	179170971	2	1	neutral	0	
2	179184970	179368591	4	2	gain	0	
2	179391740	190541721	2	1	neutral	0	
2	190554260	190557901	4	0	gain	1	
2	190559710	190927251	3	0	gain	1	
2	190927300	192821031	2	1	neutral	0	
2	192821080	198636631	3	1	gain	0	
2	198636680	203329691	2	1	neutral	0	
2	203332250	203424661	4	2	gain	0	
2	203499910	209116251	2	1	neutral	0	
2	209136250	209358341	4	2	gain	0	
2	210517900	215279221	2	1	neutral	0	
2	215279270	216177321	3	1	gain	0	
2	216182880	216214261	4	0	gain	1	
2	216214310	216214361	4	0	gain	1	
2	216226280	233273261	2	1	neutral	0	
2	233273420	233274151	5	0	gain	1	
2	233274290	236617881	2	1	neutral	0	
2	236626200	237032761	4	2	gain	0	
2	237074560	242448101	2	1	neutral	0	
2	242498900	242511821	3	0	gain	1	

2	242524020	242841471	2	1	neutral	0	
3	361460	8809704	3	1	gain	0	
3	8923040	9005061	6	0	gain	1	RAD18
3	9027210	10362701	3	1	gain	0	
3	10370500	10491221	5	2	gain	0	ATP2B2
3	10857950	11302181	3	1	gain	0	
3	11340170	11406191	5	0	gain	1	ATG7
3	11421450	11685361	4	0	gain	1	
3	11712740	20025301	3	1	gain	0	incl RAF1
3	20026880	36527731	2	1	neutral	0	
3	36534650	46666671	1	0	loss	1	
3	46712480	46730921	2	0	neutral	1	
3	46742980	46751171	1	0	loss	1	
3	46753740	46759121	2	0	neutral	1	
3	46759210	47385401	1	0	loss	1	
3	47422390	47454651	2	0	neutral	1	
3	47455350	48732851	1	0	loss	1	
3	48752750	48885021	2	1	neutral	0	
3	48895150	49066741	1	0	loss	1	
3	49067890	49142151	2	0	neutral	1	
3	49145850	49570631	1	0	loss	1	
3	49662410	49701984	2	0	neutral	1	
3	49702240	49967301	1	0	loss	1	
3	50000080	50155871	2	1	neutral	0	
3	50197060	50365441	1	0	loss	1	
3	50365490	50375581	2	0	neutral	1	
3	50375630	51426491	1	0	loss	1	
3	51428830	51697431	2	1	neutral	0	
3	51708320	51995765	1	0	loss	1	
3	52003450	52015011	2	0	neutral	1	
3	52018080	52434441	1	0	loss	1	
3	52436300	52448021	2	0	neutral	1	
3	52448520	54156841	1	0	loss	1	
3	54157540	56763641	2	1	neutral	0	
3	56766270	57069171	4	2	gain	0	
3	57107730	63953621	2	1	neutral	0	
3	63965590	64004381	3	0	gain	1	
3	64004500	64009511	5	2	gain	0	PSMD6
3	64084730	64673321	3	1	gain	0	
3	65342060	74420501	2	1	neutral	0	
3	74420540	93783381	3	1	gain	0	
3	93802950	111368651	2	1	neutral	0	
3	111394100	111718421	3	1	gain	0	
3	111719620	113500001	2	1	neutral	0	
3	113503070	113524351	4	0	gain	1	ATP6V1A
3	113528190	133114811	2	1	neutral	0	
3	133118930	133193813	4	0	gain	1	BFSP2
3	133292890	134977951	2	1	neutral	0	
3	135720340	135863991	3	1	gain	0	
3	135869990	141231111	2	1	neutral	0	
3	141235170	141328871	4	2	gain	0	RASA2
3	141331130	142166771	2	1	neutral	0	
3	142168260	142297531	4	2	gain	0	ATR
3	142383080	146254331	2	1	neutral	0	
3	146303860	146323981	4	0	gain	1	

3	147108720	149470221	2	1	neutral	0	
3	149479270	149508671	4	2	gain	0	
3	149510070	158409231	2	1	neutral	0	
3	158415470	158450431	4	2	gain	0	
3	158519800	195505311	2	1	neutral	0	
3	195505660	195518361	3	1	gain	0	
3	195538610	197896711	2	1	neutral	0	
4	53382	6304191	2	1	neutral	0	
4	6325030	6349631	3	0	gain	1	
4	6349680	6415731	4	2	gain	0	
4	6422680	7765511	2	1	neutral	0	
4	7770570	8160411	4	2	gain	0	
4	8206930	31144451	2	1	neutral	0	
4	36069530	36340791	3	0	gain	1	
4	36345130	56348951	2	1	neutral	0	
4	56422730	56447041	4	0	gain	1	PDCL2
4	56448290	56458271	5	0	gain	1	PDCL2
4	56466690	87556511	1	0	loss	1	
4	87593520	87735661	2	1	neutral	0	
4	87744840	190862221	1	0	loss	1	
4	190864360	190947541	2	1	neutral	0	
4	190947590	190947591	1	0	loss	1	
5	140430	182421	3	1	gain	0	
5	191660	22505431	2	1	neutral	0	
5	23509150	23524601	4	0	gain	1	
5	23526350	33467771	2	1	neutral	0	
5	33527300	33588911	4	2	gain	0	
5	33596040	34829891	2	1	neutral	0	
5	34830800	34830851	4	0	gain	1	
5	34839740	45645691	2	1	neutral	0	
5	45695770	64267631	1	0	loss	1	
5	64272970	64890451	2	0	neutral	1	
5	64892250	180687511	1	0	loss	1	
6	292540	292551	1	0	loss	1	
6	304630	3851021	2	1	neutral	0	
6	4021660	4060841	4	2	gain	0	
6	4068930	7609101	2	1	neutral	0	
6	7727190	7880571	3	0	gain	1	
6	7883380	12296291	2	1	neutral	0	
6	12718980	13230421	4	2	gain	0	
6	13273100	13618341	2	1	neutral	0	
6	13620440	13697121	3	0	gain	1	
6	13711170	26108283	2	1	neutral	0	
6	26123760	26124761	4	0	gain	1	
6	26124810	26197421	3	1	gain	0	
6	26197470	26205181	4	2	gain	0	
6	26216490	26638781	2	1	neutral	0	
6	27100100	27101151	3	0	gain	1	
6	27101200	27783181	4	2	gain	0	
6	27791910	27806031	4	0	gain	1	
6	27806080	27860841	4	2	gain	0	
6	27860890	27861591	4	0	gain	1	
6	27879030	31584671	2	1	neutral	0	

6	31590570	31591661	3	0	gain	1	
6	31592040	31598921	4	0	gain	1	
6	31600760	31634651	3	0	gain	1	
6	31635650	43042401	2	1	neutral	0	
6	43044230	43044661	3	0	gain	1	
6	43044710	43126701	4	2	gain	0	
6	43127530	43144391	3	0	gain	1	
6	43146040	43337061	2	1	neutral	0	
6	43395720	43419671	4	2	gain	0	
6	43419680	65612061	2	1	neutral	0	
6	65612110	66205061	3	1	gain	0	
6	69348570	70092821	4	2	gain	0	
6	70098600	70428901	3	1	gain	0	
6	70428950	88224181	2	1	neutral	0	
6	88224680	88229401	4	0	gain	1	
6	88229900	109816641	2	1	neutral	0	
6	109818670	109996941	3	1	gain	0	
6	110012640	128134686	2	1	neutral	0	
6	128135070	128294961	3	0	gain	1	
6	128297810	128718761	4	2	gain	0	
6	128718810	128841461	3	0	gain	1	
6	129204390	155776931	2	1	neutral	0	
6	157098980	157739921	4	2	gain	0	
6	157743740	161537901	2	1	neutral	0	
6	161557580	163483341	3	1	gain	0	
6	163510290	163989461	5	2	gain	0	QKI
6	165693510	170893641	2	1	neutral	0	
7	193200	618691	2	1	neutral	0	
7	618900	751121	3	0	gain	1	
7	766360	5553321	2	1	neutral	0	
7	5567380	5569271	3	0	gain	1	
7	5632570	6744801	2	1	neutral	0	
7	6797310	6845661	5	0	gain	1	RSPH10B2
7	6851590	20449601	2	1	neutral	0	
7	20662910	20795241	4	2	gain	0	
7	20823910	35840871	2	1	neutral	0	
7	35840886	36552941	3	1	gain	0	
7	36552980	43601521	2	1	neutral	0	
7	43622850	43623041	1	0	loss	1	
7	43635500	44268451	2	1	neutral	0	
7	44268500	44432051	1	0	loss	1	
7	44444040	47698811	2	1	neutral	0	
7	47814740	47988031	3	1	gain	0	
7	48004960	48285201	2	1	neutral	0	
7	48285460	48685071	4	2	gain	0	
7	49815040	72396161	2	1	neutral	0	
7	72396800	72430431	3	0	gain	1	
7	72430560	74203011	2	1	neutral	0	
7	74234500	74456771	5	2	gain	0	GTF2IRD2
7	74466820	75131261	3	1	gain	0	
7	75167500	94293841	2	1	neutral	0	
7	94539430	94876831	3	0	gain	1	
7	94879340	94918001	6	3	gain	0	PPP1R9A
7	94919410	98805081	2	1	neutral	0	
7	98930980	99160091	3	0	gain	1	

7	99161490	101962071	2	1	neutral	0	
7	101962120	101962171	1	0	loss	1	
7	101988730	121023021	2	1	neutral	0	
7	121513560	121701241	3	1	gain	0	
7	121716550	124430651	2	1	neutral	0	
7	124464010	124537231	3	0	gain	1	
7	126079180	140302191	2	1	neutral	0	
7	140373130	140394871	3	0	gain	1	
7	140395580	141768501	2	1	neutral	0	
7	141770800	141794451	1	0	loss	1	
7	141794550	150721461	2	1	neutral	0	
7	150725560	150747671	3	0	gain	1	
7	150747850	152457011	2	1	neutral	0	
7	152480280	152551601	4	0	gain	1	
7	152841290	157208761	2	1	neutral	0	
7	157333410	158334461	3	1	gain	0	
7	158380250	158937421	2	1	neutral	0	
8	190900	7698711	2	1	neutral	0	
8	7717870	7718471	4	0	gain	1	
8	7721030	18413821	2	1	neutral	0	
8	10388910	17580031	2	1	neutral	0	
8	18430040	18793551	4	2	gain	0	
8	18793600	18793651	3	0	gain	1	
8	18871080	25902351	2	1	neutral	0	
8	26149340	26267921	3	0	gain	1	
8	26365180	37611591	2	1	neutral	0	
8	37620076	37633491	3	0	gain	1	
8	37635490	37635591	2	1	neutral	0	
8	37654790	37690721	1	0	loss	1	
8	37691210	38959441	2	1	neutral	0	
8	38961130	39142312	4	0	gain	1	
8	39442150	48650381	2	1	neutral	0	
8	48686740	48872681	3	0	gain	1	
8	48873710	53580731	2	1	neutral	0	
8	53586410	53596551	5	2	gain	0	
8	53597960	53598011	4	0	gain	1	
8	53852470	59750841	2	1	neutral	0	
8	59764090	62412021	3	0	gain	1	
8	62412070	62626921	4	2	gain	0	
8	63161640	68111331	2	1	neutral	0	
8	68112630	68255751	3	0	gain	1	
8	68255800	95272701	2	1	neutral	0	
8	95384400	95501081	3	0	gain	1	
8	95502170	97285581	2	1	neutral	0	
8	97296340	97345791	4	0	gain	1	
8	97506500	110352781	2	1	neutral	0	
8	110355640	110401311	4	2	gain	0	
8	110401360	110476994	3	0	gain	1	
8	110477470	117862961	3	1	gain	0	
8	117863000	141525331	2	1	neutral	0	
8	141542150	141669771	3	1	gain	0	PTK2/FAK
8	141675020	141727801	4	0	gain	1	PTK2/FAK
8	141745350	141931221	4	2	gain	0	
8	141935760	141935811	4	0	gain	1	
8	142138810	142432441	2	1	neutral	0	

8	142435150	142441131	4	0	gain	1	
8	142444060	143436051	2	1	neutral	0	
8	143545560	143695631	4	0	gain	1	
8	143740270	144897841	2	1	neutral	0	
8	144898690	144906541	4	0	gain	1	
8	144911160	145597791	2	1	neutral	0	
8	145599160	145618281	3	1	gain	0	
8	145618540	146279511	2	1	neutral	0	
9	14810	135021	5	2	gain	0	WASHC1; FOXD4
9	146110	33473831	2	1	neutral	0	
9	33524490	33799171	4	2	gain	0	
9	33817760	38424221	2	1	neutral	0	
9	38543250	38620281	3	1	gain	0	
9	39073890	42368611	2	1	neutral	0	
9	42406570	43133631	5	0	gain	1	ANKRD20A2
9	43624660	72755161	2	1	neutral	0	
9	72758470	74840651	1	0	loss	1	
9	74840690	98011551	2	1	neutral	0	
9	98209190	98279091	5	2	gain	0	PTCH1
9	98638290	112082671	2	1	neutral	0	
9	112082720	112141891	3	0	gain	1	
9	112143940	112219491	4	0	gain	1	
9	112219580	123555221	2	1	neutral	0	
9	123580190	123595691	4	2	gain	0	
9	123605020	125719481	2	1	neutral	0	
9	125746770	125852661	4	0	gain	1	
9	125860020	127905711	2	1	neutral	0	
9	127911960	127996221	4	0	gain	1	
9	127998870	131873311	2	1	neutral	0	
9	131873600	131899971	4	0	gain	1	
9	131904730	132404471	2	1	neutral	0	
9	132481510	132510991	4	0	gain	1	
9	132515170	133309041	2	1	neutral	0	
9	133320560	133376401	4	0	gain	1	
9	133455070	133512401	2	1	neutral	0	
9	133540040	133557041	5	2	gain	0	PRDM12
9	133569180	133972221	2	1	neutral	0	
9	133981580	134001121	4	2	gain	0	
9	134002910	134371231	2	1	neutral	0	
9	134379610	134379711	3	0	gain	1	
9	134381500	134398471	4	2	gain	0	
9	134400430	134814821	3	1	gain	0	
9	134889730	135804301	2	1	neutral	0	
9	135862070	135946001	2	0	neutral	1	
9	135946380	136899931	2	1	neutral	0	
9	136901150	136918691	4	2	gain	0	
9	137005000	139379401	2	1	neutral	0	
9	139390520	139438521	3	1	gain	0	
9	139440170	139702331	2	1	neutral	0	
9	139702650	139734931	4	2	gain	0	RABL6
9	139743890	140347561	2	1	neutral	0	
9	140347610	140353491	3	0	gain	1	
9	140353540	140356731	3	1	gain	0	
9	140356780	140374921	4	0	gain	1	
9	140375420	140441811	3	0	gain	1	

9	140442040	141111751	2	1	neutral	0	
10	93000	454921	3	1	gain	0	
10	459860	486911	20	0	gain	1	DIP2C 3'
10	518380	1170951	3	0	gain	1	
10	1171000	5904641	3	1	gain	0	
10	5920050	5937121	20	0	gain	1	ANKRD16
10	5944990	17272701	3	1	gain	0	
10	17275590	17279261	20	0	gain	1	VIM
10	17362880	22608971	3	1	gain	0	
10	22615360	22618451	20	0	gain	1	BMI1
10	22634540	23235181	3	0	gain	1	
10	23244740	23270621	20	0	gain	1	ARMC3 5'
10	23287080	24835251	3	1	gain	0	
10	24873340	25305041	5	2	gain	0	ARHGAP21; PRTFDC1
10	25312160	27031481	3	1	gain	0	
10	27035270	27035371	4	0	gain	1	
10	27037500	27066151	20	0	gain	1	ABI1
10	27112070	27389221	4	0	gain	1	
10	27400910	27470461	3	0	gain	1	
10	27470500	27529601	4	1	gain	0	
10	27687225	33190501	3	1	gain	0	
10	33190550	33190551	4	0	gain	1	
10	33195990	90774181	2	1	neutral	0	incl PTEN
10	90966230	102988551	1	0	loss	1	
10	103113940	103310611	2	0	neutral	1	
10	103339210	118117441	1	0	loss	1	
10	118137940	118236661	2	0	neutral	1	
10	118305610	120925121	1	0	loss	1	
10	120927990	120938301	2	0	neutral	1	
10	120967430	121411341	1	0	loss	1	
10	121429370	121510641	2	1	neutral	0	
10	121541150	122348971	1	0	loss	1	
10	122610940	122646301	20	0	gain	1	WDR11
10	122646340	123353321	20	0	gain	1	FGFR2
10	123503120	123686751	20	0	gain	1	ATE11
10	123687360	123954661	20	0	gain	1	NSMCE4A; TACC2 5'
10	123969920	135440221	1	0	loss	1	
11	193100	1330721	1	0	loss	1	
11	1431680	1501681	2	0	neutral	1	
11	1501960	47436891	1	0	loss	1	
11	47440390	47469671	2	1	neutral	0	
11	47470330	47869971	1	0	loss	1	
11	48131610	48188911	2	0	neutral	1	
11	48238370	60906261	2	1	neutral	0	
11	60971040	61018711	4	0	gain	1	
11	61026150	64084971	2	1	neutral	0	
11	64085450	64088371	4	0	gain	1	
11	64088480	64453411	2	1	neutral	0	
11	64455840	64514801	3	0	gain	1	
11	64517850	64527331	4	0	gain	1	
11	64532960	65380991	2	1	neutral	0	
11	65383790	65404441	3	0	gain	1	
11	65408400	67141571	2	1	neutral	0	
11	67159520	67168361	3	0	gain	1	

11	67168540	69951891	2	1	neutral	0	
11	69954460	70228251	4	2	gain	0	
11	70253410	72396721	2	1	neutral	0	
11	72397090	72438171	3	1	gain	0	
11	72465950	73136091	2	1	neutral	0	
11	73141740	73638381	3	1	gain	0	
11	73662120	73850851	2	1	neutral	0	
11	73872450	74109201	4	2	gain	0	
11	74168300	82444771	2	1	neutral	0	
11	82535950	82611431	5	2	gain	0	PRCP
11	82625780	87032351	2	1	neutral	0	
11	87847160	87908551	4	2	gain	0	
11	88027180	108068131	2	1	neutral	0	
11	108093230	108236211	4	2	gain	0	
11	108253480	110453151	2	1	neutral	0	
11	110454280	110582731	3	0	gain	1	
11	110582850	111612831	2	1	neutral	0	
11	111613250	111635621	4	0	gain	1	
11	111636020	114271091	2	1	neutral	0	
11	114271400	114278521	3	0	gain	1	
11	114310250	120099831	2	1	neutral	0	
11	120107390	120355201	3	0	gain	1	
11	120355780	125221261	2	1	neutral	0	
11	125237750	125301271	4	0	gain	1	
11	125315990	129763011	2	1	neutral	0	
11	129772210	129827791	4	2	gain	0	
11	129830790	134257701	2	1	neutral	0	
12	176050	6346931	2	1	neutral	0	
12	6346980	6435651	1	0	loss	1	
12	6435700	9265131	2	1	neutral	0	
12	9265960	9712761	3	1	gain	0	
12	9747870	10539601	2	1	neutral	0	
12	10541370	10569361	4	0	gain	1	
12	10570950	10588651	6	2	gain	0	KLRC3
12	10588700	10588751	5	0	gain	1	KLRC2
12	10598900	10607181	3	0	gain	1	
12	10758880	15264331	2	1	neutral	0	
12	15274000	15739981	4	2	gain	0	
12	15742390	23893951	2	1	neutral	0	
12	23908580	25055981	4	0	gain	1	
12	25101860	25314051	2	1	neutral	0	
12	25314100	25801481	2	1	neutral	0	
12	26208280	26232001	4	2	gain	0	
12	26274940	29586741	2	1	neutral	0	
12	29596300	29649591	4	2	gain	0	
12	29650560	31256941	2	1	neutral	0	
12	31267690	31285231	5	2	gain	0	OVOS2
12	31286050	31359081	6	2	gain	0	OVOS2
12	31435650	42717821	2	1	neutral	0	
12	42717870	45004731	3	1	gain	0	
12	45059270	45797231	1	0	loss	1	
12	45797280	46298821	2	0	neutral	1	
12	46315830	49251981	1	0	loss	1	
12	49254780	49393061	2	1	neutral	0	
12	49396690	49412801	4	2	gain	0	

12	49415560	49523131	2	1	neutral	0	
12	49523290	49582701	4	2	gain	0	
12	49621740	50369361	2	1	neutral	0	
12	50384050	50474481	4	2	gain	0	
12	50474890	53346561	2	1	neutral	0	
12	53605460	53623801	4	0	gain	1	
12	53645520	53901251	2	1	neutral	0	
12	53910920	54069941	4	2	gain	0	
12	54105730	56881871	2	1	neutral	0	
12	56915810	57146081	4	2	gain	0	
12	57167640	70915301	2	1	neutral	0	
12	70918240	70928361	3	0	gain	1	
12	70928410	71003581	4	2	gain	0	
12	71003630	71314121	3	1	gain	0	
12	71314160	80172371	2	1	neutral	0	
12	80173100	82871111	3	1	gain	0	
12	82872710	93101491	2	1	neutral	0	
12	93115250	93163971	4	0	gain	1	
12	93169790	108082281	2	1	neutral	0	
12	108082400	108150711	4	2	gain	0	
12	108154290	111886031	2	1	neutral	0	
12	111886080	111891641	3	0	gain	1	
12	111893840	112204841	3	1	gain	0	
12	112204890	112856931	2	1	neutral	0	
12	112884070	112942541	4	0	gain	1	
12	113266130	122460051	2	1	neutral	0	
12	122468610	122497051	5	2	gain	0	BCL7A
12	122516690	123320121	2	1	neutral	0	
12	123320170	123880901	3	1	gain	0	
12	123880950	133810901	2	1	neutral	0	
13	19748010	25672231	2	1	neutral	0	
13	25743690	25745851	4	0	gain	1	
13	25823370	28644671	2	1	neutral	0	
13	28644720	28674651	1	0	loss	1	
13	28712900	32885411	2	1	neutral	0	
13	32885910	32890641	3	0	gain	1	BRCA2
13	32893210	32972791	4	2	gain	0	BRCA2
13	32976020	38153251	2	1	neutral	0	
13	38153370	38172851	4	0	gain	1	
13	38211040	115091751	2	1	neutral	0	
14	19377600	20404737	3	1	gain	0	
14	20443680	30047531	2	1	neutral	0	
14	30066700	30194851	4	2	gain	0	
14	30396460	35255391	2	1	neutral	0	
14	35261980	35331401	4	2	gain	0	
14	35331450	35331501	4	0	gain	1	
14	35343700	35554951	2	1	neutral	0	
14	35557160	35579131	5	0	gain	1	PPP2R3C (PP2A)
14	35579730	45542751	2	1	neutral	0	
14	45564450	45584111	4	0	gain	1	
14	45585400	51338991	2	1	neutral	0	
14	51344670	51372261	3	1	gain	0	
14	51374990	51411081	5	2	gain	0	PYGL
14	51444010	51716461	3	1	gain	0	

14	51716467	53162191	2	1	neutral	0	
14	53173900	53185751	3	0	gain	1	
14	53187580	60337311	2	1	neutral	0	
14	60574360	60600931	4	2	gain	0	
14	60611690	71216791	2	1	neutral	0	
14	71227720	71374711	1	0	loss	1	
14	71413640	73465021	2	1	neutral	0	
14	73490740	73739361	3	0	gain	1	
14	73743290	77737251	2	1	neutral	0	
14	77743720	77786981	5	2	gain	0	POMT2
14	77787520	90722942	2	1	neutral	0	
14	90725510	90736691	4	2	gain	0	
14	90738620	105996131	2	1	neutral	0	
15	20739500	31779451	2	1	neutral	0	
15	31779660	31947441	4	2	gain	0	
15	32322800	38773801	2	1	neutral	0	
15	38776460	38852181	4	0	gain	1	
15	38856800	40398251	2	1	neutral	0	
15	40453430	40505641	4	2	gain	0	
15	40509700	42264661	2	1	neutral	0	
15	42275960	42447771	3	0	gain	1	
15	42448640	43827381	2	1	neutral	0	
15	43828340	43852761	4	0	gain	1	
15	43873200	55485021	2	1	neutral	0	
15	55489010	55522651	3	0	gain	1	
15	55526980	63414371	2	1	neutral	0	
15	63414420	65808021	2	1	neutral	0	
15	65822970	65868691	4	0	gain	1	
15	65871750	66048781	2	1	neutral	0	
15	66161790	66180141	4	0	gain	1	
15	66190280	72700191	2	1	neutral	0	
15	72767220	72767321	1	0	loss	1	
15	72789140	75555721	2	1	neutral	0	
15	75556140	75562521	4	0	gain	1	
15	75580600	75761311	2	1	neutral	0	
15	75762140	75815571	3	0	gain	1	
15	75816550	77287151	2	1	neutral	0	
15	77287470	77329441	3	0	gain	1	
15	77329490	77628621	3	1	gain	0	
15	77629430	77697101	4	2	gain	0	
15	77709640	78933471	2	1	neutral	0	
15	79051800	79092881	4	2	gain	0	
15	79103470	80696881	2	1	neutral	0	
15	80733580	81046701	3	0	gain	1	
15	81166220	91185371	2	1	neutral	0	
15	91290620	91358481	3	0	gain	1	
15	91418970	91504991	2	1	neutral	0	
15	91510360	91528031	4	2	gain	0	
15	91537640	102462851	2	1	neutral	0	
16	97430	640401	2	1	neutral	0	
16	667210	677601	4	0	gain	1	
16	681260	16317761	2	1	neutral	0	
16	16330760	16484731	4	0	gain	1	
16	17202560	20364051	2	1	neutral	0	

16	20370640	20376851	4	2	gain	0	
16	20380820	20696721	3	1	gain	0	
16	20702320	24557541	2	1	neutral	0	
16	24560270	24578821	4	0	gain	1	
16	24579120	28665811	2	1	neutral	0	
16	28668070	28782691	4	0	gain	1	
16	28834580	28891411	2	1	neutral	0	
16	28892240	28913701	4	0	gain	1	
16	28913900	48119611	2	1	neutral	0	
16	48120660	48265991	3	1	gain	0	
16	48278300	53499501	2	1	neutral	0	
16	53500970	53524201	4	2	gain	0	
16	53526330	57660651	2	1	neutral	0	
16	57660870	57679271	3	1	gain	0	
16	57680320	71971301	2	1	neutral	0	
16	71976470	72033841	4	2	gain	0	
16	72042670	75298491	2	1	neutral	0	
16	75299730	75299881	1	0	loss	1	
16	75300620	84360541	2	1	neutral	0	
16	84402230	84495411	3	1	gain	0	
16	84495600	90094101	2	1	neutral	0	
16	90095400	90095741	5	0	gain	1	GAS8 segm
16	90097590	90142251	3	1	gain	0	
16	90142300	90142301	4	0	gain	1	
17	6010	1554811	2	1	neutral	0	
17	1554950	1587821	3	0	gain	1	
17	1611070	1946391	2	1	neutral	0	
17	1959610	1989171	2	0	neutral	1	
17	1990370	2866751	2	1	neutral	0	
17	2867150	2911411	4	0	gain	1	
17	2921360	3819491	2	1	neutral	0	
17	3828680	3857001	3	0	gain	1	
17	3910190	5138031	2	0	neutral	1	
17	5138530	5405141	2	1	neutral	0	
17	5405190	7637941	2	0	neutral	1	incl TP53
17	7637990	7726861	2	1	neutral	0	
17	7726910	9596501	2	0	neutral	1	
17	9596540	9729481	2	1	neutral	0	
17	9729530	9885121	1	0	loss	1	
17	9885170	10247371	2	1	neutral	0	
17	10247420	10626591	2	0	neutral	1	
17	10626640	16089921	2	1	neutral	0	
17	16089970	16640961	2	0	neutral	1	
17	16664740	17088141	1	0	loss	1	
17	17088190	17726891	2	1	neutral	0	
17	17726930	19186441	2	0	neutral	1	
17	19186940	31323991	2	1	neutral	0	
17	31324470	31324781	1	0	loss	1	
17	31340990	32646571	2	1	neutral	0	
17	32647290	32690161	1	0	loss	1	
17	32905890	38132181	2	1	neutral	0	
17	38132230	38573941	3	1	gain	0	
17	38574030	44833181	2	1	neutral	0	
17	44845690	45201261	1	0	loss	1	
17	45201310	45266521	2	1	neutral	0	

17	45266530	46629831	1	0	loss	1	
17	46654090	47925361	2	1	neutral	0	
17	48046840	54555001	3	1	gain	0	
17	54558030	54559871	6	0	gain	1	ANKFN1 3'
17	54576270	56084461	3	1	gain	0	
17	56164460	56358111	2	1	neutral	0	
17	56379630	56770151	3	1	gain	0	
17	56772290	57050251	6	0	gain	1	RAD51C; PPM1E
17	57057340	57278981	3	1	gain	0	
17	57287420	58469291	2	1	neutral	0	
17	58498750	58603261	3	0	gain	1	
17	58677780	58756871	5	0	gain	1	PPM1D
17	58761350	63531801	3	1	gain	0	
17	63532440	63554592	6	2	gain	0	AXIN2
17	63632090	64179411	3	1	gain	0	
17	64208250	64801251	5	0	gain	1	APOH PRKCA
17	64873450	65241621	4	2	gain	0	
17	65336960	65353691	6	0	gain	1	PSMD12
17	65362530	65905851	4	0	gain	1	
17	65907000	65942031	6	0	gain	1	BPTF
17	65943850	66265441	5	2	gain	0	KPNA2; AMZ2
17	66265480	79881061	3	1	gain	0	
17	79890760	79895081	6	0	gain	1	PYCR1
17	79898700	80606201	3	1	gain	0	
17	80615740	80620342	5	2	gain	0	RAB40B
17	80622380	81052251	3	1	gain	0	
17	81052300	81052301	3	0	gain	1	
18	158700	158711	1	0	loss	1	
18	163310	7567871	2	1	neutral	0	
18	7774150	8387221	4	2	gain	0	PTPRM
18	8394480	9517301	2	1	neutral	0	
18	9522160	11610611	4	2	gain	0	
18	11689570	31708851	2	1	neutral	0	
18	31709840	31803211	4	0	gain	1	
18	32335940	52989401	2	1	neutral	0	
18	53017590	53071161	4	0	gain	1	
18	53071210	53254331	3	0	gain	1	
18	53255270	78005211	2	1	neutral	0	
19	281390	288171	3	1	gain	0	
19	290960	652291	2	1	neutral	0	
19	652830	681701	1	0	loss	1	
19	685720	821541	2	1	neutral	0	
19	827850	841061	4	2	gain	0	
19	843460	1241951	2	1	neutral	0	
19	1242460	1272431	4	2	gain	0	
19	1275550	5208041	2	1	neutral	0	
19	5208250	5286061	4	2	gain	0	
19	5286110	5286161	3	0	gain	1	
19	5455510	7267861	2	1	neutral	0	
19	7268430	7554391	3	1	gain	0	
19	7565710	8642191	2	1	neutral	0	
19	8645780	8809051	4	2	gain	0	
19	8841390	10207161	2	1	neutral	0	
19	10213230	10222411	3	0	gain	1	

19	10224310	14201201	2	1	neutral	0	
19	14203930	14225971	4	2	gain	0	
19	14228320	17389851	2	1	neutral	0	
19	17392570	17412171	4	2	gain	0	
19	17416710	17434681	2	1	neutral	0	
19	17435530	17445471	4	2	gain	0	
19	17445820	17515231	3	1	gain	0	
19	17516100	41188851	2	1	neutral	0	
19	41190330	41221471	4	2	gain	0	
19	41223040	42417061	2	1	neutral	0	
19	42461000	42498221	3	0	gain	1	
19	42507490	43098971	2	1	neutral	0	
19	43228140	43233481	3	0	gain	1	
19	43233520	43376121	3	1	gain	0	
19	43376170	43773571	4	2	gain	0	
19	43857870	44079601	2	1	neutral	0	
19	44081280	44086121	4	2	gain	0	
19	44096180	46195251	2	1	neutral	0	
19	46195970	46206271	4	0	gain	1	
19	46214950	46842841	2	1	neutral	0	
19	46850360	46894431	3	0	gain	1	
19	46914450	47177981	2	1	neutral	0	
19	47178290	47217571	3	0	gain	1	
19	47219390	49222281	2	1	neutral	0	
19	49224060	49243511	4	2	gain	0	
19	49243780	49652521	2	1	neutral	0	
19	49652570	49949911	1	0	loss	1	
19	49950280	50063931	2	1	neutral	0	
19	50086480	50128821	3	0	gain	1	
19	50138840	50177011	2	1	neutral	0	
19	50180540	50193721	4	2	gain	0	
19	50195510	50340161	2	1	neutral	0	
19	50354140	50363571	3	0	gain	1	
19	50364530	52671361	2	1	neutral	0	
19	52693350	52729231	4	2	gain	0	
19	52772870	54782411	2	1	neutral	0	
19	54782670	54782821	1	0	loss	1	
19	54782870	54782921	1	0	loss	1	
19	54783200	55690531	2	1	neutral	0	
19	55693130	55740101	3	1	gain	0	
19	55741895	55758451	4	2	gain	0	
19	55773740	59083971	2	1	neutral	0	
20	68350	1559331	2	1	neutral	0	
20	1578310	1592351	20	0	gain	1	SIRPB1
20	1600520	2847181	2	1	neutral	0	
20	2903910	3018761	4	2	gain	0	PTPRA
20	3025060	3388441	2	1	neutral	0	
20	3451720	3624901	4	2	gain	0	
20	3627370	20350441	2	1	neutral	0	
20	20373750	20693121	4	0	gain	1	RALGAPA2
20	21106680	25206191	2	1	neutral	0	
20	25228820	25277151	4	0	gain	1	
20	25280840	32268191	2	1	neutral	0	
20	32295520	32308001	3	0	gain	1	
20	32319830	33150331	2	1	neutral	0	

20	33162910	33245041	4	0	gain	1	
20	33264760	37063991	2	1	neutral	0	
20	37117080	37464811	4	2	gain	0	
20	37518240	37547301	4	0	gain	1	
20	37555000	40162191	2	1	neutral	0	
20	40179950	40747051	3	1	gain	0	
20	40747100	41400181	4	2	gain	0	
20	41408860	42089701	3	1	gain	0	
20	42142130	46331371	2	1	neutral	0	
20	46365450	47244501	3	0	gain	1	
20	47245990	47364401	4	2	gain	0	
20	47444180	55213041	2	1	neutral	0	
20	55746020	55841161	4	0	gain	1	
20	55904930	56075251	3	1	gain	0	
20	56078500	62904931	2	1	neutral	0	
21	10906910	15013871	2	1	neutral	0	
21	15481280	45527311	1	0	loss	1	
21	45528870	45554031	2	1	neutral	0	
21	45555950	48063551	1	0	loss	1	
21	48064220	48084261	2	1	neutral	0	
22	16266930	18209951	2	1	neutral	0	
22	18218350	18256431	4	0	gain	1	
22	18273500	19951791	2	1	neutral	0	
22	19954500	19978311	4	2	gain	0	
22	20024330	22221711	2	1	neutral	0	
22	22277470	22294011	4	2	gain	0	
22	22300220	24323191	2	1	neutral	0	
22	24373140	24384221	4	0	gain	1	
22	24431965	25024801	2	1	neutral	0	
22	25115440	25202311	4	0	gain	1	
22	25202410	41636891	2	1	neutral	0	
22	41642610	41652771	4	2	gain	0	RANGAP1
22	41652820	41652821	5	0	gain	1	RANGAP1
22	41653960	41677611	4	0	gain	1	RANGAP1
22	41681550	46372871	2	1	neutral	0	
22	46449630	46631271	4	2	gain	0	
22	46640990	51183581	2	1	neutral	0	
22	51207210	51220721	6	0	gain	1	RABL2B
X	295060	2799251	2	1	neutral	0	
X	2825320	2951361	3	1	gain	0	
X	2951410	3030571	4	2	gain	0	
X	3227760	16863211	2	1	neutral	0	
X	16863950	16887971	3	1	gain	0	
X	16888160	18800511	2	1	neutral	0	
X	18802020	18845601	4	0	gain	1	
X	18911610	37263021	2	1	neutral	0	
X	37265470	37312841	4	0	gain	1	
X	37431130	39937191	2	1	neutral	0	
X	40440320	40464971	4	0	gain	1	
X	40482820	47001821	2	1	neutral	0	
X	47001990	47045981	4	2	gain	0	
X	47056480	55054231	2	1	neutral	0	
X	55102480	55117871	4	0	gain	1	PAGE2B

X	55170220	69489231	2	1	neutral	0
X	69489520	69500951	4	2	gain	0
X	69501530	76711971	2	1	neutral	0
X	76763830	77271351	3	0	gain	1
X	77275740	77296191	4	0	gain	1
X	77298090	77302041	3	0	gain	1
X	77359840	119010491	2	1	neutral	0
X	119037190	119053921	3	0	gain	1
X	119054470	129537821	2	1	neutral	0
X	129543230	129546791	3	0	gain	1
X	129759290	150790061	2	1	neutral	0
X	150791410	150844571	3	1	gain	0
X	150867290	152752241	2	1	neutral	0
X	152770090	152845741	3	0	gain	1
X	152853830	153649301	2	1	neutral	0
X	153657040	153664231	3	0	gain	1
X	153665600	155004401	2	1	neutral	0
Y	21154300	21154641	2	1	neutral	0

F48: Resection 2

Chrom	start	stop	major copy #	minor copy #	amplification	LOH	Genes
1	69090	955711	2	1	neutral	0	
1	957580	976761	3	0	gain	1	
1	976860	986941	4	0	gain	1	
1	986990	990361	3	0	gain	1	
1	1007200	1480351	2	1	neutral	0	
1	1500160	1718871	3	1	gain	0	
1	1720500	11346151	2	1	neutral	0	
1	11561050	11589951	4	0	gain	1	
1	11590000	11596731	3	0	gain	1	
1	11708750	28733971	2	1	neutral	0	
1	28759540	28856431	4	2	gain	0	
1	28857040	32164207	2	1	neutral	0	
1	32165420	32230381	3	1	gain	0	
1	32256210	33116131	2	1	neutral	0	
1	33116910	33145743	4	0	gain	1	
1	33147390	34158581	3	1	gain	0	
1	34158630	36230271	2	1	neutral	0	
1	36230820	36383931	3	0	gain	1	
1	36383980	36521341	4	0	gain	1	
1	36550530	44010801	2	1	neutral	0	
1	44019170	44087661	3	1	gain	0	
1	44118850	46651201	2	1	neutral	0	
1	46654390	46743951	4	1	gain	0	
1	46744540	55465011	2	1	neutral	0	
1	55470700	55518431	3	1	gain	0	
1	55521670	55527151	4	0	gain	1	
1	55527200	55527214	4	2	gain	0	
1	55529050	62747231	2	1	neutral	0	

1	62905540	63052231	3	0	gain	1	
1	63052280	63070461	4	0	gain	1	
1	63084380	63282401	3	0	gain	1	
1	63282450	64059311	3	1	gain	0	
1	64059360	85136911	2	1	neutral	0	
1	85136960	85724671	1	0	loss	1	
1	85724720	95323041	2	1	neutral	0	
1	95330300	95332941	4	2	gain	0	
1	95354220	103347311	2	1	neutral	0	
1	103348760	104086061	4	2	gain	0	
1	104087570	149783911	2	1	neutral	0	
1	149784830	149859431	4	0	gain	1	
1	149871620	150280641	2	1	neutral	0	
1	150297400	150321691	4	0	gain	1	
1	150325310	150980741	2	1	neutral	0	
1	150981110	151006691	5	0	gain	1	PRUNE1
1	151009210	153320401	2	1	neutral	0	
1	153330760	153410821	1	0	loss	1	
1	153412410	156023441	2	1	neutral	0	
1	156023490	156038161	3	0	gain	1	
1	156038210	156096681	4	0	gain	1	
1	156100410	156202211	2	1	neutral	0	
1	156203420	156216481	4	0	gain	1	
1	156220380	156351961	3	1	gain	0	
1	156352540	156391361	5	2	gain	0	C1Orf61
1	156437440	156722131	3	1	gain	0	
1	156737570	156770221	5	2	gain	0	PRCC
1	156776970	156891401	3	1	gain	0	
1	156891450	157665321	2	1	neutral	0	
1	157665370	157668381	1	0	loss	1	
1	157668390	167097841	2	1	neutral	0	
1	167190140	167385041	5	2	gain	0	POU2F1
1	167400920	171310961	2	1	neutral	0	
1	171481230	171560981	3	1	gain	0	
1	171605070	176660341	2	1	neutral	0	
1	176661270	176668241	5	0	gain	1	PAPPA2 segm
1	176668290	177133831	4	2	gain	0	
1	177199020	198608451	2	1	neutral	0	
1	198661480	198725291	4	2	gain	0	
1	199996980	200827181	2	1	neutral	0	
1	202304780	202544281	3	1	gain	0	
1	202549610	204295031	2	1	neutral	0	
1	204295080	205053220	3	1	gain	0	
1	205057920	205091001	5	0	gain	1	RBBP5
1	205116690	214531371	2	1	neutral	0	

1	214537860	214558131	4	2	gain	0	PTPN14
1	214560190	214638031	6	3	gain	0	PTPN14
1	214638080	214638131	5	0	gain	1	PTPN14
1	214787100	220324741	2	1	neutral	0	
1	220324950	220445671	4	0	gain	1	
1	220702170	230461161	2	1	neutral	0	
1	230468590	230493041	3	0	gain	1	
1	230513250	242162361	2	1	neutral	0	
1	242253160	242253411	4	0	gain	1	
1	242263970	243859001	2	1	neutral	0	
1	244006430	244218561	4	0	gain	1	
1	244218580	249212561	2	1	neutral	0	
2	41610	1546362	2	1	neutral	0	
2	1637980	1748221	3	0	gain	1	
2	1795640	20424621	2	1	neutral	0	
2	20451350	20647731	4	0	gain	1	
2	20818440	27354341	2	1	neutral	0	
2	27354540	27357311	4	0	gain	1	PREB
2	27357360	27357411	5	0	gain	1	PREB
2	27359930	27566001	2	1	neutral	0	
2	27566180	27657391	3	0	gain	1	
2	27657590	33623581	2	1	neutral	0	
2	33740210	33783381	4	0	gain	1	
2	33783740	38977311	2	1	neutral	0	
2	38978370	38978391	5	0	gain	1	SRSF7 3'
2	39006140	43813581	2	1	neutral	0	
2	43813840	44004021	4	2	gain	0	
2	44010660	45832551	2	1	neutral	0	
2	45879240	46207491	4	0	gain	1	
2	46211690	54087021	2	1	neutral	0	
2	54093230	54197881	4	2	gain	0	
2	54342840	73250341	2	1	neutral	0	
2	73267990	73316411	3	0	gain	1	
2	73339480	88012931	2	1	neutral	0	
2	88013570	88053151	4	0	gain	1	
2	88071690	88118131	3	0	gain	1	
2	88125180	96260851	2	1	neutral	0	
2	96261950	96589251	3	1	gain	0	
2	96589300	96657451	4	2	gain	0	
2	96687910	97779631	2	1	neutral	0	
2	97783800	97856901	4	0	gain	1	
2	97858600	97899571	4	2	gain	0	
2	97909590	98263581	3	0	gain	1	
2	98263810	101185451	2	1	neutral	0	

2	101186040	101192921	3	0	gain	1	
2	101437490	110959051	2	1	neutral	0	
2	110962480	112638391	4	2	gain	0	
2	112656320	118865891	2	1	neutral	0	
2	119600520	119752071	1	0	loss	1	
2	119914380	120319681	2	1	neutral	0	
2	120344700	121685041	4	2	gain	0	
2	121708820	136262051	2	1	neutral	0	
2	136289110	136348301	4	0	gain	1	R3HDM1
2	136354540	136481861	5	0	gain	1	R3HDM1
2	136499500	136499551	3	0	gain	1	
2	136505840	153504411	2	1	neutral	0	
2	153512820	153572611	4	0	gain	1	
2	153573750	158300511	2	1	neutral	0	
2	158390430	158454031	4	2	gain	0	
2	158485090	161056571	2	1	neutral	0	
2	161132150	161132201	3	0	gain	1	RBMS1
2	161133820	161223881	5	2	gain	0	RBMS1
2	161263980	179170971	2	1	neutral	0	
2	179184970	179368591	4	2	gain	0	
2	179391740	180819691	2	1	neutral	0	
2	180819740	190670431	3	1	gain	0	
2	190670470	209116251	2	1	neutral	0	
2	209136250	210545531	4	2	gain	0	
2	210555330	215595131	2	1	neutral	0	
2	215595180	217724411	3	1	gain	0	
2	217724620	242841471	2	1	neutral	0	

3	361460	8809704	3	1	gain	0	
3	8923040	9005061	7	0	gain	1	RAD18
3	9027210	10362701	3	1	gain	0	
3	10370500	10491221	5	2	gain	0	ATP2B2
3	10857950	11302181	3	1	gain	0	
3	11340170	11406191	5	0	gain	1	ATG7
3	11421450	20025301	3	1	gain	0	
3	20026880	35778774	2	1	neutral	0	
3	35778820	35833981	3	0	gain	1	
3	35835200	36527731	2	1	neutral	0	
3	36534650	48732851	1	0	loss	1	
3	48752750	48885021	2	0	neutral	1	
3	48895150	49043601	1	0	loss	1	
3	49044120	49045091	2	0	neutral	1	
3	49048860	49967301	1	0	loss	1	
3	50000080	50155871	2	1	neutral	0	
3	50197060	51517841	1	0	loss	1	

3	51624440	51697431	2	1	neutral	0	
3	51708320	52434441	1	0	loss	1	
3	52436300	52443901	2	0	neutral	1	
3	52446900	53892631	1	0	loss	1	
3	53892750	54676261	2	0	neutral	1	
3	54786630	63898841	2	1	neutral	0	
3	63898890	63999271	3	0	gain	1	
3	64004280	64673321	4	2	gain	0	
3	65342060	111368651	2	1	neutral	0	
3	111394100	111718421	4	2	gain	0	
3	111719620	113497651	2	1	neutral	0	
3	113499580	113524351	4	0	gain	1	
3	113528190	122160941	2	1	neutral	0	
3	122168420	122354911	3	1	gain	0	
3	122399730	124692731	2	1	neutral	0	
3	124696710	125899811	3	1	gain	0	
3	126062570	133114811	2	1	neutral	0	
3	133118930	133185781	5	0	gain	1	BFSP2
3	133191190	134267961	3	1	gain	0	
3	134268010	140419731	2	1	neutral	0	
3	140419780	141231111	3	1	gain	0	
3	141235170	141331151	5	2	gain	0	RASA2
3	141381940	142166771	2	1	neutral	0	
3	142168260	142280231	5	2	gain	0	ATR
3	142281070	142297531	4	0	gain	1	ATR
3	142383080	146254331	3	1	gain	0	
3	146303860	146323981	6	1	gain	0	PLSCR5
3	147108720	167437901	3	1	gain	0	
3	167506920	168865521	4	0	gain	1	
3	169098980	169802771	2	1	neutral	0	
3	169815020	182817141	3	1	gain	0	
3	182817190	184009191	2	1	neutral	0	
3	184009850	184059961	3	1	gain	0	
3	184060010	187419861	2	1	neutral	0	
3	187419910	190610161	3	1	gain	0	
3	190930330	190936741	4	0	gain	1	
3	190967830	197751581	3	1	gain	0	
3	197751630	197896711	2	1	neutral	0	
4	53382	2242271	2	1	neutral	0	
4	2243250	2243751	4	0	gain	1	
4	2252270	3445121	2	1	neutral	0	
4	3445770	3449961	1	0	loss	1	
4	3450970	6304191	2	1	neutral	0	
4	6325030	6374401	3	1	gain	0	

4	6377550	7765511	2	1	neutral	0
4	7770570	8129381	3	1	gain	0
4	8160410	31144451	2	1	neutral	0
4	36069530	36296621	3	0	gain	1
4	36307820	56448391	2	1	neutral	0
4	56458270	57193961	1	0	loss	1
4	57204570	57302471	2	0	neutral	1
4	57307830	70080234	1	0	loss	1
4	70146220	70160501	0	0	loss	1
4	70346360	73931121	1	0	loss	1
4	73933750	74124501	2	0	neutral	1
4	74270050	87281311	1	0	loss	1
4	87516930	87735661	2	1	neutral	0
4	87744840	190873421	1	0	loss	1
4	190874230	190947591	2	1	neutral	0

5	140430	182421	3	1	gain	0
5	191660	45645691	2	1	neutral	0
5	45695770	64096131	1	0	loss	1
5	64097130	64926531	2	0	neutral	1
5	64930740	72744181	1	0	loss	1
5	72794500	73236821	2	0	neutral	1
5	73930540	131879181	1	0	loss	1
5	131892370	132073111	2	0	neutral	1
5	132083190	137474391	1	0	loss	1
5	137474440	139752331	1	0	loss	1
5	139781560	139928641	2	0	neutral	1
5	139928820	180687511	1	0	loss	1

6	292540	292551	1	0	loss	1
6	304630	3851021	2	1	neutral	0
6	4021660	4060841	4	2	gain	0
6	4068930	12296291	2	1	neutral	0
6	12718980	13278551	4	2	gain	0
6	13281200	13621041	2	1	neutral	0
6	13622600	13697121	4	0	gain	1
6	13711170	26017821	2	1	neutral	0
6	26020720	26056550	4	0	gain	1
6	26087670	26108283	2	1	neutral	0
6	26123760	26197471	3	1	gain	0
6	26199080	26205181	4	2	gain	0
6	26216490	26225691	4	0	gain	1
6	26225710	26273561	2	1	neutral	0

6	26285400	26385501	4	2	gain	0	
6	26385550	27783181	3	1	gain	0	
6	27791910	27806441	4	0	gain	1	
6	27806490	27861591	4	2	gain	0	
6	27879030	31475051	2	1	neutral	0	
6	31475180	31475281	1	0	loss	1	
6	31477560	31584671	2	1	neutral	0	
6	31590570	31591661	3	0	gain	1	
6	31592040	31600761	4	0	gain	1	
6	31601160	31634651	3	0	gain	1	
6	31635650	43040671	2	1	neutral	0	
6	43040960	43144391	4	2	gain	0	
6	43146040	43337061	2	1	neutral	0	
6	43395720	43422841	4	2	gain	0	
6	43466740	65767561	2	1	neutral	0	
6	65767610	66205061	3	1	gain	0	
6	69348570	70098751	4	2	gain	0	
6	70386050	111346761	2	1	neutral	0	
6	111408960	114178981	3	1	gain	0	
6	114180860	114291801	3	0	gain	1	
6	114291850	128222041	2	1	neutral	0	
6	128291380	129204491	4	2	gain	0	
6	129371070	163148694	2	1	neutral	0	
6	163149270	163989461	4	2	gain	0	QKI
6	165693510	170893641	2	1	neutral	0	
7	193200	6744801	2	1	neutral	0	
7	6797310	6845661	4	0	gain	1	
7	6851590	20662911	2	1	neutral	0	
7	20662960	21468401	3	1	gain	0	
7	21468910	27578011	2	1	neutral	0	
7	27582590	29160661	3	1	gain	0	
7	29234560	44161941	2	1	neutral	0	
7	44162780	44163251	3	0	gain	1	
7	44178520	47619441	2	1	neutral	0	
7	47694880	47971641	4	2	gain	0	
7	47976450	48273721	2	1	neutral	0	
7	48278840	48685071	3	1	gain	0	
7	49815040	94898011	2	1	neutral	0	
7	94898520	94918001	7	3	gain	0	PPP1R9A
7	94919410	99526971	2	1	neutral	0	
7	99564630	99569621	4	0	gain	1	
7	99573570	106791461	2	1	neutral	0	
7	106793630	106810571	4	2	gain	0	

7	106814900	121011461	2	1	neutral	0	
7	121012170	121701241	3	1	gain	0	
7	121716550	124430651	2	1	neutral	0	
7	124464010	124537231	4	0	gain	1	
7	126079180	152457011	2	1	neutral	0	
7	152480280	152551601	4	0	gain	1	
7	152841290	158937421	2	1	neutral	0	
8	190900	26221391	2	1	neutral	0	
8	26223830	26267921	4	0	gain	1	
8	26365180	37635591	2	1	neutral	0	
8	37654790	37687061	1	0	loss	1	
8	37687370	48508531	2	1	neutral	0	
8	48511510	48874231	3	0	gain	1	
8	48874620	53536401	2	1	neutral	0	
8	53537280	53598011	3	1	gain	0	
8	53852470	59059901	2	1	neutral	0	
8	59323950	62626881	3	1	gain	0	
8	62626920	67342461	2	1	neutral	0	
8	67344760	67352431	3	1	gain	0	
8	67355040	67364341	4	0	gain	1	
8	67365950	67511341	3	1	gain	0	
8	67513940	97285581	2	1	neutral	0	
8	97296340	97345791	4	0	gain	1	
8	97506500	110477471	2	1	neutral	0	
8	110478810	110542311	4	2	gain	0	
8	110563060	110587141	3	0	gain	1	
8	110588240	117864891	2	1	neutral	0	
8	117864940	117878931	3	0	gain	1	
8	117950490	141595371	2	1	neutral	0	
8	141669570	141727801	5	0	gain	1	PTK2/FAK
8	141745350	141935811	4	2	gain	0	PTK2/FAK
8	142138810	143436051	2	1	neutral	0	
8	143545560	143695631	3	0	gain	1	
8	143740270	145584671	2	1	neutral	0	
8	145597790	145618281	4	2	gain	0	
8	145618540	146279511	2	1	neutral	0	
9	14810	135021	4	1	gain	0	WASHC1; FOXD4
9	146110	33472401	2	1	neutral	0	
9	8507440	19490191	2	1	neutral	0	
9	33473790	33799171	4	2	gain	0	
9	33817760	38424221	2	1	neutral	0	
9	38543250	70914501	3	1	gain	0	
9	70917870	72879321	2	1	neutral	0	

9	72882840	74834421	1	0	loss	1	
9	74838040	98011551	2	1	neutral	0	
9	98209190	98279091	4	2	gain	0	PTCH1
9	98638290	104086331	2	1	neutral	0	
9	104124710	104133671	4	2	gain	0	
9	104151740	112082721	2	1	neutral	0	
9	112141840	112219491	4	0	gain	1	
9	112219580	123555221	2	1	neutral	0	
9	123580190	123595691	4	2	gain	0	
9	123605020	125719481	2	1	neutral	0	
9	125746770	125852661	4	0	gain	1	
9	125860020	127912161	2	1	neutral	0	
9	127915820	127996221	4	0	gain	1	
9	127998870	130550331	2	1	neutral	0	
9	130550380	130678691	3	1	gain	0	
9	130684130	130692121	4	1	gain	0	PIP5KL1
9	130693010	130693031	5	0	gain	1	PIP5KL1
9	130698000	131711521	3	1	gain	0	
9	131715060	132404471	2	1	neutral	0	
9	132481510	132510991	4	0	gain	1	
9	132515170	134371231	2	1	neutral	0	
9	134379610	134398471	4	2	gain	0	
9	134400430	134612901	3	1	gain	0	
9	134615160	135919271	2	1	neutral	0	
9	135926170	135933521	1	0	loss	1	GTF3C5
9	135936140	138852971	2	1	neutral	0	
9	138903370	139438521	3	1	gain	0	
9	139440170	139726821	2	1	neutral	0	RABL6
9	139728180	139733511	5	2	gain	0	RABL6
9	139733560	139744041	4	0	gain	1	RABL6
9	139744470	139867151	2	1	neutral	0	
9	139872030	139875291	4	0	gain	1	
9	139878040	140110831	3	1	gain	0	
9	140114880	141109841	2	1	neutral	0	
10	93000	5904641	3	1	gain	0	
10	5920050	5937121	20	0	gain	1	ANKRD16
10	5944990	22608971	3	1	gain	0	
10	22615360	22618451	20	0	gain	1	BMI1
10	22634540	23235181	3	0	gain	1	
10	23244740	23270621	20	0	gain	1	ARMC3 5'
10	23287080	27035371	3	1	gain	0	
10	27037500	27066151	20	0	gain	1	ABI1
10	27112070	33190551	3	1	gain	0	
10	33195990	73550151	2	1	neutral	0	

10	73550890	74619091	3	1	gain	0	
10	74620180	90774181	2	1	neutral	0	
10	90966230	119307731	1	0	loss	1	
10	119768510	119800041	2	1	neutral	0	
10	119805330	120931991	1	0	loss	1	
10	120933250	120938301	2	0	neutral	1	
10	120967430	121355971	1	0	loss	1	
10	121411190	121436481	2	1	neutral	0	
10	121510590	122348971	1	0	loss	1	
10	122610940	122646301	20	0	gain	1	WDR11
10	122646340	122666361	19	0	gain	1	WDR11
10	122668070	123239541	13	0	gain	1	WDR11, FGFR2 ends
10	123241620	123298201	20	0	gain	1	FGFR2
10	123310800	123662111	20	0	gain	1	FGFR2; ATE1
10	123670420	123686751	17	0	gain	1	ATE1
10	123687360	123687921	14	0	gain	1	ATE1
10	123718850	123848251	14	1	gain	0	NSMCE4A; TACC2
10	123892130	123954661	19	0	gain	1	TACC2
10	123969920	123989921	1	0	loss	1	TACC2
10	123996910	124054941	20	0	gain	1	TACC2;BTBD16 segm
10	124057270	124340411	1	0	loss	1	
10	124345580	124352091	0	0	loss	1	DMBT1 segmental
10	124357490	135440221	1	0	loss	1	
11	193100	44958901	1	0	loss	1	
11	44959110	44959161	0	0	loss	1	TP53I11 segm
11	44959760	55036771	1	0	loss	1	
11	55110680	64035021	2	1	neutral	0	
11	64037680	64053311	4	0	gain	1	
11	64053490	64084831	2	1	neutral	0	
11	64084920	64088531	4	0	gain	1	
11	64089060	64444501	2	1	neutral	0	
11	64453120	64514801	3	0	gain	1	
11	64517850	64527331	4	0	gain	1	
11	64532960	65383897	2	1	neutral	0	
11	65384300	65410091	3	0	gain	1	
11	65412430	67141571	2	1	neutral	0	
11	67159520	67168691	3	0	gain	1	
11	67169200	73118681	2	1	neutral	0	
11	73120540	73359131	3	0	gain	1	
11	73359740	73471101	4	0	gain	1	
11	73471130	73844571	3	1	gain	0	
11	73849770	74109201	5	2	gain	0	PPME
11	74168300	74379831	3	1	gain	0	
11	74379880	82444771	2	1	neutral	0	

11	82535950	82611431	5	2	gain	0	PRCP
11	82625780	108093261	2	1	neutral	0	
11	108098350	108236211	3	1	gain	0	
11	108253480	111179151	2	1	neutral	0	
11	111224990	111229621	4	0	gain	1	
11	111233910	111597741	2	1	neutral	0	
11	111608180	111637081	4	2	gain	0	
11	111657120	112088591	2	1	neutral	0	
11	112097170	113092041	3	1	gain	0	
11	113101920	120178291	2	1	neutral	0	
11	120180140	120355781	3	0	gain	1	
11	120531030	124971391	2	1	neutral	0	
11	124972030	125781311	3	0	gain	1	
11	125781360	126396541	3	1	gain	0	
11	126432730	129763011	2	1	neutral	0	
11	129772210	129827791	4	2	gain	0	
11	129830790	134257701	2	1	neutral	0	
12	176050	7362811	2	1	neutral	0	
12	7456930	7480941	4	2	gain	0	
12	7510000	9305521	2	1	neutral	0	
12	9305720	9711921	3	1	gain	0	
12	9712710	10532351	2	1	neutral	0	
12	10539510	10569321	5	0	gain	1	KLRC4
12	10569360	10588751	6	2	gain	0	KLRC3, KLRC2
12	10598900	15637161	2	1	neutral	0	
12	15650180	15734721	4	2	gain	0	
12	15739830	16115851	3	0	gain	1	
12	16135250	23737531	2	1	neutral	0	
12	23757320	25101861	4	0	gain	1	
12	25147230	25801481	2	1	neutral	0	
12	26208280	26225801	4	2	gain	0	
12	26231970	31256941	2	1	neutral	0	
12	31267690	31267791	5	2	gain	0	OVOS2
12	31267840	31285231	4	1	gain	0	OVOS2
12	31286050	31359084	6	2	gain	0	OVOS2
12	31435650	45004731	2	1	neutral	0	
12	45059270	45797011	1	0	loss	1	
12	45797230	46287261	2	0	neutral	1	
12	46287310	48119191	1	0	loss	1	
12	48131350	48172961	2	0	neutral	1	
12	48173930	49251981	1	0	loss	1	
12	49254780	49393061	2	1	neutral	0	
12	49396690	49412801	4	2	gain	0	
12	49415560	49525081	2	1	neutral	0	

12	49578800	49582701	4	2	gain	0	
12	49621740	50369361	2	1	neutral	0	
12	50384050	50384101	3	0	gain	1	
12	50384470	50395011	4	0	gain	1	
12	50396030	50481251	4	2	gain	0	
12	50482310	53594221	2	1	neutral	0	
12	53605460	53646031	4	0	gain	1	
12	53646220	53901251	2	1	neutral	0	
12	53910920	54066411	4	2	gain	0	
12	54069840	56881871	2	1	neutral	0	
12	56915810	57109861	3	1	gain	0	
12	57109910	57167941	4	2	gain	0	
12	57175760	62997081	2	1	neutral	0	
12	63042270	63544621	4	2	gain	0	
12	63954300	70824421	2	1	neutral	0	
12	70915260	71314161	4	2	gain	0	
12	71519120	80169761	2	1	neutral	0	
12	80170930	82752601	4	2	gain	0	
12	82780590	93101491	2	1	neutral	0	
12	93115250	93169891	4	0	gain	1	
12	93170620	93804611	3	1	gain	0	
12	93804830	105388461	2	1	neutral	0	
12	105418210	105467741	3	0	gain	1	
12	105478170	108086641	2	1	neutral	0	
12	108086780	108097521	4	2	gain	0	
12	108098420	108136101	3	0	gain	1	
12	108136980	108150711	4	0	gain	1	
12	108154290	111886081	2	1	neutral	0	
12	111890620	111947751	3	0	gain	1	
12	111947800	112140071	3	1	gain	0	
12	112143550	112856931	2	1	neutral	0	
12	112884070	112942541	4	0	gain	1	
12	113266130	121014451	2	1	neutral	0	
12	121017120	121019021	4	2	gain	0	
12	121019150	133810901	2	1	neutral	0	
13	19748010	32885911	2	1	neutral	0	
13	32890590	32972791	4	2	gain	0	BRCA2
13	32976020	37573431	2	1	neutral	0	
13	37574950	37576441	4	0	gain	1	
13	37576630	38143931	3	1	gain	0	
13	38144720	38172851	4	0	gain	1	
13	38211040	110960291	2	1	neutral	0	
13	110960420	111099201	3	0	gain	1	
13	111102030	115091751	2	1	neutral	0	

14	19377600	27066911	2	1	neutral	0	
14	29236490	30098251	4	2	gain	0	
14	30098290	30194851	4	0	gain	1	
14	30396460	35550431	2	1	neutral	0	
14	35554800	35579131	4	0	gain	1	PPP2R3C (PP2A)
14	35579730	35782241	2	1	neutral	0	
14	35783570	35786511	6	0	gain	1	PSMA6 3'
14	35871220	45542751	2	1	neutral	0	
14	45564450	45583441	4	0	gain	1	
14	45583700	51372261	2	1	neutral	0	
14	51374990	51410981	5	2	gain	0	PYGL
14	51411030	51411081	5	0	gain	1	PYGL
14	51444010	65528001	2	1	neutral	0	
14	65541850	65560531	1	0	loss	1	
14	65568270	73465021	2	1	neutral	0	
14	73490740	73739361	3	0	gain	1	
14	73743290	77737251	2	1	neutral	0	
14	77743720	77787521	5	2	gain	0	POMT2
14	77787710	95236351	2	1	neutral	0	
14	95556830	95599801	3	0	gain	1	
14	95657900	105996131	2	1	neutral	0	
15	20739500	38773801	2	1	neutral	0	
15	38776460	38852181	4	0	gain	1	
15	38856800	40453451	2	1	neutral	0	
15	40457260	40505641	4	2	gain	0	
15	40509700	42276741	2	1	neutral	0	
15	42276790	42447771	3	0	gain	1	
15	42448640	66048781	2	1	neutral	0	
15	66161790	66180141	4	0	gain	1	
15	66190280	73075991	2	1	neutral	0	
15	73345020	73345121	1	0	loss	1	
15	73408880	77224801	2	1	neutral	0	
15	77227870	77532191	3	1	gain	0	
15	77532240	77697101	4	2	gain	0	
15	77709640	91185371	2	1	neutral	0	
15	91290620	91358481	4	0	gain	1	
15	91418970	91504991	2	1	neutral	0	
15	91510360	91528031	4	2	gain	0	
15	91537640	93198851	2	1	neutral	0	
15	93444460	93448201	4	0	gain	1	
15	93467540	102462851	2	1	neutral	0	
16	97430	640401	2	1	neutral	0	

16	667210	677601	4	0	gain	1	
16	681260	2240351	2	1	neutral	0	
16	2246340	2246441	1	0	loss	1	
16	2255690	7383051	2	1	neutral	0	
16	7560180	7760731	5	2	gain	0	
16	8619510	28890881	2	1	neutral	0	
16	28891380	28915751	3	0	gain	1	
16	28916270	31336081	2	1	neutral	0	
16	31336280	31373991	1	0	loss	1	
16	31374040	48119611	2	1	neutral	0	
16	48120660	48265991	3	1	gain	0	
16	48278300	53191451	2	1	neutral	0	
16	53241910	53243701	4	0	gain	1	
16	53256560	53469651	2	1	neutral	0	
16	53472930	53524201	3	1	gain	0	
16	53526330	56544821	2	1	neutral	0	
16	56545070	56553761	3	0	gain	1	
16	56599040	71968021	2	1	neutral	0	
16	71969260	71971301	3	0	gain	1	
16	71976470	72027221	4	2	gain	0	
16	72032170	75258761	2	1	neutral	0	
16	75263410	75282211	4	2	gain	0	
16	75284570	84360541	2	1	neutral	0	
16	84402230	84497321	4	2	gain	0	
16	84513520	90094101	2	1	neutral	0	
16	90095400	90095741	5	0	gain	1	GAS8 segm
16	90097590	90109711	2	1	neutral	0	
16	90124120	90142301	4	2	gain	0	
17	6010	5138031	2	0	neutral	1	
17	5138530	5357241	2	1	neutral	0	
17	5357290	12909291	2	0	neutral	1	incl TP53
17	12913930	16120691	2	1	neutral	0	
17	16120740	20922451	2	0	neutral	1	
17	20922490	21438251	2	1	neutral	0	
17	21438690	21438791	1	0	loss	1	
17	21454260	32646571	2	1	neutral	0	
17	32647290	32690161	1	0	loss	1	
17	32905890	38131211	2	1	neutral	0	
17	38131260	38573941	3	1	gain	0	
17	38574030	44833181	2	1	neutral	0	
17	44845690	45206861	1	0	loss	1	
17	45209630	45209731	0	0	loss	1	
17	45214520	45777061	2	1	neutral	0	
17	45785760	46629831	1	0	loss	1	

17	46654090	47925361	2	1	neutral	0	
17	48046840	56084461	3	1	gain	0	
17	56164460	56358111	2	1	neutral	0	
17	56379630	56770151	3	1	gain	0	
17	56772290	57050251	5	0	gain	1	RAD51C; PPM1E
17	57057340	57311861	3	1	gain	0	
17	57311890	58508601	2	1	neutral	0	
17	58524950	58603261	3	0	gain	1	
17	58677780	58756871	5	0	gain	1	PPM1D
17	58761350	65241621	3	1	gain	0	incl AXIN2; PRKCA
17	65336960	65362631	6	2	gain	0	PSMD12
17	65374270	65900011	3	1	gain	0	
17	65900820	65942031	5	0	gain	1	BPTF
17	65943850	71361451	3	1	gain	0	
17	71361500	71384131	3	0	gain	1	
17	71384170	79881061	3	1	gain	0	
17	79890760	79895081	6	0	gain	1	PYCR1
17	79898700	81052251	3	1	gain	0	
17	81052300	81052301	2	1	neutral	0	
18	158700	52969851	2	1	neutral	0	
18	52988900	53089741	4	0	gain	1	
18	53128250	76740331	2	1	neutral	0	
18	76752080	76757291	4	2	gain	0	
18	76829410	78005211	2	1	neutral	0	
19	281390	821541	2	1	neutral	0	
19	827850	841061	4	2	gain	0	
19	843460	5151501	2	1	neutral	0	
19	5206790	5274311	4	2	gain	0	
19	5286060	13039571	2	1	neutral	0	
19	13039620	13320231	3	1	gain	0	
19	13320280	14163061	2	1	neutral	0	
19	14164420	14192801	3	1	gain	0	
19	14193280	17389851	2	1	neutral	0	
19	17392570	17412171	4	2	gain	0	
19	17416710	38861421	2	1	neutral	0	
19	38865250	38874001	3	0	gain	1	
19	38875060	43098971	2	1	neutral	0	
19	43228140	43772291	3	1	gain	0	
19	43773520	43773571	3	0	gain	1	
19	43857870	46195251	2	1	neutral	0	
19	46195970	46206271	4	0	gain	1	
19	46214950	46879811	2	1	neutral	0	
19	46886030	46894431	3	0	gain	1	

19	46914450	47177981	2	1	neutral	0	
19	47178290	47217571	4	0	gain	1	
19	47219390	49222281	2	1	neutral	0	
19	49224060	49241191	4	2	gain	0	
19	49242220	50063931	2	1	neutral	0	
19	50086480	50128821	4	0	gain	1	
19	50138840	52133441	2	1	neutral	0	
19	52146610	52227181	1	0	loss	1	
19	52249200	54584601	2	1	neutral	0	
19	54598490	54604081	4	0	gain	1	
19	54606190	55693245	2	1	neutral	0	
19	55693390	55697021	3	0	gain	1	
19	55697230	55791092	3	1	gain	0	
19	55795340	59083971	2	1	neutral	0	
20	68350	1559331	2	1	neutral	0	
20	1578310	1592351	20	0	gain	1	SIRPB1
20	1600520	2847181	2	1	neutral	0	
20	2903910	3002851	4	2	gain	0	PTPRA
20	3003340	3388281	2	1	neutral	0	
20	3388390	3388441	1	0	loss	1	
20	3451720	3662661	3	1	gain	0	
20	3669210	20350441	2	1	neutral	0	
20	20373750	20693121	4	0	gain	1	RALGAPA2
20	21106680	37063991	2	1	neutral	0	
20	37117080	37137771	3	0	gain	1	
20	37137820	37195841	4	0	gain	1	
20	37198530	37275751	3	1	gain	0	
20	37277290	40179971	2	1	neutral	0	
20	40709520	42089211	3	1	gain	0	
20	42089350	61869261	2	1	neutral	0	
20	61869310	62899341	3	1	gain	0	
20	62904580	62904931	3	0	gain	1	
21	10906910	15013871	2	1	neutral	0	
21	15481280	48084261	1	0	loss	1	
22	16258190	24323191	2	1	neutral	0	
22	24373140	24384221	4	0	gain	1	
22	24431965	25024801	2	1	neutral	0	
22	25115440	25202311	4	0	gain	1	
22	25202410	41636891	2	1	neutral	0	
22	41642610	41681651	4	2	gain	0	RANGAP1
22	41716670	46372871	2	1	neutral	0	
22	46449630	46631271	4	2	gain	0	

22	46640990	50906321	2	1	neutral	0	
22	50906810	50913271	1	0	loss	1	
22	50920280	51183581	2	1	neutral	0	
22	51207210	51220721	6	0	gain	1	RABL2B
X	295060	2799251	2	1	neutral	0	
X	2825320	6452241	3	1	gain	0	
X	6968340	18690181	2	1	neutral	0	
X	18725900	18845601	4	0	gain	1	
X	18911610	37263021	2	1	neutral	0	
X	37265470	37312841	4	0	gain	1	
X	37431130	39937191	2	1	neutral	0	
X	40440320	40464971	4	0	gain	1	
X	40482820	48755061	2	1	neutral	0	
X	48755350	48760361	4	0	gain	1	
X	48761040	54521811	2	1	neutral	0	
X	54521860	55021331	1	0	loss	1	
X	55024840	55054231	2	1	neutral	0	
X	55102480	55117871	4	0	gain	1	PAGE2B
X	55170220	76711971	2	1	neutral	0	
X	76763830	77270211	3	0	gain	1	
X	77271250	77294391	4	0	gain	1	
X	77294440	77302041	3	0	gain	1	
X	77359840	107320581	2	1	neutral	0	
X	107328210	107335101	3	1	gain	0	
X	107369310	119010491	2	1	neutral	0	
X	119037190	119053921	3	0	gain	1	
X	119054470	135789171	2	1	neutral	0	
X	135790840	135961581	3	1	gain	0	
X	136112310	152752241	2	1	neutral	0	
X	152770090	152845741	3	0	gain	1	
X	152853830	153649301	2	1	neutral	0	
X	153657040	153663821	3	0	gain	1	
X	153664030	155004401	2	1	neutral	0	
Y	21154300	21154641	2	1	neutral	0	

F48: LM-S4

Chrom	start	stop	major copy #	minor copy #	amplification	LOH	Genes
1	63200	156040341	2	1	neutral	0	
1	156084690	156848107	3	1	gain	0	
1	156848120	157104501	3	0	gain	1	
1	161284160	203281241	2	1	neutral	0	
1	204393960	204394831	3	0	gain	1	
1	204394880	204527831	3	1	gain	0	
1	205597250	248867391	2	1	neutral	0	

2	27500	15747461	2	1	neutral	0	
2	16078480	16089591	11	0	gain	1	MYCNOS
2	16089640	16089681	11	0	gain	1	MYCN
2	17941200	242801001	2	1	neutral	0	
3	182950	30733101	3	1	gain	0	
3	37034730	53264521	1	0	loss	1	
3	55511600	197203751	2	1	neutral	0	
4	103220	55991471	2	1	neutral	0	
4	68614880	190435371	1	0	loss	1	
5	75880	45697531	2	1	neutral	0	
5	49960600	180671221	1	0	loss	1	incl APC, PDGFRB,
6	189070	170242981	2	1	neutral	0	
7	81330	158827481	2	1	neutral	0	
8	187870	145813491	2	1	neutral	0	
9	211400	127951981	2	1	neutral	0	incl CDKN2A/B
9	130552840	130587641	3	1	gain	0	
9	130587960	137328451	2	1	neutral	0	
9	139390510	141017411	3	1	gain	0	
10	268340	14940761	3	1	gain	0	
10	37968210	73511611	2	1	neutral	0	
10	73512700	73533201	3	1	gain	0	
10	81926650	90774191	2	1	neutral	0	incl PTEN
10	91061840	120829001	1	0	loss	1	
10	123237860	123357591	10	1	gain	0	FGFR2
10	129897440	135288881	1	0	loss	1	
11	193030	48157941	1	0	loss	1	
11	56103420	122928691	2	1	neutral	0	
11	125495640	126136041	3	0	gain	1	
11	128332240	134672071	2	1	neutral	0	
12	192150	39138001	2	1	neutral	0	
12	46123600	46298851	1	0	loss	1	
12	49334690	133263921	2	1	neutral	0	
13	20138860	114292571	2	1	neutral	0	
14	20834740	106580691	2	1	neutral	0	
15	22980610	101984311	2	1	neutral	0	
16	131040	89987231	2	1	neutral	0	
17	700930	713071	2	0	neutral	1	
17	713110	47700181	2	1	neutral	0	incl TP53
17	48712270	80840621	3	1	gain	0	
18	193090	77213191	2	1	neutral	0	
19	426900	59067621	2	1	neutral	0	
20	351870	62176231	2	1	neutral	0	
21	15448750	36421211	1	0	loss	1	
21	37507480	37518811	2	0	neutral	1	
21	39755310	47806621	1	0	loss	1	

22	18212990	50710701	2	1	neutral	0	
X	296370	155011961	2	1	neutral	0	

F48: DI-S6

Chrom	start	stop	major copy #	minor copy #	amplification	LOH	Genes
1	63200	206905111	2	1	neutral	0	
1	212619270	248867391	3	1	gain	0	H3F3A, FH, AKT3
2	27500	242801001	2	1	neutral	0	
3	182950	197203751	2	1	neutral	0	
4	103220	1913801	2	1	neutral	0	
4	1913850	75184081	3	1	gain	0	incl PDGFRA, KIT, KDR
4	75186180	190435371	1	0	loss	1	incl. FBXW7
5	75880	180671221	2	1	neutral	0	
6	189070	31137235	3	1	gain	0	
6	31543500	33289681	1	0	loss	1	
6	34517360	43752391	2	1	neutral	0	
6	44224850	57496146	4	2	gain	0	
6	62386950	170242981	2	1	neutral	0	
7	81330	92241701	2	1	neutral	0	
7	92244440	92462641	1	0	loss	1	CDK6
7	92735880	158827481	2	1	neutral	0	
8	187870	145813491	2	1	neutral	0	
9	211400	141017411	2	1	neutral	0	incl CDKN2A/B
10	268340	120829001	2	1	neutral	0	incl PTEN
10	123237860	123357591	1	0	loss	1	FGFR2
10	129897440	135288881	2	1	neutral	0	
11	193030	56113661	1	0	loss	1	
11	56128700	134672071	2	1	neutral	0	
12	192150	133263921	2	1	neutral	0	
13	20138860	114292571	2	1	neutral	0	
14	20834740	106580691	2	1	neutral	0	
15	22980610	101984311	2	1	neutral	0	
16	131040	89987231	2	1	neutral	0	
17	700930	29448098	2	0	neutral	1	incl TP53
17	29448130	37700561	2	1	neutral	0	
17	37855780	37884281	1	0	loss	1	ERBB2
17	38146080	80840621	2	1	neutral	0	
18	193090	77213191	3	1	gain	0	
19	426900	59067621	2	1	neutral	0	
20	351870	62176231	2	1	neutral	0	
21	15448750	30339321	3	1	gain	0	
21	30678000	34783841	2	1	neutral	0	
21	34787180	47806621	1	0	loss	1	
22	18212990	23229221	2	1	neutral	0	
22	23521900	23654437	1	0	loss	1	

22	23657720	50710701	2	1	neutral	0	
X	296370	76711191	2	1	neutral	0	
X	76763810	101409371	1	0	loss	1	ATRX, BTK
X	101409410	155011961	2	1	neutral	0	

M60

Chrom	start	stop	major copy #	minor copy #	amplification	LOH	Genes
1	63200	69581	5	0	gain	1	
1	931290	47803771	2	1	neutral	0	
1	51433620	59250453	4	2	gain	0	CDKN2C, JUN
1	59252150	248867391	2	1	neutral	0	
2	27500	242801001	2	1	neutral	0	
3	182950	30733101	2	1	neutral	0	
3	37034730	37035901	3	1	gain	0	
3	37038090	189507621	2	1	neutral	0	
3	189526050	189582211	3	0	gain	1	
3	189584460	197203751	2	1	neutral	0	
4	103220	190435371	2	1	neutral	0	
5	75880	138269731	2	1	neutral	0	
5	138269780	161495111	2	1	neutral	0	
5	170732340	170741661	1	0	loss	1	
5	170814940	180039591	2	1	neutral	0	
5	180040000	180047261	1	0	loss	1	
5	180047310	180218701	2	1	neutral	0	
5	180652170	180671221	3	0	gain	1	incl. RACK1
6	189070	219541	3	1	gain	0	
6	393140	18149351	2	1	neutral	0	
6	19835940	26157101	4	2	gain	0	
6	26204860	26205161	4	0	gain	1	
6	31137090	43738931	2	1	neutral	0	
6	43742060	44224992	4	2	gain	0	VEGFA
6	44232000	170199281	2	1	neutral	0	
6	170205440	170205574	3	0	gain	1	
6	170211810	170242981	2	0	neutral	1	
7	81330	6442031	3	1	gain	0	
7	6442080	19148881	4	0	gain	1	ETV1
7	19153440	55272228	3	1	gain	0	
7	55272826	63719961	2	1	neutral	0	
7	63721350	158827481	3	1	gain	0	
8	187870	145813491	2	1	neutral	0	
9	211400	238421	2	1	neutral	0	
9	5021970	5570001	1	0	loss	1	
9	8317860	8733861	2	1	neutral	0	
9	20346430	20622291	1	0	loss	1	MLLT3
9	21802720	21990458	0	0	loss	1	MTAP

9	21990490	22010071	0	0	loss	1	CDKN2A/B
9	32614450	141017411	2	1	neutral	0	
10	268340	135288881	1	0	loss	1	
11	193030	134672071	2	1	neutral	0	
12*	192150	68055231	2	1	neutral	0	
12	69202220	69203071	15	0	gain	1	SLC35E3, MDM2
12	69207320	69240301	12	0	gain	1	MDM2, SPSF6
12	69240340	69986821	14	0	gain	1	YEATS4, FRS2, CCT2,
12	85285870	133263921	2	1	neutral	0	BEST3, RAB3IP,
13	20138860	114292571	2	1	neutral	0	MYRFL, CNOT2,
14	20834740	106580691	2	1	neutral	0	KCNMB4, PTPRB,
15	22980610	93563481	2	1	neutral	0	PTPRR
15	93563530	93567941	3	0	gain	1	
15	101967730	101984311	2	1	neutral	0	
16	131040	89987231	2	1	neutral	0	
17	700930	80840621	2	1	neutral	0	
18	193090	77213191	2	1	neutral	0	
19	426900	42799361	3	1	gain	0	
19	44047530	49562721	2	0	neutral	1	
19	49562770	53799741	2	1	neutral	0	
19	55856780	59067621	3	1	gain	0	
20	351870	62176231	2	1	neutral	0	
21	15448750	47806621	2	1	neutral	0	
22	18212990	50710701	2	1	neutral	0	
X	296370	48650341	1	0	loss	1	
X	48650390	65884976	2	0	neutral	1	
X	65885020	155011961	2	1	neutral	0	
12*	p12	p11	high gain	CAPRIN2, IPO8, ERGIC2, FAR2, CCDC91, PTHLH			

F62

Chrom	start	stop	major copy #	minor copy #	amplification	LOH	Genes
1	63200	931431	5	0	gain	1	
1	932020	2494721	2	1	neutral	0	
1	6475520	8925541	1	0	loss	1	
1	9770500	223300471	2	1	neutral	0	
1	226019560	248867391	1	0	loss	1	
2	27500	198288711	2	1	neutral	0	
2	198299660	198299751	1	0	loss	1	
2	202122940	242801001	2	1	neutral	0	
3	182950	10183871	2	1	neutral	0	
3	10186940	10203221	4	0	gain	1	VHL
3	12626000	197203751	2	1	neutral	0	
4	103220	109631	3	0	gain	1	
4	124310	177623911	2	1	neutral	0	
4	185309900	185350221	1	0	loss	1	IRF2
4	187509730	190435371	2	1	neutral	0	

5	75880	161495111	2	1	neutral	0	
5	170732340	170870681	1	0	loss	1	NPM1, FGF18
5	172657080	180671221	2	1	neutral	0	
6	189070	32805441	2	1	neutral	0	
6	32805500	32816851	1	0	loss	1	
6	32816900	43742111	2	1	neutral	0	
6	43745190	43752391	3	0	gain	1	VEGFA
6	44224850	152446491	2	1	neutral	0	
6	157099050	163991741	1	0	loss	1	ARID1B to...QKI
6	170199140	170230621	4	2	gain	0	
6	170242880	170242981	5	0	gain	1	
7	81330	1533581	5	2	gain	0	
7	2946260	26254191	3	1	gain	0	
7	27202230	28106961	2	1	neutral	0	
7	30673470	66452081	3	1	gain	0	
7	66452120	80422761	2	1	neutral	0	
7	80422810	116399481	3	1	gain	0	
7	116399530	117120131	2	1	neutral	0	
7	117120180	158827481	3	1	gain	0	
8	187870	31497991	2	1	neutral	0	
8	32406230	41906381	1	0	loss	1	incl. FGFR1
8	42559520	145743171	2	1	neutral	0	
8	145771140	145813491	3	0	gain	1	
9	211400	20622291	1	0	loss	1	
9	21802720	21815471	0	0	loss	1	MTAP
9	21815520	21998661	0	0	loss	1	CDKN2A
9	21998690	22010071	0	0	loss	1	CDKN2B
9	32614450	35079521	1	0	loss	1	
9	36840510	139440251	2	1	neutral	0	
9	140971610	141017411	4	2	gain	0	CACNA1B
10	268340	135288881	1	0	loss	1	
11	193030	8706441	2	1	neutral	0	
11	8958620	8969341	4	2	gain	0	
11	9595470	134672071	2	1	neutral	0	
12	192150	69240341	2	1	neutral	0	
12	69753730	69986821	20	0	gain	1	YEATS4, FRS2
12	85285870	133263921	2	1	neutral	0	
13	20138860	114292571	2	1	neutral	0	
14	20834740	21961001	2	1	neutral	0	
14	22872080	22937725	3	1	gain	0	TRA
14	22942080	106580691	2	1	neutral	0	
15	22980610	42128921	2	1	neutral	0	
15	45003730	49723481	2	0	neutral	1	
15	49723520	67482891	2	0	neutral	1	
15	74287140	93534731	3	1	gain	0	

15	93534770	93563531	3	0	gain	1	
15	93567590	101984311	4	0	gain	1	
16	131040	89987231	2	1	neutral	0	
17	700930	1356611	4	2	gain	0	NXN, CRK
17	1358050	74730911	2	1	neutral	0	
17	74732230	74733471	1	0	loss	1	
17	76217020	80840621	2	1	neutral	0	
18	193090	77213191	2	1	neutral	0	
19	426900	59067621	2	1	neutral	0	
20	351870	62176231	3	1	gain	0	
21	15448750	39947611	2	1	neutral	0	
21	40181940	40194831	1	0	loss	1	ETS2
21	42838030	47806621	2	1	neutral	0	
22	18212990	50710701	1	0	loss	1	
X	296370	1315021	2	1	neutral	0	
X	1317400	48651661	1	0	loss	1	
X	48651710	155011961	1	0	loss	1	

F72

Chrom	start	stop	major copy #	minor copy #	amplification	LOH	Genes
1	10848734	238949947	2	1	neutral	0	
2	1285093	14489828	2	0	neutral	1	
2	16778060	84560538	1	0	loss	1	
2	97124454	238205324	2	1	neutral	0	
2	241801051	243800849	4	0	gain	1	
3	8063782	189322919	2	1	neutral	0	
4	7716506	182822085	2	0	neutral	1	incl. FGFR3
5	3095541	177278996	2	1	neutral	0	
6	2285947	164850346	1	0	loss	1	
7	6431176	152477635	2	0	neutral	1	
8	3516707	144444235	2	1	neutral	0	
9	893192	8052019	2	1	neutral	0	
9	9684934	19671217	1	0	loss	1	MLLT3
9	21107896	23004294	0	0	loss	1	MTAP, CDKN2A/B
9	36950568	136681293	2	1	neutral	0	
10	4854634	136278064	1	0	loss	1	
11	5572191	129292910	2	1	neutral	0	
12	5515020	127941051	2	1	neutral	0	
13	27671156	106760275	2	1	neutral	0	
14	27694416	99721015	2	1	neutral	0	
15	26538893	101133767	2	1	neutral	0	
16	7319535	82798736	2	0	neutral	1	
17	3660900	34740591	2	1	neutral	0	
17	36898531	38841529	1	0	loss	1	ERBB2
17	41561643	77425058	2	1	neutral	0	

18	6354698	71051583	3	1	gain	0	
19	5118157	54376364	2	1	neutral	0	
20	5297818	57230283	2	1	neutral	0	
21	18037379	45217992	2	1	neutral	0	
22	20812806	48110885	2	1	neutral	0	
X	3870312	41396709	3	1	gain	0	
X	47522251	52934070	1	0	loss	1	
X	57512027	150949464	2	1	neutral	0	

F75

Chrom	start	stop	major copy #	minor copy #	amplification	LOH	Genes
1	69090	976761	2	1	neutral	0	
1	976860	977011	4	0	gain	1	
1	977060	981541	5	0	gain	1	
1	981590	990361	4	0	gain	1	
1	1007200	11346151	2	1	neutral	0	
1	11561050	11596731	4	2	gain	0	
1	11708750	12888511	2	1	neutral	0	
1	12907260	13036731	0	0	loss	1	
1	13141130	17690221	2	1	neutral	0	
1	17698740	18152771	3	1	gain	0	
1	18554400	19639691	2	1	neutral	0	
1	19644100	19655121	5	2	gain	0	
1	19666030	21900261	2	1	neutral	0	
1	21900540	22074731	4	2	gain	0	
1	22078000	29563211	2	1	neutral	0	
1	29581790	29652161	4	0	gain	1	
1	31186430	31351571	2	1	neutral	0	
1	31406060	31538621	4	2	gain	0	
1	31654550	32165701	2	1	neutral	0	
1	32166990	32222861	4	2	gain	0	
1	32229490	33100361	2	1	neutral	0	
1	33116030	33145743	4	0	gain	1	
1	33147390	36065851	2	1	neutral	0	
1	36066100	36107301	6	0	gain	1	
1	36181240	36282641	2	1	neutral	0	
1	36288460	36557661	4	2	gain	0	
1	36558030	39395271	2	1	neutral	0	
1	39457060	39500161	5	0	gain	1	
1	39549890	40537181	2	1	neutral	0	
1	40539740	40627281	4	0	gain	1	
1	40654730	44044581	2	1	neutral	0	
1	44048760	44063721	4	0	gain	1	
1	44064390	44451291	2	1	neutral	0	
1	44455970	44456111	4	0	gain	1	

1	44457550	46651201	2	1	neutral	0	
1	46654390	46663181	10	0	gain	1	POMGNT1
1	46663380	46743951	4	0	gain	1	
1	46744540	48705201	2	1	neutral	0	
1	48708130	51425481	3	1	gain	0	
1	51436030	51443961	5	0	gain	1	
1	51578140	55474291	2	1	neutral	0	
1	55505510	55529251	4	2	gain	0	
1	55534720	70904801	2	1	neutral	0	
1	71318530	71531411	4	2	gain	0	
1	71532460	97217031	2	1	neutral	0	
1	97235260	97278931	5	0	gain	1	
1	97544530	108771751	2	1	neutral	0	
1	108992890	108994871	6	0	gain	1	
1	109010080	150297501	2	1	neutral	0	
1	150298210	150325411	4	0	gain	1	
1	150337190	150980741	2	1	neutral	0	
1	150981110	151006691	5	0	gain	1	PRUNE1
1	151009210	167097841	2	1	neutral	0	
1	167190140	167385041	5	0	gain	1	POU2F1
1	167400920	170521561	2	1	neutral	0	
1	170633360	170705291	6	0	gain	1	
1	170914700	171310961	2	1	neutral	0	
1	171481230	171560981	4	0	gain	1	
1	171605070	173921271	2	1	neutral	0	
1	173930220	174992511	4	2	gain	0	
1	175046560	176525961	2	1	neutral	0	
1	176563660	176998871	5	2	gain	0	
1	176999950	177133831	6	0	gain	1	
1	177199020	196716441	2	1	neutral	0	
1	196744020	196801121	1	0	loss	1	
1	196857280	202113681	2	1	neutral	0	
1	202117740	202128911	5	0	gain	1	
1	202129680	205053220	2	1	neutral	0	
1	205057920	205091001	4	0	gain	1	RBBP5
1	205116690	213174231	2	1	neutral	0	
1	213178380	213189011	4	0	gain	1	
1	213224750	214510101	2	1	neutral	0	
1	214531270	214638131	5	0	gain	1	PTPN14
1	214787100	228525051	2	1	neutral	0	
1	228525650	228526041	1	0	loss	1	
1	228526560	233113961	2	1	neutral	0	
1	233120050	233431181	4	2	gain	0	
1	233463780	242162361	2	1	neutral	0	
1	242253160	244552361	3	1	gain	0	

1	244572890	247007201	2	1	neutral	0	
1	247012920	247320701	3	1	gain	0	
1	247322310	249212561	2	1	neutral	0	
2	41610	1643181	2	1	neutral	0	
2	1647140	1691461	5	0	gain	1	
2	1695700	20451451	2	1	neutral	0	
2	20453590	20647731	5	2	gain	0	
2	20818440	27354341	2	1	neutral	0	
2	27354540	27356551	5	0	gain	1	
2	27357160	27357411	7	0	gain	1	
2	27359930	43937681	2	1	neutral	0	
2	43937980	43939521	6	0	gain	1	
2	43947810	111334581	2	1	neutral	0	
2	111395540	112670601	4	2	gain	0	
2	112686700	136262051	2	1	neutral	0	
2	136289110	136481861	5	0	gain	1	
2	136499500	141571371	2	1	neutral	0	
2	141598480	141680721	0	0	loss	1	LRP1B segm
2	141707800	179170971	2	1	neutral	0	
2	179184970	179320841	4	0	gain	1	
2	179323240	209116251	2	1	neutral	0	
2	209136250	210543401	4	2	gain	0	
2	210545480	215013921	2	1	neutral	0	
2	215013970	217006011	3	1	gain	0	
2	217012810	242841471	2	1	neutral	0	
3	361460	10362293	2	1	neutral	0	
3	10362700	12660211	3	1	gain	0	ATP2B2, ATG7, RAF1
3	12776280	16535341	2	1	neutral	0	
3	16630170	16640061	1	0	loss	1	
3	16645950	24231821	2	1	neutral	0	
3	25215890	25638101	5	0	gain	1	
3	25639800	46666671	2	1	neutral	0	
3	46712480	46759121	3	1	gain	0	
3	46759210	49950761	2	1	neutral	0	
3	49967050	50155871	4	0	gain	1	
3	50197060	51517841	2	1	neutral	0	
3	51624440	51697431	4	0	gain	1	
3	51708320	63898891	2	1	neutral	0	
3	63938060	63996611	3	0	gain	1	
3	63999120	64009231	5	0	gain	1	PSMD6
3	64009480	64009511	15	0	gain	1	PSMD6 1 fr
3	64084730	64673321	4	0	gain	1	
3	65342060	74570211	2	1	neutral	0	

3	74570260	87302971	3	1	gain	0	
3	87309050	87325571	5	0	gain	1	
3	88039900	122186271	2	1	neutral	0	
3	122215290	122354911	3	1	gain	0	
3	122399730	133114811	2	1	neutral	0	
3	133118930	133191391	5	0	gain	1	BFSP2
3	133193812	142145641	2	1	neutral	0	RASA2
3	142151510	142297531	4	2	gain	0	ATR
3	142383080	197896711	2	1	neutral	0	
4	53382	59431	1	0	loss	1	
4	59950	7765511	2	1	neutral	0	
4	7770570	8129381	4	2	gain	0	
4	8160410	57193961	2	1	neutral	0	
4	57204570	57308021	3	1	gain	0	
4	57312860	73154571	2	1	neutral	0	
4	73156580	74124501	4	2	gain	0	
4	74270050	85719251	2	1	neutral	0	
4	85722810	87735661	4	2	gain	0	
4	87744840	99325731	2	1	neutral	0	
4	99337900	99363241	5	0	gain	1	
4	99393680	123075481	2	1	neutral	0	
4	123075530	123342471	3	1	gain	0	
4	123342520	160264541	2	1	neutral	0	
4	160265190	160279291	6	0	gain	1	
4	162306900	190947591	2	1	neutral	0	
5	140430	741741	2	1	neutral	0	
5	745300	755171	1	0	loss	1	
5	756080	5140633	2	1	neutral	0	
5	5140780	5319241	4	2	gain	0	
5	5423030	34954081	2	1	neutral	0	
5	34954660	35118221	4	2	gain	0	
5	35618100	45645691	2	1	neutral	0	
5	45695770	49724551	1	0	loss	1	
5	49736880	55272101	2	1	neutral	0	
5	55396010	55528731	3	1	gain	0	
5	56111400	56111851	1	0	loss	1	
5	56112890	64447771	2	1	neutral	0	
5	64466450	64850731	4	2	gain	0	
5	64859140	64883221	6	3	gain	0	PPWD1
5	64886130	64926531	3	1	gain	0	
5	64930740	72849281	2	1	neutral	0	
5	72850130	72858711	5	0	gain	1	
5	72863170	73200081	3	1	gain	0	

5	73200190	73236821	5	2	gain	0	
5	73930540	80171661	2	1	neutral	0	
5	80256560	80497251	5	2	gain	0	
5	80502670	86670131	2	1	neutral	0	
5	86670660	86675661	6	0	gain	1	
5	86676330	92956751	3	1	gain	0	
5	92956800	130726741	2	1	neutral	0	
5	130762960	130831351	4	2	gain	0	
5	130834140	131326651	3	0	gain	1	
5	131329730	131895061	2	1	neutral	0	
5	131911460	131978021	4	0	gain	1	
5	131993880	139752331	2	1	neutral	0	
5	139781560	139928841	3	0	gain	1	
5	139930290	150056421	2	1	neutral	0	
5	150071320	150078161	5	0	gain	1	
5	150078810	167896011	2	1	neutral	0	
5	167913510	167927701	5	2	gain	0	
5	167929010	175387131	2	1	neutral	0	
5	175388280	175395201	5	2	gain	0	
5	175520200	180687511	2	1	neutral	0	
6	292540	3739351	2	1	neutral	0	
6	3751510	3751761	1	0	loss	1	
6	3850050	4060841	5	0	gain	1	
6	4068930	13621041	2	1	neutral	0	
6	13622600	13697121	5	0	gain	1	
6	13711170	26094451	2	1	neutral	0	
6	26104180	27861591	3	1	gain	0	
6	27879030	31584671	2	1	neutral	0	
6	31590570	31625461	4	2	gain	0	
6	31625840	31962461	2	1	neutral	0	
6	31963480	31965561	4	0	gain	1	
6	31994710	31997130	1	0	loss	1	
6	31997340	41884671	2	1	neutral	0	
6	41888750	41900401	4	0	gain	1	
6	41903670	43042401	2	1	neutral	0	
6	43044230	43098071	3	0	gain	1	
6	43098120	43127681	4	0	gain	1	
6	43128460	43146601	3	0	gain	1	
6	43146840	43337061	2	1	neutral	0	
6	43395720	43422201	3	1	gain	0	
6	43422490	123046331	2	1	neutral	0	
6	123100940	123110601	5	2	gain	0	
6	123110960	166578151	2	1	neutral	0	incl QKI
6	166578290	166736371	1	0	loss	1	

6	166738030	170893641	2	1	neutral	0	
7	193200	6694241	3	1	gain	0	
7	6730480	6737421	4	0	gain	1	
7	6744790	6844671	7	3	gain	0	
7	6845640	20434521	3	1	gain	0	
7	20434570	20666231	4	0	gain	1	
7	20668310	20721281	7	0	gain	1	
7	20725320	20795241	6	2	gain	0	
7	20823910	21985421	3	1	gain	0	
7	22162030	22349691	6	0	gain	1	
7	22354200	44621381	3	1	gain	0	
7	44621650	44621801	11	0	gain	1	TMED4 segm
7	44663950	47698811	3	1	gain	0	
7	47814740	47971641	6	2	gain	0	
7	47976450	48237941	3	1	gain	0	
7	48258950	48666651	6	2	gain	0	
7	48682890	65445361	3	1	gain	0	
7	65446960	66221061	4	2	gain	0	
7	66236870	66274231	6	0	gain	1	
7	66367530	94791281	3	1	gain	0	PPP1R9A
7	94827660	94918001	9	1	gain	0	PPP1R9A
7	94919410	102119361	3	1	gain	0	
7	102125490	102312041	8	3	gain	0	RASA4B POLR2J3
7	102389660	106793681	3	1	gain	0	
7	106797430	106810571	7	0	gain	1	2 genes segm
7	106814900	121616281	3	1	gain	0	
7	121616840	121701241	8	3	gain	0	PTPRZ1 25/30 segm
7	121716550	157208761	3	1	gain	0	
7	157333410	158334461	6	3	gain	0	PTPRN2
7	158380250	158937421	3	1	gain	0	
8	190900	25902351	2	1	neutral	0	
8	26149340	26217791	4	2	gain	0	
8	26218490	26265851	5	0	gain	1	
8	26267880	27327281	3	1	gain	0	
8	27327330	37611591	2	1	neutral	0	
8	37620076	37635591	5	0	gain	1	
8	37654790	48650381	2	1	neutral	0	
8	48686740	48872681	4	2	gain	0	
8	48873710	53580731	2	1	neutral	0	
8	53586410	53598011	5	2	gain	0	
8	53852470	110099751	2	1	neutral	0	
8	110100250	119964051	3	1	gain	0	
8	119964052	141445311	2	1	neutral	0	

8	141449150	141935811	4	2	gain	0	PTK2/FAK
8	142138810	146279511	2	1	neutral	0	
9	14810	15465571	2	1	neutral	0	
9	15466750	15472541	6	0	gain	1	
9	15472630	21941011	2	1	neutral	0	
9	21968220	21994431	0	0	loss	1	CDKN2A
9	22005980	38424221	2	1	neutral	0	
9	38543250	43885051	3	1	gain	0	
9	43891500	69403191	4	2	gain	0	
9	69416140	70913981	4	1	gain	0	
9	70917870	74477521	2	1	neutral	0	
9	74480070	74489981	4	0	gain	1	
9	74526650	80881861	2	1	neutral	0	
9	80912130	80943071	4	2	gain	0	
9	80943900	98011551	2	1	neutral	0	
9	98209190	98270941	4	2	gain	0	PTCH1
9	98278740	112082671	2	1	neutral	0	
9	112082720	112082721	1	0	loss	1	
9	112141840	112207571	5	0	gain	1	PTPN3
9	112211090	112930771	4	2	gain	0	PTPN3
9	112963230	114306711	2	1	neutral	0	
9	114312060	114359701	4	2	gain	0	
9	114393690	125719481	2	1	neutral	0	
9	125746770	125783181	6	0	gain	1	
9	125796850	125865471	3	0	gain	1	
9	125884600	127905711	2	1	neutral	0	
9	127911960	127996221	4	2	gain	0	
9	127998870	132404471	2	1	neutral	0	
9	132481510	132515271	4	0	gain	1	
9	132565500	133320581	2	1	neutral	0	
9	133325660	133374951	4	0	gain	1	
9	133376370	133540241	2	1	neutral	0	
9	133542000	133569281	5	2	gain	0	PRDM12
9	133570880	134371231	2	1	neutral	0	
9	134379610	134387489	4	2	gain	0	
9	134388630	134612901	3	1	gain	0	
9	134735930	139347881	2	1	neutral	0	
9	139347930	139728681	2	1	neutral	0	
9	139730200	139734301	5	2	gain	0	RABL6 segm
9	139734610	141111751	2	1	neutral	0	
10	93000	27355471	1	0	loss	1	
10	27356090	27389221	3	0	gain	1	ANKRD26 3'
10	27400910	47756091	1	0	loss	1	

10	47909170	47948821	0	0	loss	1	FAM21B
10	48235820	51130441	1	0	loss	1	
10	51130600	56138701	2	1	neutral	0	
10	56287580	69991381	1	0	loss	1	
10	69991430	70182331	2	1	neutral	0	
10	70182460	88492701	1	0	loss	1	
10	88635780	88683451	3	0	gain	1	BMPR1A
10	88696500	93668451	1	0	loss	1	incl PTEN
10	93683840	93787041	2	1	neutral	0	BTAF1
10	93788550	102987531	1	0	loss	1	
10	102988250	102988551	0	0	loss	1	LBX1 fr
10	103113940	118187571	1	0	loss	1	
10	118196230	118225671	3	0	gain	1	PNLIPRP3 fr
10	118228700	135440221	1	0	loss	1	incl FGFR2, DMBT1
11	193100	1411571	2	1	neutral	0	
11	1431680	1482181	3	1	gain	0	
11	1491320	17565851	2	1	neutral	0	
11	17568920	17667491	3	1	gain	0	
11	17741330	20385861	2	1	neutral	0	
11	20388720	20529951	3	0	gain	1	
11	20621220	22225391	2	1	neutral	0	
11	22232810	22296271	4	2	gain	0	
11	22297640	30255321	3	1	gain	0	
11	30352500	30358321	4	2	gain	0	
11	30431620	47436891	2	1	neutral	0	
11	47440390	47470481	4	0	gain	1	
11	47493790	48131621	2	1	neutral	0	
11	48134300	48186041	4	0	gain	1	
11	48188760	48188911	6	0	gain	1	
11	48238370	64444501	2	1	neutral	0	
11	64453120	64514271	3	1	gain	0	
11	64514400	64527231	4	0	gain	1	
11	64527280	64527331	4	0	gain	1	
11	64532960	65380991	2	1	neutral	0	
11	65383790	65404441	3	0	gain	1	
11	65408400	66373321	2	1	neutral	0	
11	66384200	66412211	5	0	gain	1	
11	66413500	69924811	2	1	neutral	0	
11	69931520	70228251	4	2	gain	0	
11	70253410	82444771	2	1	neutral	0	
11	82535950	82611431	5	0	gain	1	PRCP
11	82625780	82997221	3	1	gain	0	
11	83170860	83252881	1	0	loss	1	
11	83344260	89607421	2	1	neutral	0	

11	89608150	89608201	1	0	loss	1	
11	89608780	89609181	3	1	gain	0	
11	89701680	89773091	11	5	gain	0	TRIM64; TRIM49C 2/3
11	89774220	103349981	2	1	neutral	0	
11	103780430	103866951	4	2	gain	0	
11	103870780	111594721	2	1	neutral	0	
11	111597690	111657221	4	2	gain	0	
11	111680370	112088591	2	1	neutral	0	
11	112097170	112131281	5	0	gain	1	
11	113073100	114262351	2	1	neutral	0	
11	114270630	114310391	4	0	gain	1	
11	114311380	129762861	2	1	neutral	0	
11	129762960	129830841	4	2	gain	0	
11	129939880	134257701	2	1	neutral	0	
12	176050	9268411	2	1	neutral	0	
12	9301570	9555186	4	2	gain	0	
12	9681850	9711821	7	0	gain	1	
12	9711870	9711921	6	0	gain	1	
12	9712710	24982811	2	1	neutral	0	
12	24985660	25101861	5	2	gain	0	
12	25147230	49393061	2	1	neutral	0	
12	49396690	49412801	5	2	gain	0	
12	49415560	50368861	2	1	neutral	0	
12	50369360	50384571	3	0	gain	1	
12	50385780	50452901	5	0	gain	1	
12	50453550	50475431	4	2	gain	0	
12	50478780	51138621	2	1	neutral	0	
12	51173930	51208221	6	2	gain	0	
12	51213420	53901251	2	1	neutral	0	
12	53910920	54069941	4	0	gain	1	
12	54105730	56881871	2	1	neutral	0	
12	56915810	56982131	6	0	gain	1	
12	56982720	57006951	4	0	gain	1	
12	57007000	57081841	4	2	gain	0	
12	57106330	57146081	3	1	gain	0	
12	57167640	70824421	2	1	neutral	0	
12	70915260	71314161	4	2	gain	0	
12	71519120	80192331	2	1	neutral	0	
12	80199380	82752601	4	2	gain	0	
12	82780590	88926271	2	1	neutral	0	
12	88939530	93171921	3	1	gain	0	
12	93172890	108128291	2	1	neutral	0	
12	108133160	108154291	5	0	gain	1	
12	108169000	110760851	2	1	neutral	0	

12	110764200	110780241	5	0	gain	1	
12	110781040	110815631	4	0	gain	1	
12	110819560	111886081	2	1	neutral	0	
12	111890620	111923071	4	0	gain	1	
12	111923120	112123591	5	2	gain	0	
12	112130520	112193531	3	0	gain	1	
12	112194020	121014451	2	1	neutral	0	
12	121017120	121019161	5	0	gain	1	
12	121078490	122460051	2	1	neutral	0	
12	122468610	122497051	5	2	gain	0	
12	122516690	123353081	2	1	neutral	0	
12	123355440	123645331	3	0	gain	1	
12	123645380	133810901	2	1	neutral	0	
13	19748010	32885911	2	1	neutral	0	
13	32890590	32976321	5	2	gain	0	BRCA2
13	32976570	52440021	2	1	neutral	0	
13	52508890	52602711	4	2	gain	0	
13	52602940	115091751	2	1	neutral	0	
14	19377600	20007621	6	2	gain	0	
14	20010110	23371901	2	1	neutral	0	
14	23372270	23398681	4	0	gain	1	
14	23415740	25518921	2	1	neutral	0	
14	26917170	30194851	4	0	gain	1	
14	30396460	35270391	2	1	neutral	0	
14	35272060	35343801	5	0	gain	1	
14	35465920	38725221	2	1	neutral	0	
14	39502450	47601021	1	0	loss	1	
14	47613280	51371081	2	1	neutral	0	
14	51372110	51411081	6	0	gain	1	PYGL
14	51444010	60337311	2	1	neutral	0	
14	60574360	60585401	5	2	gain	0	
14	60587920	61435301	2	1	neutral	0	
14	61441830	65198441	1	0	loss	1	
14	65198490	65209171	0	0	loss	1	PLEKHG3 fr
14	65209700	67878771	1	0	loss	1	
14	67940140	70125411	2	1	neutral	0	
14	70170120	75269361	1	0	loss	1	
14	75276090	75282961	2	1	neutral	0	
14	75283280	77737251	1	0	loss	1	
14	77743720	77787521	4	1	gain	0	POMT2
14	77787710	77910651	1	0	loss	1	
14	77914840	77931991	2	1	neutral	0	
14	77934430	81686861	1	0	loss	1	

14	81728130	92530721	2	1	neutral	0	
14	92537090	92572891	4	2	gain	0	
14	92582540	102229301	2	1	neutral	0	
14	102252360	102376021	5	0	gain	1	
14	102378740	105361251	2	1	neutral	0	
14	105393460	105399261	5	2	gain	0	
14	105404400	105996131	2	1	neutral	0	
15	20739500	22074701	2	1	neutral	0	
15	22368580	22538521	0	0	loss	1	OR4M2; OR4N4
15	22742400	40954381	2	1	neutral	0	
15	40990960	41023361	5	0	gain	1	
15	41028740	42264661	2	1	neutral	0	
15	42275960	42447771	3	0	gain	1	
15	42448640	66048781	2	1	neutral	0	
15	66161790	66180141	5	0	gain	1	
15	66190280	76998301	2	1	neutral	0	
15	77020940	77578821	3	1	gain	0	
15	77623260	77697101	5	2	gain	0	
15	77709640	77711921	3	1	gain	0	
15	77750750	78933471	2	1	neutral	0	
15	79051800	80253511	3	1	gain	0	
15	80259980	80263451	6	0	gain	1	
15	80412690	81046701	4	2	gain	0	
15	81166220	91185371	2	1	neutral	0	
15	91290620	91358481	5	0	gain	1	
15	91418970	91504991	2	1	neutral	0	
15	91510360	91537641	4	2	gain	0	
15	91542210	102462851	2	1	neutral	0	
16	97430	103581	3	0	gain	1	
16	103990	640401	1	0	loss	1	
16	667210	677601	3	0	gain	1	
16	681260	1524941	1	0	loss	1	
16	1535940	2201611	2	1	neutral	0	
16	2201700	2203421	3	0	gain	1	
16	2213930	2240351	2	1	neutral	0	
16	2255690	2256401	4	0	gain	1	
16	2256480	2263301	2	1	neutral	0	
16	2263730	2273921	3	0	gain	1	
16	2278380	2550951	2	1	neutral	0	
16	2564110	2579731	4	0	gain	1	
16	2580260	3019831	2	1	neutral	0	
16	3021160	3021921	4	0	gain	1	
16	3022640	3740981	2	1	neutral	0	

16	3777710	5290091	1	0	loss	1	
16	5517220	11363111	2	1	neutral	0	
16	11367140	11375091	4	0	gain	1	
16	11439330	14766571	2	1	neutral	0	
16	14818880	14966141	3	0	gain	1	
16	14966180	20364051	2	1	neutral	0	
16	20370640	20396171	4	2	gain	0	
16	20410420	20451721	2	1	neutral	0	
16	20471440	20638611	4	0	gain	1	
16	20648060	21771841	2	1	neutral	0	
16	21846960	27556841	1	0	loss	1	
16	27560990	28403451	2	1	neutral	0	
16	28488840	29997181	1	0	loss	1	
16	29997590	30208641	2	1	neutral	0	
16	30212050	47733241	1	0	loss	1	
16	48117630	48278501	2	0	neutral	1	
16	48286050	69988491	1	0	loss	1	
16	69996880	70220421	2	1	neutral	0	
16	70284990	71971301	1	0	loss	1	
16	71976470	72043041	3	0	gain	1	
16	72045950	89785491	1	0	loss	1	
16	89786850	89788251	0	0	loss	1	ZNF276 fr
16	89788940	90142301	1	0	loss	1	
17	6010	1554241	2	1	neutral	0	CRK
17	1554410	1583061	3	0	gain	1	
17	1584020	1587821	5	0	gain	1	
17	1611070	3828731	2	1	neutral	0	
17	3830130	3856691	4	2	gain	0	
17	3856990	4060361	2	1	neutral	0	
17	4071080	4167231	3	1	gain	0	
17	4186130	7134101	2	1	neutral	0	
17	7137390	7147521	4	0	gain	1	
17	7147870	8079571	2	1	neutral	0	TP53
17	8092390	8113541	4	2	gain	0	
17	8131500	8702431	2	1	neutral	0	
17	8706590	8753111	4	0	gain	1	
17	8783960	19813291	2	1	neutral	0	
17	19823350	19880961	5	2	gain	0	
17	19999970	27233501	2	1	neutral	0	
17	27233880	27278421	4	0	gain	1	
17	27282970	27332881	2	1	neutral	0	
17	27370250	27417931	6	0	gain	1	
17	27419360	27426761	3	1	gain	0	
17	27430620	28576181	2	1	neutral	0	

17	28593910	28618521	4	0	gain	1	
17	28618830	30696771	2	1	neutral	0	
17	30771550	30807611	5	0	gain	1	
17	30814640	40575101	2	1	neutral	0	
17	40612920	40665981	4	0	gain	1	
17	40666310	40961541	2	1	neutral	0	
17	40962780	40993571	4	0	gain	1	
17	40996650	56770151	2	1	neutral	0	
17	56772290	56811571	6	0	gain	1	RAD51C
17	56833360	57208701	2	1	neutral	0	PPM1E
17	57232320	57275111	5	0	gain	1	
17	57278930	58603261	2	1	neutral	0	
17	58677780	58740861	5	0	gain	1	PPM1D
17	58756820	64222151	2	1	neutral	0	
17	64222200	65822431	3	1	gain	0	incl PRKCA
17	65850060	65862781	5	0	gain	1	
17	65870940	65924711	4	0	gain	1	
17	65925080	65944401	5	0	gain	1	
17	65955660	79639731	2	1	neutral	0	
17	79648740	79650831	5	0	gain	1	
17	79651100	79881061	2	0	neutral	1	
17	79890760	79895081	5	0	gain	1	PYCR1
17	79898700	81052301	2	1	neutral	0	
18	158700	7567871	2	0	neutral	1	
18	7774150	11610611	3	0	gain	1	incl PTPRM
18	11689570	14764081	2	0	neutral	1	
18	14769350	14848921	1	0	loss	1	
18	14850220	43417051	2	0	neutral	1	
18	43417480	43419041	1	0	loss	1	
18	43420180	48466711	2	0	neutral	1	
18	48466750	55283201	3	0	gain	1	
18	55287800	55328661	5	0	gain	1	
18	55329720	78005211	2	0	neutral	1	
19	281390	1497201	2	1	neutral	0	
19	1506020	1535221	4	2	gain	0	
19	1555330	1650231	2	1	neutral	0	
19	1775160	1775411	1	0	loss	1	
19	1783030	5151501	2	1	neutral	0	
19	5206790	5286161	4	0	gain	1	
19	5455510	17389851	2	1	neutral	0	
19	17392570	17416761	4	2	gain	0	
19	17416980	17533241	3	1	gain	0	
19	17534310	33349441	2	1	neutral	0	

19	33350750	33370271	1	0	loss	1	
19	33372750	36038121	2	1	neutral	0	
19	36038230	36103751	4	0	gain	1	
19	36104640	36602311	2	1	neutral	0	
19	36603670	36606571	4	2	gain	0	
19	36606950	38861421	2	1	neutral	0	
19	38865250	38874001	5	0	gain	1	
19	38875060	39522861	2	1	neutral	0	
19	39575910	39600751	4	0	gain	1	
19	39660200	40477141	2	1	neutral	0	
19	40478060	40487201	4	0	gain	1	
19	40504240	41712391	2	1	neutral	0	
19	41725300	41727151	3	0	gain	1	
19	41727790	41765801	5	2	gain	0	
19	41770410	41898881	2	1	neutral	0	
19	41903090	41930501	4	2	gain	0	
19	41931490	42414221	2	1	neutral	0	
19	42417010	42498221	3	1	gain	0	
19	42507490	43699418	2	1	neutral	0	
19	43702150	43763261	1	0	loss	1	
19	43766020	46191801	2	1	neutral	0	
19	46195150	46206271	6	3	gain	0	
19	46214950	47177981	2	1	neutral	0	
19	47178290	47225301	4	0	gain	1	
19	47225500	49477981	2	1	neutral	0	
19	49481180	49551621	2	0	neutral	1	
19	49551990	49621621	2	1	neutral	0	
19	49621890	49654581	4	2	gain	0	
19	49654780	51227821	2	1	neutral	0	
19	51228230	51298401	3	1	gain	0	
19	51301360	52671361	2	1	neutral	0	
19	52693350	52772881	4	0	gain	1	
19	52780410	54725461	2	1	neutral	0	
19	54725630	54745861	1	0	loss	1	
19	54746020	55597311	2	1	neutral	0	
19	55597430	55624141	3	1	gain	0	
19	55624760	55624961	4	0	gain	1	
19	55644290	55758451	3	1	gain	0	
19	55773740	59083971	2	1	neutral	0	
20	68350	2847181	2	1	neutral	0	
20	2903910	2945711	6	0	gain	1	PTPRA segm
20	2945759	3016581	5	2	gain	0	PTPRA segm
20	3017800	3515952	2	1	neutral	0	
20	3520870	3520971	3	0	gain	1	

20	3526410	3571921	5	2	gain	0	
20	3573110	3662661	3	1	gain	0	
20	3669210	20350441	2	1	neutral	0	
20	20373750	20661441	5	0	gain	1	RALGAPA2
20	20693020	35575191	2	1	neutral	0	
20	35579840	35724331	5	0	gain	1	
20	35731090	36874521	2	1	neutral	0	
20	36888900	36965581	5	2	gain	0	
20	36974920	37063991	2	1	neutral	0	
20	37117080	37182711	5	0	gain	1	
20	37186940	37464811	3	1	gain	0	
20	37518240	37547301	5	0	gain	1	
20	37555000	40162191	2	1	neutral	0	
20	40179950	40727111	4	0	gain	1	
20	40727160	41514551	4	2	gain	0	
20	42086680	42143381	3	0	gain	1	
20	42143650	46386091	2	1	neutral	0	
20	47242430	47364401	5	2	gain	0	
20	47444180	47649711	3	1	gain	0	
20	47675000	50140641	2	1	neutral	0	
20	50158910	50159251	0	0	loss	1	NFATC2 1fr
20	50179100	55213041	2	1	neutral	0	
20	55746020	55841161	5	0	gain	1	
20	55904930	55918501	2	1	neutral	0	
20	55926230	55982901	5	0	gain	1	
20	56072230	62904931	2	1	neutral	0	
21	10906910	46237881	2	1	neutral	0	
21	46271290	46293921	4	0	gain	1	PTTG1IP
21	46306290	48084261	2	1	neutral	0	
22	16266930	19951791	1	0	loss	1	
22	19954500	19978311	2	1	neutral	0	
22	20024330	20307511	1	0	loss	1	
22	20377850	20380321	20	7	gain	0	TMEM191B
22	20456390	24530371	1	0	loss	1	
22	24560370	24765281	2	1	neutral	0	
22	24807560	26317371	1	0	loss	1	
22	26342110	26423641	2	1	neutral	0	
22	26565640	38710141	1	0	loss	1	
22	38822800	38889721	2	1	neutral	0	
22	38889770	38934401	3	0	gain	1	
22	38934550	41636891	1	0	loss	1	
22	41642610	41681651	2	1	neutral	0	RANGAP1
22	41716670	45122511	1	0	loss	1	

22	45125200	45133111	5	1	gain	0	PRR5-ARHGAP8 segm
22	45182340	51183581	1	0	loss	1	
22	51207210	51220721	3	0	gain	1	RABL2B
X	295060	2799251	2	1	neutral	0	
X	2825320	3030571	4	2	gain	0	
X	3227760	18690181	2	1	neutral	0	
X	18725900	18845601	5	0	gain	1	
X	18911610	76711971	2	1	neutral	0	
X	76763830	77302041	3	0	gain	1	
X	77359840	152752241	2	1	neutral	0	
X	152770090	152845741	3	1	gain	0	
X	152853830	155004401	2	1	neutral	0	
Y	21154300	21154641	3	0	gain	1	
